# Bonnevillamides, Linear Heptapeptides Isolated from a Great Salt Lake-Derived *Streptomyces* sp.

**DOI:** 10.3390/md15070195

**Published:** 2017-06-24

**Authors:** Guangwei Wu, Jason R. Nielson, Randall T. Peterson, Jaclyn M. Winter

**Affiliations:** 1 Department of Medicinal Chemistry, University of Utah, Salt Lake City, UT 84112, USA; Guangwei.Wu@utah.edu; 2 Department of Pharmacology and Toxicology, University of Utah, Salt Lake City, UT 84112, USA; Jason.Nielson@pharm.utah.edu (J.R.N); Randall.Peterson@pharm.utah.edu (R.T.P.)

**Keywords:** Great Salt Lake, hypersaline environment, heptapeptide, azetidine, *Streptomyces* sp., zebrafish

## Abstract

*Streptomyces* sp. GSL-6B was isolated from sediment collected from the Great Salt Lake and investigation of its organic extract led to the isolation of three new linear heptapeptides, bonnevillamides A (**1**), B (**2**), and C (**3**). The bonnevillamides represent a new class of linear peptides featuring unprecedented non-proteinogenic amino acids. All three peptides contain the newly characterized bonnevillic acid moiety (3-(3,5-dichloro-4-methoxyphenyl)-2-hydroxyacrylic acid), as well as a heavily modified proline residue. Moreover, in bonnevillamide A, the terminal proline residue found in bonnevillamides B and C is replaced with 4-methyl-azetidine-2-carboxylic acid methyl ester. The structures of the three heptapeptides were elucidated by NMR, high-resolution electrospray ionization mass spectroscopy (HRESIMS), and LC-MS/MS, and the absolute configuration of all proteinogenic amino acid residues were determined by advanced Marfey’s method. Bonnevillamides A, B and C were evaluated for their effects on zebrafish embryo development. All three heptapeptides were shown to modulate heart growth and cardiac function, with bonnevillamide B having the most pronounced effect.

## 1. Introduction

Natural products are medically useful compounds that play a pivotal role in drug discovery programs. However, with the increasing demand for new therapeutic agents, the discovery of new natural product scaffolds possessing novel modes of action are urgently needed. Biological pressures can influence the chemical diversity of secondary metabolites and microorganisms isolated from extreme environments, such as the deep sea [1], salterns [2,3], and hydrothermal vents [4,5] have been shown to be excellent producers of bioactive and structurally diverse small molecules [6]. Great Salt Lake, located in Utah, USA, is one of the world’s most hypersaline environments. Seawater has an average salinity of ~3.3%, whereas the Great Salt Lake ranges between 5% and 27% [7]. It has been reported that the Great Salt Lake is rich in halophilic and halotolerant microbial communities [7,8,9,10]. However, while there has been much success at discovering novel phylotypes, little is known about the Great Salt Lake’s potential as a resource for small molecule discovery.

**Figure 1:**
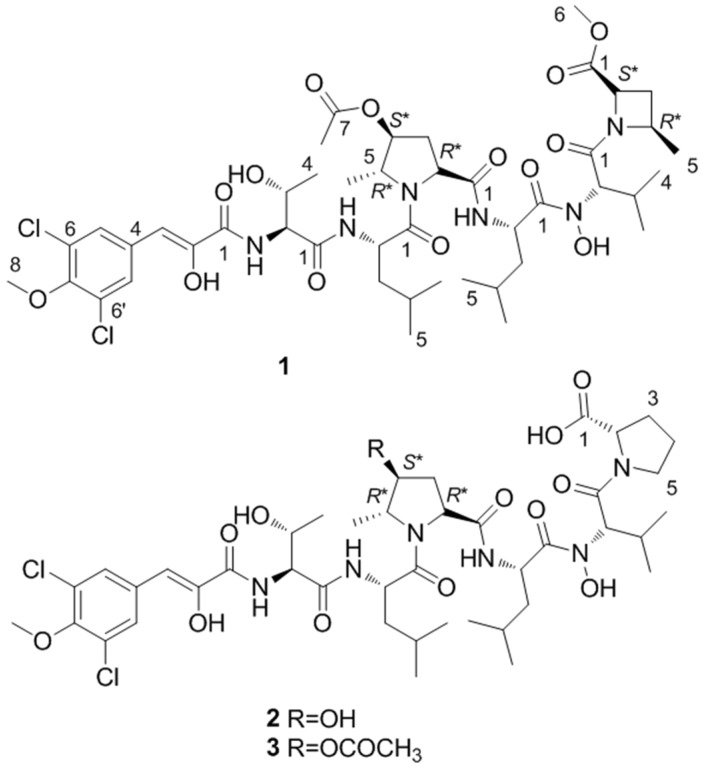
Structures of bonnevillamide A (**1**), B (**2**) and C (**3**).

Our current interest is aimed at evaluating the potential of halophilic and halotolerant bacteria isolated from the Great Salt Lake as new resources for the discovery of biologically active natural products. Sediment was collected from various locations in Farmington Bay, which is located in between Great Salt Lake’s North and South Arms, and processed to enrich for bacteria belonging to the order actinomycetes. HPLC and LC–MS were used to screen for secondary metabolites and it was observed that a new strain, *Streptomyces* sp. GSL-6B, produced a class of dichloro-substituted metabolites with a unique UV spectrum. Guided by UV characteristics and molecular weight, three linear heptapeptides were isolated. The bonnevillamides A–C (**1**–**3**) represent a new class of linear heptapeptides containing unprecedented amino acid residues (Figure 1). The three bonnevillamides contain bonnevillic acid (BVA) (3-(3,5-dichloro-4-methoxyphenyl)-2-hydroxyacrylic acid) and a heavily modified proline residue. Additionally, in place of a terminal proline residue, as observed in bonnevillamides B and C, bonnevillamide A contains 4-methyl-azetidine-2-carboxylic acid methyl ester (MACME). Herein, we report the isolation, structure elucidation and biological activity of **1**–**3**.

## 2. Results and Discussion

After a nine day fermentation, *Streptomyces* sp. GSL-6B was harvested by centrifugation. Diaion HP-20 resin was incubated with the supernatant and then extracted with methanol to yield the crude supernatant extract, whereas the mycelium was extracted with acetone/water (70%/30%) to produce the mycelium crude extract. Using LC–MS, bonnevillamides A–C were detected in both the supernatant and mycelium extracts. The organic extracts were combined and fractionated by Sephadex LH-20 column chromatography and semi-preparative HPLC to afford compounds **1**–**3**.

### 2.1. Structure Elucidation

**Figure 2:**
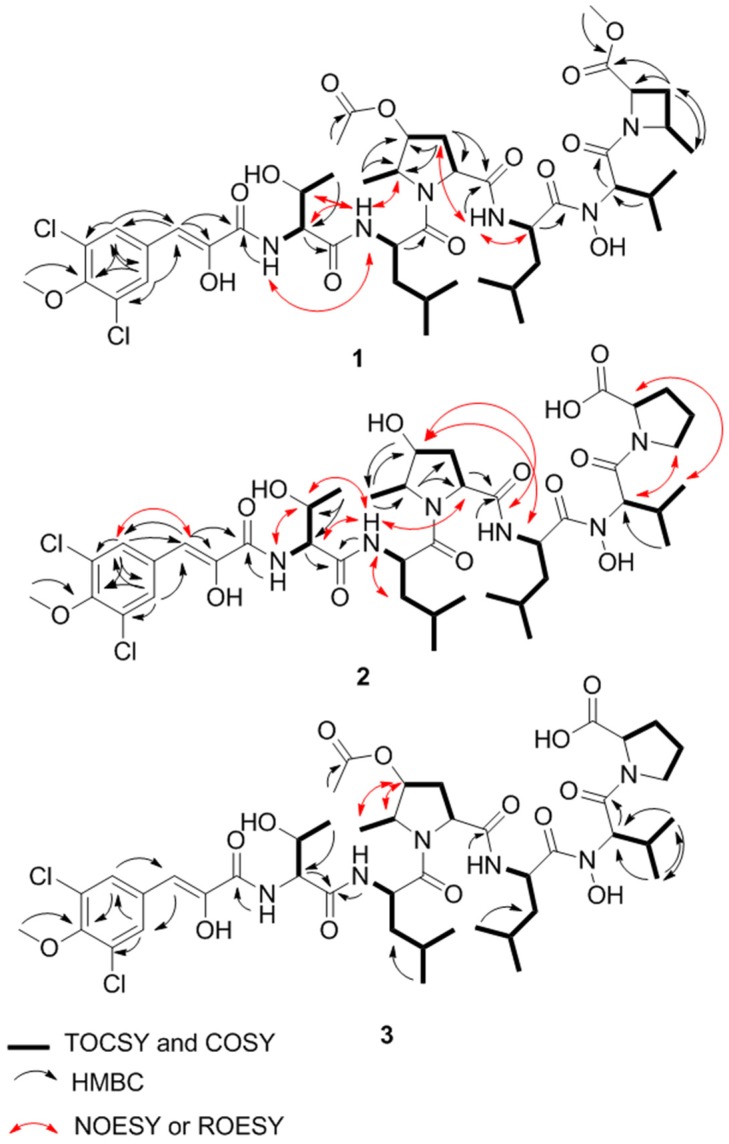
The 2D NMR correlations of compounds **1**–**3**.

**Figure 3:**
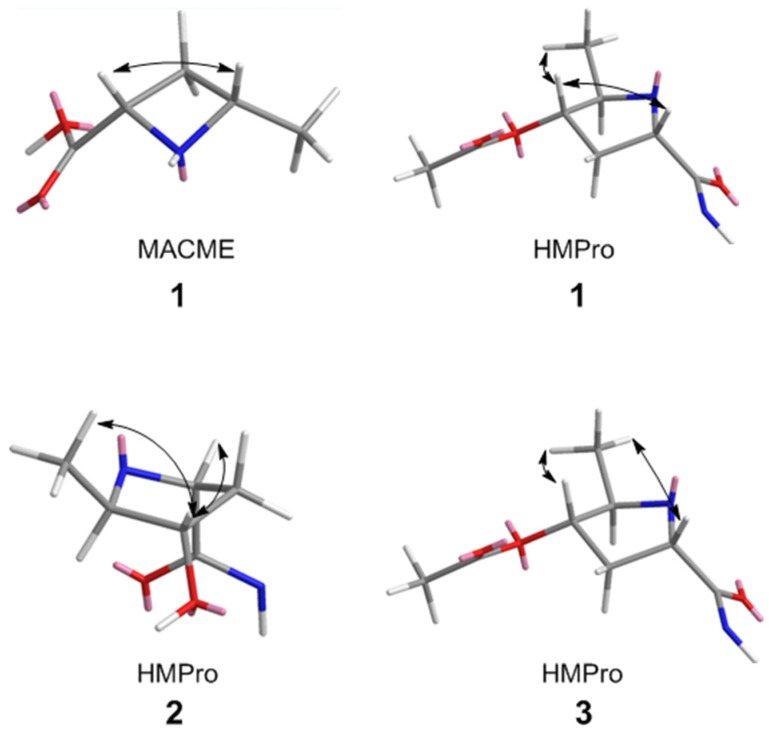
Stick models of 4-methyl-azetidine-2-carboxylic acid methyl ester (MACME) and 4-hydroxy-5-methylproline (HMPro) residues in **1**–**3**. Key 1D NOE correlations are shown as black arrows. Atom definitions: red, oxygen; blue, nitrogen; gray, carbon; white, hydrogen.

Compound **1** was isolated as a light red solid. The isotopic ratio of **1** indicated the presence of two chlorine atoms and its molecular formula was determined as C_45_H_66_Cl_2_N_6_O_14_ using positive high-resolution electrospray ionization mass spectroscopy (HRESIMS) (*m/z* 985.4048 [M + H]^+^, calcd. for 985.4092). The peptide nature of **1** was suggested by the signal distribution observed in the ^1^H and ^13^C NMR spectra, which included exchangeable amide NH signals, amino acid α proton signals and seven carbonyl carbons. Careful interpretation of ^1^H-^1^H Correlation Spectroscopy (COSY), ^1^H-^1^H Total Correlation Spectroscopy (TOCSY) and gradient ^1^H-^13^C Heteronuclear Single Quantum Coherence (gHSQCAD) spectra allowed us to establish spin systems ascribable to threonine (Thr), valine (Val) and two leucine (Leu) residues (Figure 2). Unlike the other amino acids, the exchangeable amide proton in Val was not observed in the ^1^H NMR spectrum (Table 1). This signal was also not detected in the 2D Heteronuclear Multiple Bond Correlation (HMBC) and COSY correlations, suggesting the presence of an *N*, *N*-disubstituted amide. LC-MS/MS indicated a hydroxyl group was incorporated in place of the amide proton yielding a hydroxamic acid moiety. The highly modified 4-hydroxy-5-methylproline (HMPro) residue was identified as a new non-proteinogenic amino acid and its structure was deduced from TOCSY and HMBC experiments. MS/MS fragmentation and formula weight analysis showed that the C-4 hydroxyl group of HMPro in compound **1** is acetylated. Correlations from H_3_-6_HMPro_ to C-5_HMPro_ and C-4_HMPro_ and from H_2_-3_HMPro_ to C-4_HMPro_ in the HMBC spectrum combined with the H-2_HMPro_/H_2_-3_HMPro_/H-4_HMPro_ and H-5_HMPro_/H_3_-6_HMPro_TOCSY correlations indicated that the methyl group is on C-5 and the acetyl group is located on C-4 (Figure 2). Irradiating *δ*_H_ 4.88 produced an NOE enhancement for H_3_-6_HMPro_ and H-2_HMPro_ indicating the co-facial positioning of the protons (Figure 3). Considering the final spin system of H-2_MACME_/H_2_-3_MACME_/H-4_MACME_/H_3_-5_MACME_ and the nitrogen-containing nature of C-2_MACME_ and C-4_MACME_ (Figure 2), a 4-methyl-azetidine-2-carboxylic acid methyl ester residue was proposed. HMBC correlations from *δ*_H_ 3.67, *δ*_H_ 2.69 and *δ*_H_ 1.72 to the carbonyl carbon at *δ*_C_ 171.6 suggested the presence of a methyl ester appended onto C-2_MACME_, whereas correlations from *δ*_H_ 1.47 to the methylene *δ*_C_ 27.9 placed the methyl group on C-4_MACME_ (Figure 2). A 1D Nuclear Overhauser Effect Spectroscopy (NOESY) was used to assign the relative configuration of MACME (Figure 3). Additionally, detailed analysis of the remaining signals, which were distributed to a symmetrical substituted benzene ring (*δ*_C_ 127.0, C-4_BVA_; *δ*_C_ 129.8, C-5 and C-5′_BVA_; *δ*_C_ 122.7, C-6 and C-6′_BVA_; *δ*_C_ 149.4, C-7_BVA_; *δ*_H_ 7.73, H-5 and H-5′_BVA_), an enolic double bond (*δ*_C_ 149.5, C-2_BVA_; *δ*_C_ 115.9, C-3_BVA_; *δ*_H_ 6.66, H-3_BVA_), a carbonyl carbon (*δ*_C_ 163.1, C-1_BVA_), and a methoxy group (*δ*_C_ 60.1, C-8_BVA_; *δ*_H_3.67, H-8_BVA_) was used to characterize the bonnevillic acid (BVA) (3-(3,5-dichloro-4-methoxyphenyl)-2-hydroxyacrylic acid) building block. The BVA moiety was further supported by extensive HMBC correlations (Figure 2) and by comparing chemical shifts with those found in the aeruginosins [11,12].

**Figure 4:**
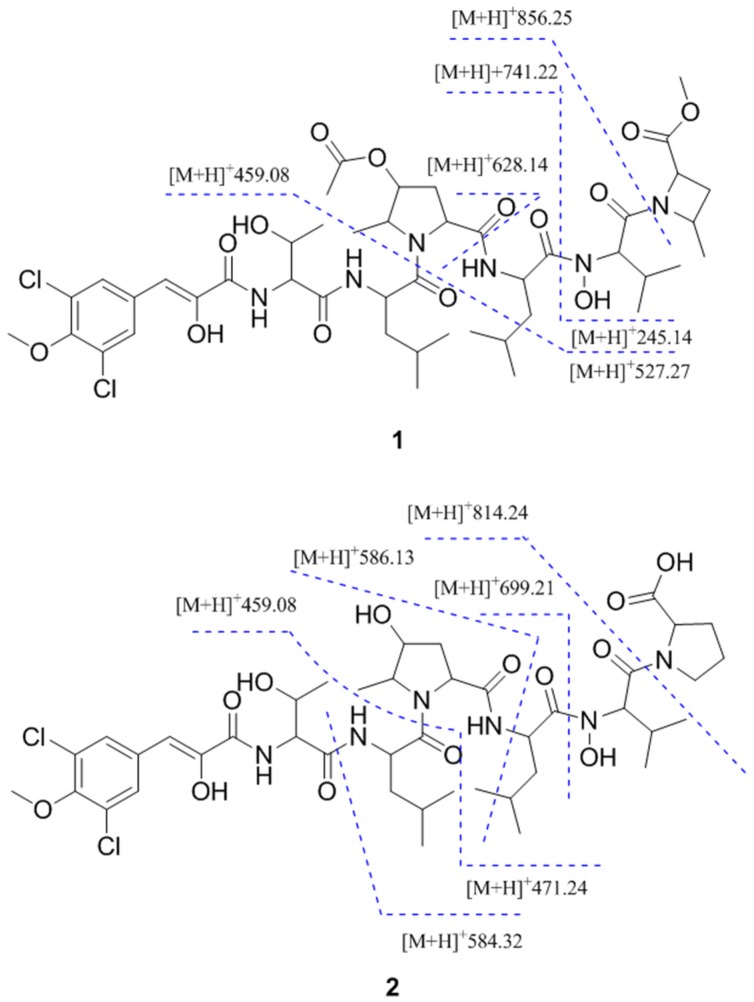
LC–MS/MS data for **1** and **2**.

Assignment of the amino acid sequence was carried out via a combination of HMBC, NOESY, Rotating Frame Nuclear Overhauser Effect Spectroscopy (ROESY) and MS/MS analysis. HMBC correlations from NH_Thr_ (*δ*_H_ 7.67) to C-1_BVA_ and from NH_Leu2_ (*δ*_H_ 8.25) to C-1_HMPro_ sequentially connected BVA to Thr and HMPro to Leu2 (Figure 2). NOESY correlations between NH_Thr_ and NH_Leu1_ and between NH_Leu1_ and H-5_HMPro_ elongated the sequence to BVA-Thr-Leu-HMPro-Leu2 (Figure 2). Due to the presence of the methyl ester in the MACME residue, MACME was determined to be the terminal amino acid residue. Thus, the remaining Val residue must reside between Leu2 and MACME. The order of amino acids was further confirmed with a detailed analysis of LC–MS/MS data of **1** (Figure 4). This LC–MS/MS data of **1** provided further evidence for the presence of a hydroxamic acid moiety on Val and an acetylated HMPro residue. Consequently, the planar structure of **1** was elucidated as a linear heptapeptide with the sequence BVA-Thr-Leu1-O-Ac-HMPro-Leu2-N-OH-Val-MACME (Figure 1).

Compounds **2** and **3** were isolated as light red solids. Their molecular formulas were calculated as C_42_H_62_Cl_2_N_6_O_13_ and C_44_H_64_Cl_2_N_6_O_14_ by HRESIMS (*m/z* 929.3805 [M + H]^+^, calcd. for 929.3830; *m/z* 971.3926 [M + H]^+^, calcd. for 971.3936), respectively. Comparing the UV and ^1^H NMR spectra of **2** and **3** with those of **1** indicated that the compounds were analogs of **1**. For compound **2**, the MACME residue is replaced by Pro and the acetyl group observed on HMPro is absent. The substitution of Pro and loss of the acetyl moiety were supported by 1D and 2D NMR correlations (Figure 1 and Figure 2). The sequence of amino acid residues of **2** was assigned by HMBC, NOESY, ROESY and LC–MS/MS analysis (Figure 4). Comparing 1D and 2D NMR spectra between compounds **2** and **3** revealed that compound **3** was an OH-4_HMPro_ acetylated derivative of **2**. The 1D NOESY experiments confirmed that the HMPro residue in compounds **2** and **3** had the same relative configuration as that in compound **1** (Figure 3). Ultimately, the order of amino residues in compounds **2** and **3** were assigned as BVA-Thr-Leu1-HMPro-Leu2-N-OH-Val-Pro and BVA-Thr-Leu1-O-Ac-HMPro-Leu2-N-OH-Val-Pro, respectively.

To determine the absolute configurations of the proteinogenic amino acid residues in compounds **1**–**3**, an advanced Marfey’s method was performed [13,14,15]. Each hydrolysate of **1**–**3** was divided into two fractions and derivatized with either 1-fluoro-2,4-dinitrophenyl-5-d,l-leucinamide (d,l-FDLA) or l-FDLA. The d,l-FDLA- or l-FDLA-derived products were monitored by LC-MS. For compounds **1**–**3**, l-Thr, l-Leu, l-Val and l-Pro were deduced through retention time comparisons (Table S1). As no commercial standards are available for HMPro and MACME, their absolute configurations have not yet been determined.

### 2.2. Biosynthetic Proposal

The genome of *Streptomyces* sp. GSL-6B is currently being sequenced and when it is completed, the sequenced genome will be mined for the bonnevillamide biosynthetic cluster. Based on the linear arrangement of proteinogenic and non-proteinogenic amino acids, we propose that the bonnevillamides are assembled via a heptamodular non-ribosomal peptide synthetase (NRPS). NRPSs act as biosynthetic templates and typically assemble nonribosomal peptides in an assembly-line fashion. The catalytic domains embedded within the NRPS dictate the types of building blocks that are to be used for the assembly of a compound, as well as some modifications to the proteinogenic or non-proteinogenic substrates. Specialized monomers or building blocks required for the assembly of a natural product, such as non-proteinogenic amino acids, are generally synthesized from primary metabolites using a dedicated collection of genes located within the corresponding gene cluster [16]. The biosynthesis of compounds **1**–**3** is initiated with the unique BVA starter unit, which is most likely derived from tryosine. If this is the case, a tryosine ammonia lyase should be located in close proximity to the NRPS in which it catalyzes the elimination of ammonia to yield p-coumaric acid [17]. Additional modifications such as oxidation, halogenation and methylation would be required to produce the final BVA residue. The next NRPS module would incorporate a threonine residue, whereas modules 3, 4, 5 and 6 would incorporate leucine, proline, leucine and valine, respectively. Further inspection of module 6, as well as post-NRPS tailoring enzymes, will provide insight into the formation of the hydroxamic acid moiety on valine. Based on the structural similarities of the three bonnevillamides, we hypothesize that the compounds are biosynthesized by the same NRPS and that module 7 incorporates Pro. To form **1**, post-NRPS tailoring enzymes may convert the terminal Pro residue to MACME and feeding studies with stable isotope-labeled amino acids will help elucidate the biosynthetic origin of this rare residue.

### 2.3. Bioactivity

**Figure 5:**
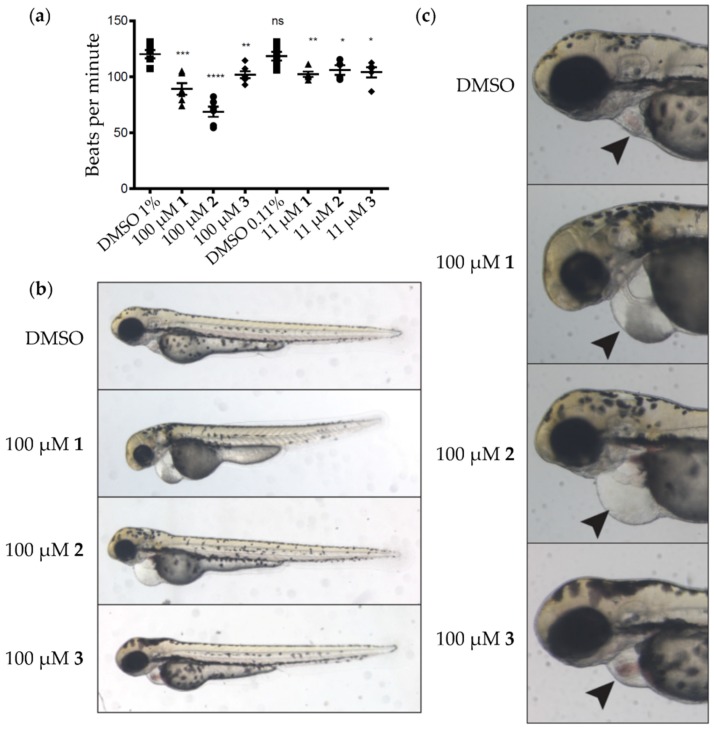
Evaluating the activity of compounds **1**–**3** using a zebrafish embryonic development assay: (**a**) Quantification of 60 hpf zebrafish embryo heartrate. Each square, triangle, circle or diamond represents a single fish and shape definitions are: DMSO, square; **1**, triangle; **2**, circle; and **3**, diamond. Statistics from Student’s *t*-test (compared to 1% DMSO-treated samples): * *p* < 0.05, ** *p* < 0.01, *** *p* < 0.001 and **** *p* < 0.0001; (**b**) Representative images of 75 hpf zebrafish treated with the indicated bonnevillamides; (**c**) Magnified view of panel (**b**) highlighting the pericardial edema and abnormal heart morphology.

Using the broth dilution method, compounds **1**–**3** were initially screened for antimicrobial activity. Unfortunately, no significant effect on the growth of *Staphylococcus aureus* (ATCC 126,000), *Escherichia coli* (D22 and ATCC 25,922), *Pseudomonas aeruginosa* (ATCC 27,853), *Klebsiella pneumoniae* (ATCC 13883), or *Acinetobacter baummannii* (ATCC BAA-1605) was observed for **1**‒**3** at 32 µg/mL. The bonnevillamides were then evaluated for activity using a zebrafish embryonic development assay. Phenotypic changes were monitored using various concentrations of compound and at 100 μM, all three compounds had a pronounced effect on heart development and cardiac function compared to a DMSO control (Figure 5). A decreased heart rate, an enlarged pericardial sac and abnormal heart morphology were observed. Compound **2** was the most active, suggesting that the free hydroxyl found on HMPro may be responsible for its increased activity compared to **1** and **3**. Additional studies are underway to identify the molecular target(s) of the bonnevillamides.

## 3. Materials and Methods

### 3.1. General Experimental Procedures

Optical rotations were measured on a Jasco DIP-370 polarimeter (Oklahoma City, OK, USA), UV spectra were recorded on a Perkin-Elmer Lamdba2 (Waltham, MA, USA) UV/vis spectrometer and NMR spectra were recorded on Varian INOVA (Palo Alto, CA, USA) 500 and 600 NMR spectrometers with 3 mm Nalorac MDBG probes. High resolution ESIMS was carried out on a Bruker (Billerica, MA, USA) maxis II ETD Q-TOF instrument and HPLC-MS/MS was recorded using a Micromass Q-TOF Micro Mass Spectrometer (Waters, Milford, MA, USA) in positive mode with an Agilent Eclipse XDB-C18 column (150 × 4.6 mm, 5 μm). Semi-preparative HPLC was performed using an ODS column (Thermo Scientific, Waltham, MA, USA, HYPERSIL GOLD 10 × 250 mm, 12 μm, 3 mL/min). Column chromatography was performed with Sephadex LH-20 (GE Healthcare Bio-Sciences AB, Uppsala, Sweden).

### 3.2. Isolation and Fermentation of Streptomyces sp. GSL-6B

*Streptomyces* sp. GSL-6B was isolated from sediment collected from Farmington Bay in the Great Salt Lake, UT, USA. Sediment samples were collected at a depth of 0.6 m and placed in sterile 50 mL conical tubes and kept cool until processed. In a biological safety cabinet, 20 mL of wet sediment was dried for 48 h. Approximately 0.5 g of dried sediment was added to yeast-peptone-mannitol (YPM) agar (4 g/L mannitol, 2 g/L yeast extract, 2 g/L peptone, and 42 g/L Instant Ocean Aquarium Sea Salt Mixture, Spectrum Brands, USA) and incubated at 30 °C for up to 90 days. Bacterial colonies were subcultured on YPM until pure isolates were obtained. 16S sequencing was used to identify GSL-6B as a *Streptomyces* sp. (GenBank accession number for the partial 16S rDNA sequence is MF288649). *Streptomyces* sp. GSL-6B was cultured at 28 °C for four days with constant shaking at 160 rpm in 125 mL Erlenmeyer flasks containing 50 mL of marine-based fermentation media (glucose (20.0 g/L), beef extract (3.0 g/L), yeast extract (10.0 g/L), soluble starch (10.0 g/L), K_2_HPO_4_ (0.5 g/L), MgSO_4_ (0.5 g/L), CaCO_3_ (2.0 g/L), and artificial sea salt (33.0 g/L, Instant Ocean Aquarium Sea Salt Mixture) at pH = 7.0). After five days, 20 2.8 L Fernbach flasks containing 1 L of marine-based fermentation media were inoculated using 1% of the seed cultures and incubated at 28 °C for nine days with constant shaking at 160 rpm.

### 3.3. Extraction and Isolation 

After 9 days of fermentation, 20 L of broth was centrifuged and the supernatant was incubated with HP-20 resin (20 g/L) with shaking at 120 rpm for 2 h. The resin was then filtered through miracloth, washed three times with water (200 mL aliquots) to remove salts and extracted three times with methanol (300 mL aliquots) to yield the supernatant crude extract. The mycelium was extracted three times with 70% acetone (500 mL total volume) and concentrated *in vacuo*. To the ~1 mL of oil, 300 mL of water was added and partitioned with an equal volume of ethyl acetate to yield the mycelium crude extract. By LC–MS, it was observed that the bonnevillamides were found in both the supernatant and mycelium. The crude extracts were combined, concentrated *in vacuo* and fractionated with a SephadexLH-20 column (60 cm (length) × 1.5 cm (diameter)) using 50 mL aliquots of 100% MeOH. Based on mass spectroscopy and UV, fraction 1 contained the targeted compounds and was subjected to further purification via semi-preparative HPLC. A Hypersil Gold 10 × 250 mm C18 column was used at a flow rate of 3 mL/min and an isocratic condition of 65% MeOH:H_2_O with 0.1% TFA to afford compounds **1** (5.0 mg, *t*_R_ = 16.1 min), **2** (5.1 mg, *t*_R_ = 16.8 min), and **3** (3.0 mg, *t*_R_ = 17.2 min).

Bonnevillamide A (**1**): light red solid; [α]${}_{D}^{20}$ −30° (*c* 0.1, CHCl_3_); UV (MeOH) λ_max_ (log ε) 236 (3.00), 295 (3.96) nm; ^1^H and ^13^C NMR data, Table 1 and Supporting Information; HRESIMS *m/z* 985.4048 [M + H]^+^ (calcd. for C_45_H_67_Cl_2_N_6_O_14_, 985.4092).

Bonnevillamide B (**2**): light red solid; [α]${}_{D}^{20}$ −25° (*c* 0.1, CHCl_3_); UV (MeOH) λ_max_ (log ε) 236 (3.05), 294 (4.06) nm; ^1^H and ^13^C NMR data, Table 1 and Supporting Information; HRESIMS *m/z* 929.3805 [M + H]^+^ (calcd. for C_42_H_63_Cl_2_N_6_O_13_, 929.3830).

Bonnevillamide C (**3**): light red solid; [α]${}_{D}^{20}$ −40° (*c* 0.1, CHCl_3_); UV (MeOH) λ_max_ (log ε) 235 (3.07), 295 (4.09) nm; ^1^H and ^13^C NMR data, Table 1 and Supporting Information; HRESIMS *m/z* 971.3926 [M + H]^+^ (calcd. for C_44_H_65_Cl_2_N_6_O_14_, 971.3936).

### 3.4. Advanced Marfey’s Anslysis

Each peptide (~0.2 mg) was dissolved in 500 µL 6 N·HCl and heated in an ampule for 17 h at 110 °C. The hydrolysate was evaporated to dryness *in vacuo* and re-dissolved in 100 μL H_2_O. The solution was divided into two fractions and to each fraction, 20 μL 1 N·NaHCO_3_ and 100 μL of either d,l-FDLA or l-FDLA (1% solution in acetone) was added. The mixture was heated for 50 min at 40 °C, quenched by adding 20 μL 1N·HCl and dried *in vacuo*. The residue was dissolved in 1:1 CH_3_CN-H_2_O and analyzed by LC–MS. Separation of the residues was carried out using an Agilent (Santa Clara, CA, USA) Eclipse XDB-C18 column (5 μm, 4.6 × 150 mm) at a flow rate of 0.5 mL/min with a linear gradient of 5% to 100% CH_3_CN in 0.1% formic acid over 45 min. Absolute configurations of the proteinogenic amino acids were determined by comparing the retention times of the d,l- and l-FDLA derivatives, which were identified by MS. Retention times of the d,l-FDLA amino acid derivatives were 22.03 and 24.57 min (Thr), 27.94 and 32.03 min (Leu), 26.69 and 29.95 min (Val), and 24.41 and 26.00 min (Pro) (Figures S33‒S35 and Table S1). The retention times of the l-FDLA amino acid derivatives were 22.08 min (Thr), 28.03 min (Leu), 26.69 min (Val), and 24.35 min (Pro) (Figures S33‒S35 and Table S1).

### 3.5. Zebrafish Assays

Animals were maintained and embryos were obtained according to standard fish husbandry protocols. Fertilized eggs were collected from group mating of TuAB zebrafish and stored in E3 media at 28 °C until 2 hpf. At 2 hpf, groups of ~5 embryos were distributed into the wells of flat-bottom 96 well plates filled with E3 media (400 μL) and DMSO, **1**, **2**, or **3** were added at the indicated concentrations. The plates of embryos were then incubated at 28 °C. All zebrafish protocols were approved by the Institutional Animal Care and Use Committee at the University of Utah.

### 3.6. Heartrate Analysis

The amount of time required for the heart of a 60 hpf zebrafish embryo to beat 20 times was measured manually, by monitoring the heart of each embryo under a Zeiss SteREO Discovery V8 microscope (Zeiss, Jena, Germany), and used to extrapolate the number of heart beats per minute for a single zebrafish embryo.

## 4. Conclusions

Biological pressures can influence the chemical diversity of secondary metabolites and it has been shown that microorganisms isolated from extreme environments often produce molecules not observed in their terrestrial counterparts. A chemical investigation of *Streptomyces* sp. GSL-6B, isolated from sediment collected from the Great Salt Lake, led to the isolation of bonnevillamides A–C (**1**–**3**). The bonnevillamides represent a new class of linear heptapetides featuring novel amino acid residues. All three compounds contain the newly characterized bonnevillic acid building block, as well as a heavily modified proline residue. Additionally, while bonnevillamide B (**2**) and C (**3**) contain a terminal proline residue, bonnevillamide A (**1**) contains an extremely rare 4-methyl-azetidine-2-carboxylic acid methyl ester moiety. The three peptides were evaluated for their effects on zebrafish embryo development and shown to modulate heart growth and cardiac function, with bonnevillamide B (**2**) having the most pronounced effect. Preliminary structure–activity relationship data suggests that the free hydroxyl found on the 4-hydroxy-5-methylproline residue in **2** appears to be a key structural feature for the observed activity. All three structures were characterized by 1D and 2D NMR spectroscopy and absolute configuration of the proteinogenic amino acid residues was determined using the advanced Marfey’s method.

## Figures and Tables

**Table 1:** NMR data of **1**–**3** in DMSO-*d*_6_.

Residue	Position	Bonnevillamide A (1)		Bonnevillamide B (2)		Bonnevillamide C (3)	
		*δ*_C_^a^	*δ*_H_ (*J* in Hz) ^b^	*δ*_C_^a^	*δ*_H_ (*J* in Hz) ^b^	*δ*_C_^c^	*δ*_H_ (*J* in Hz) ^b^
BVA	1	163.1 C	-	163.1 C	-	163.1 C	-
	2	149.5 C	-	149.5 C	-	149.5 C	-
	3	115.9 CH	6.66, s	116.1 CH	6.66, s	115.9 CH	6.67, s
	4	127.0 C	-	126.9 C	-	^d^	-
	5/5′	129.8 CH	7.73, s	129.8 CH	7.70, s	129.7 CH	7.73, s
	6/6′	122.7 C	-	122.7 C	-	122.6 C	-
	7	149.4 C	-	149.5 C	-	149.5 C	-
	8	60.1 CH_3_	3.67, s	59.5 CH_3_	3.65, s	59.4 CH_3_	3.67, s
Thr	1	170.2 C	-	169.8 C	-	170.2 C	-
	2	59.1 CH	4.26, dd (4.8, 8.6)	58.8 CH	4.30, m	59.1 CH	4.27, dd (5.0, 8.6)
	3	67.2 CH	3.93, m	67.5 CH	3.97, m	67.1 CH	3.97, m
	4	20.8 CH_3_	1.05, d (6.4)	20.1 CH_3_	1.02, d (5.2)	20.7 CH_3_	1.05, d (6.3)
	NH	-	7.67, d (8.6)	-	7.65, d (7.4)	-	7.68, d (8.5)
Leu 1	1	173.8 C	-	173.7 C	-	^d^	-
	2	48.0 CH	4.98, m	47.9 CH	4.95, m	48.6 CH	4.49, m
	3	40.1 CH_2_	1.37, m; 1.52, m	40.5 CH_2_	1.34, m; 1.46, m	41.4 CH_2_	1.31, m; 1.62, m
	4	24.4 CH	1.66, m	24.7 CH	1.64, m	24.7 CH	1.62, m
	5	22.1 CH_3_	0.83, overlap	23.6 CH_3_	0.85, overlap	21.5 CH_3_	0.82, overlap
	6	22.4 CH_3_	0.83, overlap	22.2 CH_3_	0.83, overlap	23.7 CH_3_	0.89, d (6.9)
	NH	-	7.83, d (8.4)		7.76, d (6.8)	-	8.26, d (8.0)
HMPro	1	170.7 C	-	171.3 C	-	170.7 C	-
	2	58.4 CH	4.43, overlap	58.8 CH	4.44, m	58.4 CH	4.51, overlap
	3	32.1 CH_2_	2.15, m	35.2 CH_2_	1.92, m; 1.98, m	32.2 CH_2_	2.16, m; 2.22 m
	4	78.5 CH	4.88, m	75.3 CH	3.90, brs	78.5 CH	4.89, d
	5	60.1 CH	4.49, m	62.4 CH	4.01, m	60.1 CH	4.49, overlap
	6	19.1 CH_3_	1.18, d (6.8)	19.3 CH_3_	1.10, d (6.7)	19.1 CH_3_	1.18, d (6.9)
	7	170.5 C	-	-	-	170.5 C	-
	8	21.3 CH_3_	1.99, s	-	-	21.4 CH_3_	2.00, s
Leu 2	1	171.2 C	-	167.3 C	-	^d^	-
	2	48.6 CH	4.49, m	48.3 CH	4.66, m	48.1 CH	4.97, m
	3	41.3 CH_2_	1.31, m; 1.62, m	41.9 CH_2_	1.38, m; 1.47, m	40.3 CH_2_	1.37, m; 1.50, m
	4	24.7 CH	1.62, m	24.5 CH	1.57, m	24.5 CH	1.67, m
	5	21.5 CH_3_	0.82, overlap	23.8 CH_3_	0.84, overlap	22.1 CH_3_	0.83, overlap
	6	23.7 CH_3_	0.88, d (6.9)	21.5 CH_3_	0.80, overlap	22.3 CH_3_	0.83, overlap
	NH	-	8.25, d (8.0)	-	8.20, d (7.6)	-	7.82, d (8.6)
N-OH-Val	1	168.3 C	-	167.3 C	-	167.3 C	-
	2	61.2 CH	4.43, m	62.4 CH	4.66, m	62.4 CH	4.70, d (10.5)
	3	26.1 CH_2_	2.31, m	26.8 CH	2.30, m	26.9 CH_2_	2.34, m
	4	18.9 CH_3_	0.77, d (6.7)	19.0 CH_3_	0.76, d (7.0)	19.0 CH_3_	0.79, d (6.7)
	5	19.7 CH_3_	0.84, overlap	19.5 CH_3_	0.83, overlap	19.5 CH_3_	0.88, d (6.6)
MACME/Pro	1	171.6 C	-	171.1 C	-	^d^	-
	2	56.2 CH	4.49, m	59.1 CH	4.18, m	59.2 CH	4.21, dd (4.6, 8.7)
	3	27.9 CH_2_	2.69, m; 1.72, m	29.2 CH_2_	1.78, m; 2.10, m	29.2 CH_2_	1.83, m; 2.14, m
	4	57.3 CH	4.36, m	25.0 CH_2_	1.80, m; 1.90, m	24.9 CH_2_	1.91, m; 1.83, m
	5	22.4 CH_3_	1.47, d (6.2)	46.9 CH_2_	3.47, m	46.9 CH_2_	3.50, m
	6	52.4 CH_3_	3.67, s	-	-	-	-

^a^, ^13^C spectrum was recorded in DMSO-*d*_6_ in 500 MHz NMR; ^b^, ^1^H spectrum was recorded in DMSO-*d*_6_ in 600 MHz NMR; ^c^, The carbon chemical shifts were deduced by HSQC and HMBC experiments; ^d^, Carbon signals were not detected.

## Supplementary Materials

The following are available online at www.mdpi.com/1660-3397/15/7/195/s1, Figure S1: UV spectra of compounds **1**–**3**; Figures S2–S11: ^1^H NMR,^13^C NMR, gHSQCAD , gHMBCAD, TOCSY, COSY, ROESY, NOESY and 1D NOE of bonnevillamide A (**1**) in DMSO-*d6*; Figures S12–S13: HRESIMS/MS and HR(+)ESIMS of bonnevillamide A (**1**); Figures S14–S22: ^1^H NMR,^13^C NMR, gHSQCAD, gHMBCAD, TOCSY, COSY, ROESY, NOESY and 1D NOE of bonnevillamide B (**2**) in DMSO-*d6*; Figures S23–S24: HRESIMS/MS and HR(+)ESIMS of bonnevillamide B (**2**); Figures S25–S31: ^1^H NMR, gHSQCAD, gHSQCAD, TOCSY, COSY, ROESY, and NOESY spectrum of bonnevillamide C (**3**) in DMSO-*d6*; Figure S32: HR(+)ESIMS of bonnevillamide C (**3**); Figure S33: Advanced Marfey’s analysis of bonnevillamide A (**1**); Figure S34: Advanced Marfey’s analysis of bonnevillamide B (**2**); Figure S35: Advanced Marfey’s analysis of bonnevillamide C (**3**); Table S1: Retention times of amino acid derivatives used in advanced Marfey’s analysis.
