# Supplementary material for: Bonnevillamides, Linear Heptapeptides Isolated from a Great Salt Lake-Derived Streptomyces sp
Source: Mar Drugs. 2017 Jun 24;15(7):195. doi: 10.3390/md15070195 (PMC5532637; doi:10.3390/md15070195)
Supplement: Supplementary file 1 [file marinedrugs-15-00195-s001.pdf]

Supporting Information

Bonnevillamides, Linear Heptapeptides Isolated from a Great Salt Lake-Derived Streptomyces sp.

Guangwei Wu [1], Jason R. Nielson [2], Randall T. Peterson [2], and Jaclyn M. Winter [1,*]

[1]Department of Medicinal Chemistry, University of Utah, Salt Lake City, UT 84112, USA;Guangwei.Wu@utah.edu

[2]Department of Pharmacology and Toxicology, University of Utah, Salt Lake City, UT 84112, USA; Jason.Nielson@pharm.utah.edu (J. R. N); Randall.Peterson@pharm.utah.edu (R. T. P.)

*****Correspondence: Jaclyn.Winter@utah.edu; Tel.: +1-801-585-7117

List of Supporting Information

- **Figure S1.** UV spectra of compounds **1**–**3**...S3
- **Figure S2-S5.** ^{1}H, ^{13}C, gHSQCAD, gHMBCAD spectra of compound **1**...............................S4-S7
- **Figure S6-S7.** TOCSY and COSY spectra of compound **1**...S8-S9
- **Figure S8-S11.** ROESY, NOESY and 1D NOE spectra of compound **1**............................S10-S13
- **Figure S12-S13.** LC-MS/MS and HR(+)ESIMS spectra of compound **1**..........................S14-S15
- **Figure S14-S17.** ^{1}H, ^{13}C, gHSQCAD, gHMBCAD spectra of compound **2**....................... S16-S19
- **Figure S18-S19.** TOCSY and COSY spectra of compound **2**..S20-S21
- **Figure S20-S22.** ROESY, NOESY and1D NOE spectra of compound **2**............................S22-S24
- **Figure S23-S24.** LC-MS/MS and HR(+)ESIMS spectra of compound **2**............................S25-S26
- **Figure S25-S27.** ^{1}H, gHSQCAD, gHMBCAD spectra of compound **3**.............................S27-S29
- **Figure S28-S29.** TOCSY and COSY spectra of compound **3**..S30-S31
- **Figure S30-S31.** ROESY, NOESY spectra of compound **3**...S32-S33
- **Figure S32.** HR(+)ESIMS spectra of compound **3**..S34
- **Figure S33.** Advanced Marfey's analysis of acid hydrolysate of **1**............................S35-S38
- **Figure S34.** Advanced Marfey's analysis of acid hydrolysate of **2**............................S39-S44
- **Figure S35.** Advanced Marfey's analysis of acid hydrolysate of **3**............................S45-S49
- **Table S1.** Corresponding retention times between D,L-FDLA derivatives of amino acids..........S50

Figure S1. UV spectra of compounds **1–3**.

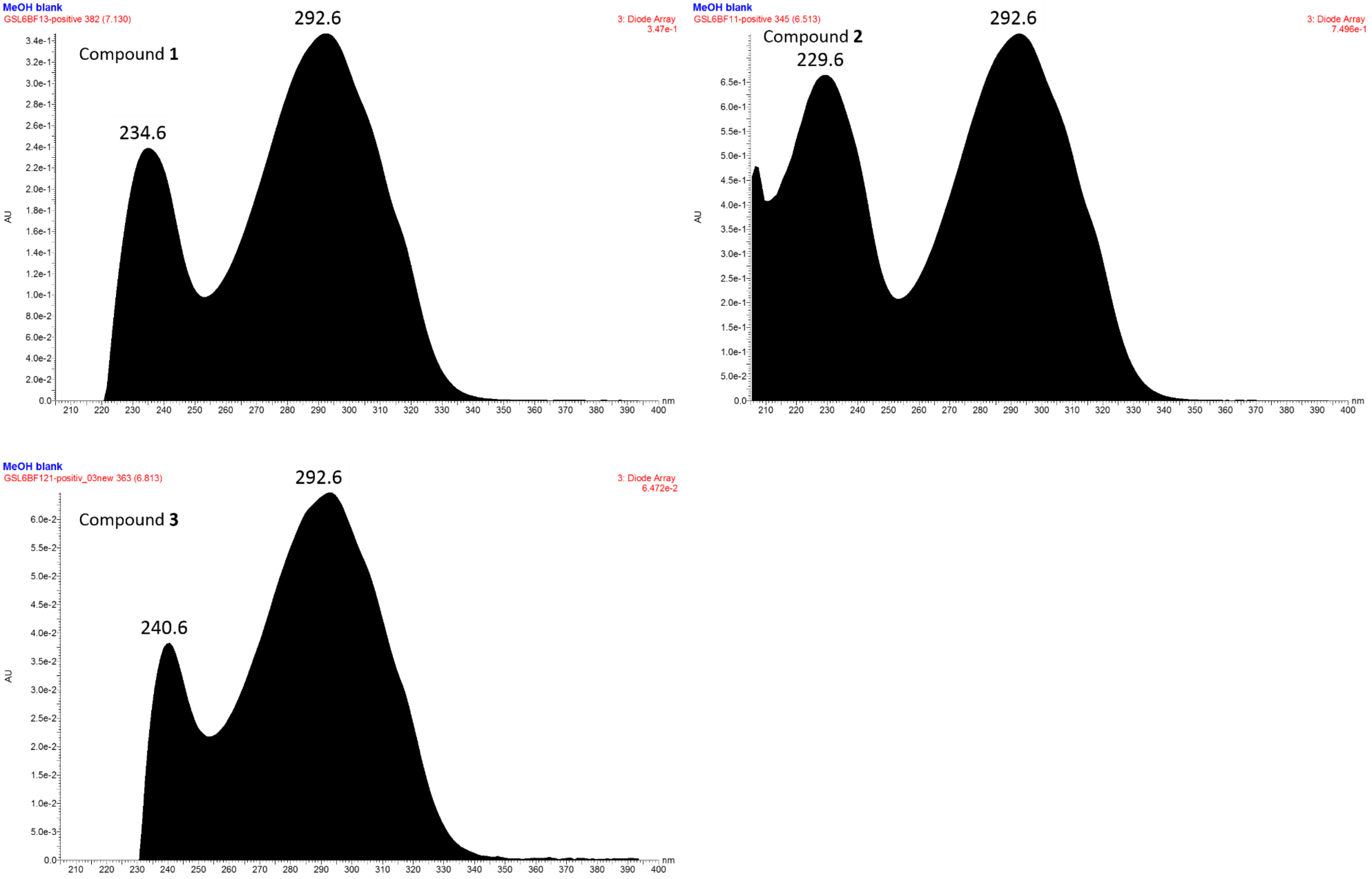

Figure S2. ^{1}H NMR spectrum of compound **1** in DMSO-d_6

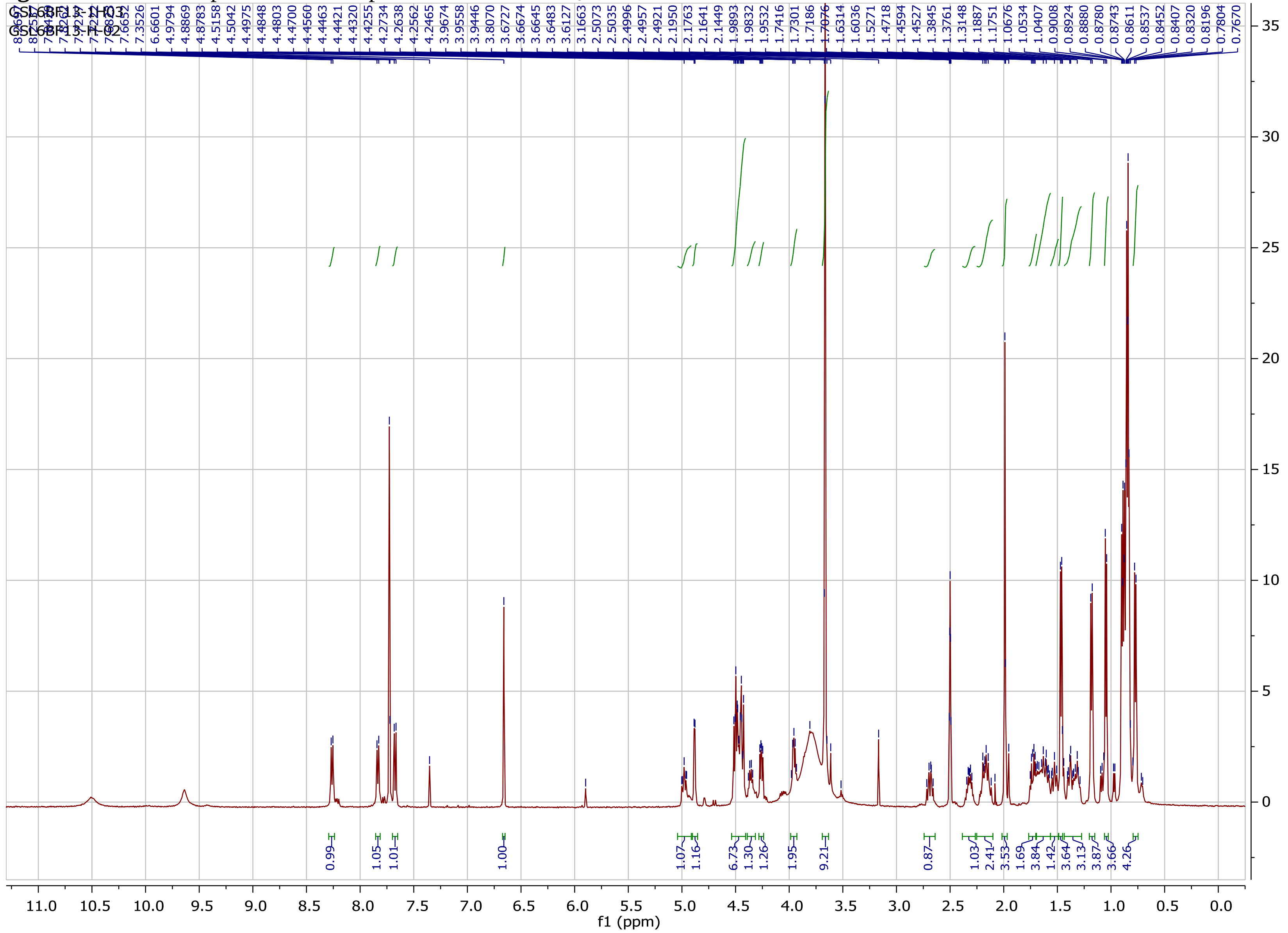

Figure S3. ^{13}C NMR spectrum of compound **1** in DMSO-d_6

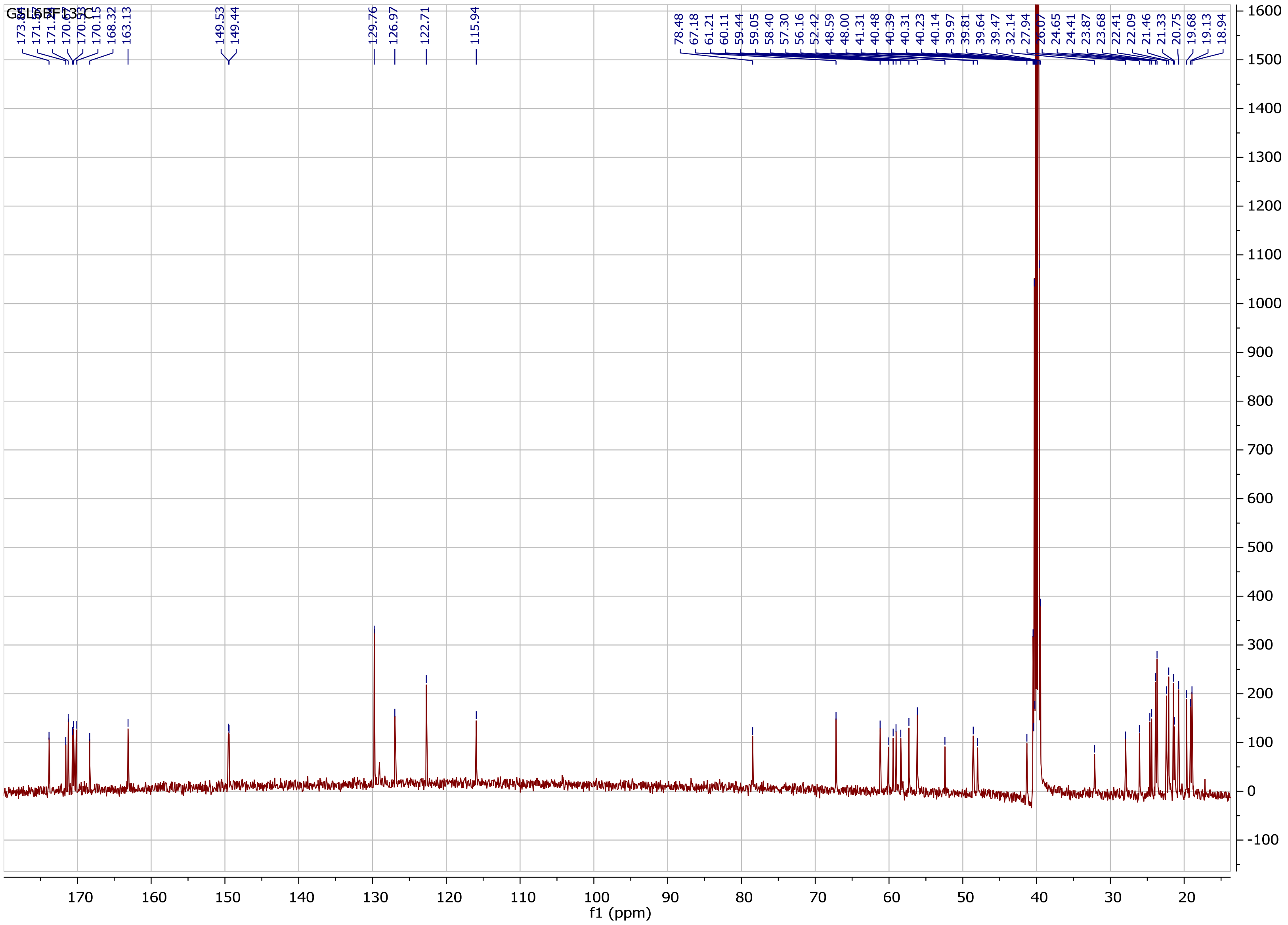

63 **Figure S4**.gHSQCAD spectrum of compound **1** in DMSO-d_6

64

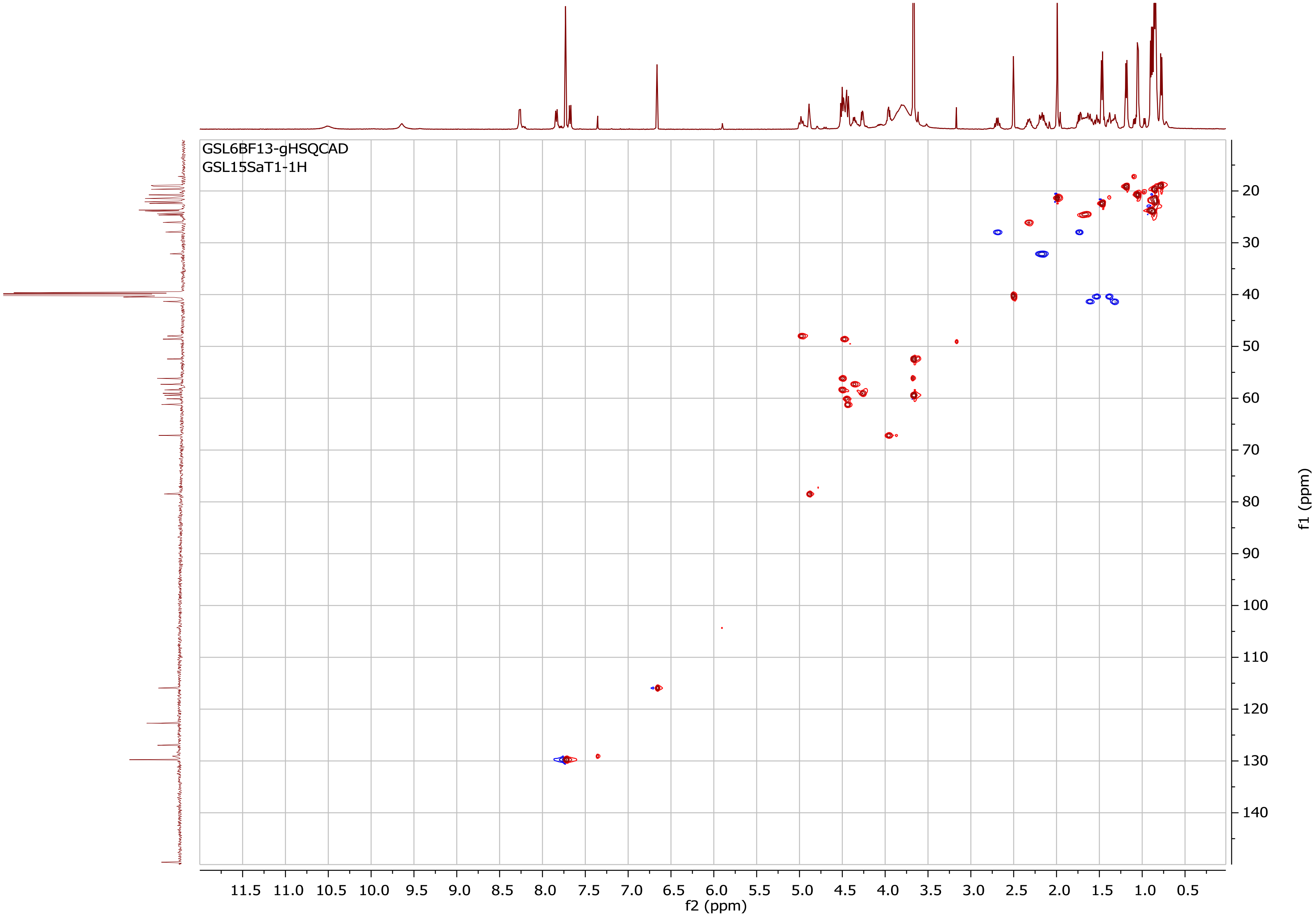

65

Figure S5.gHMBCAD spectrum of compound **1** in DMSO-d_6

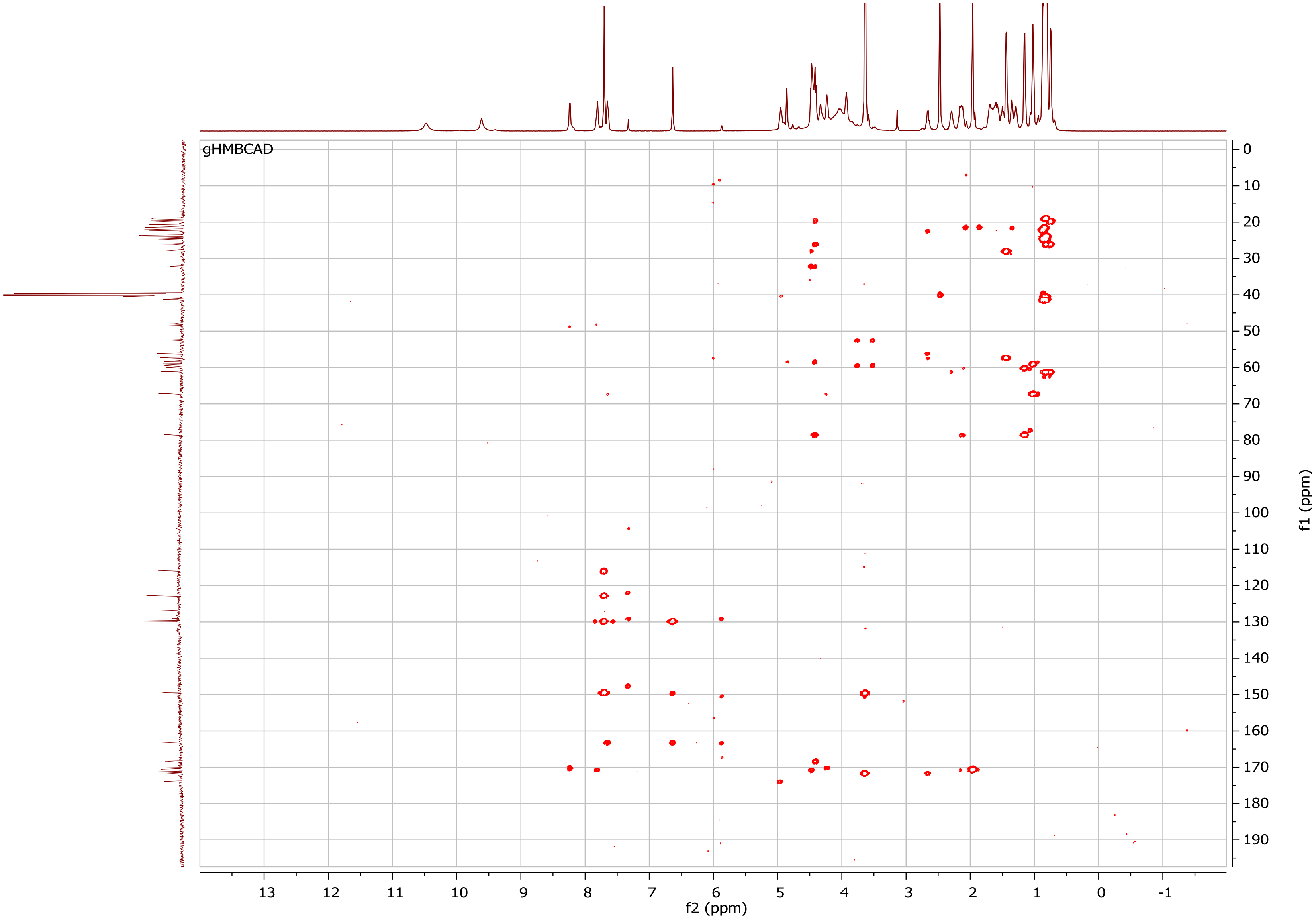

Figure S6. TOCSY spectrum of compound **1** in DMSO-d_6

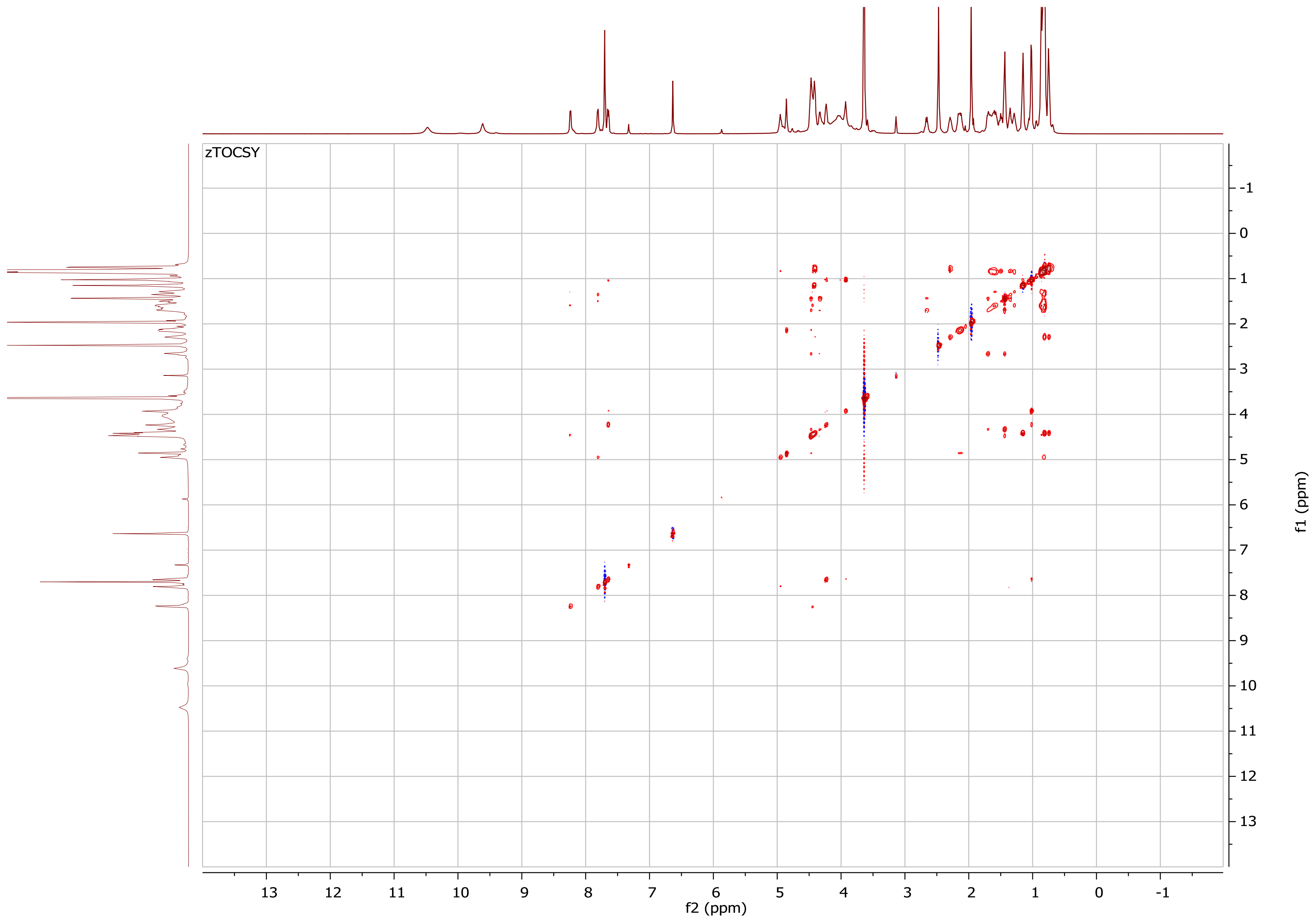

Figure S7. COSY spectrum of compound **1** in DMSO-d_6

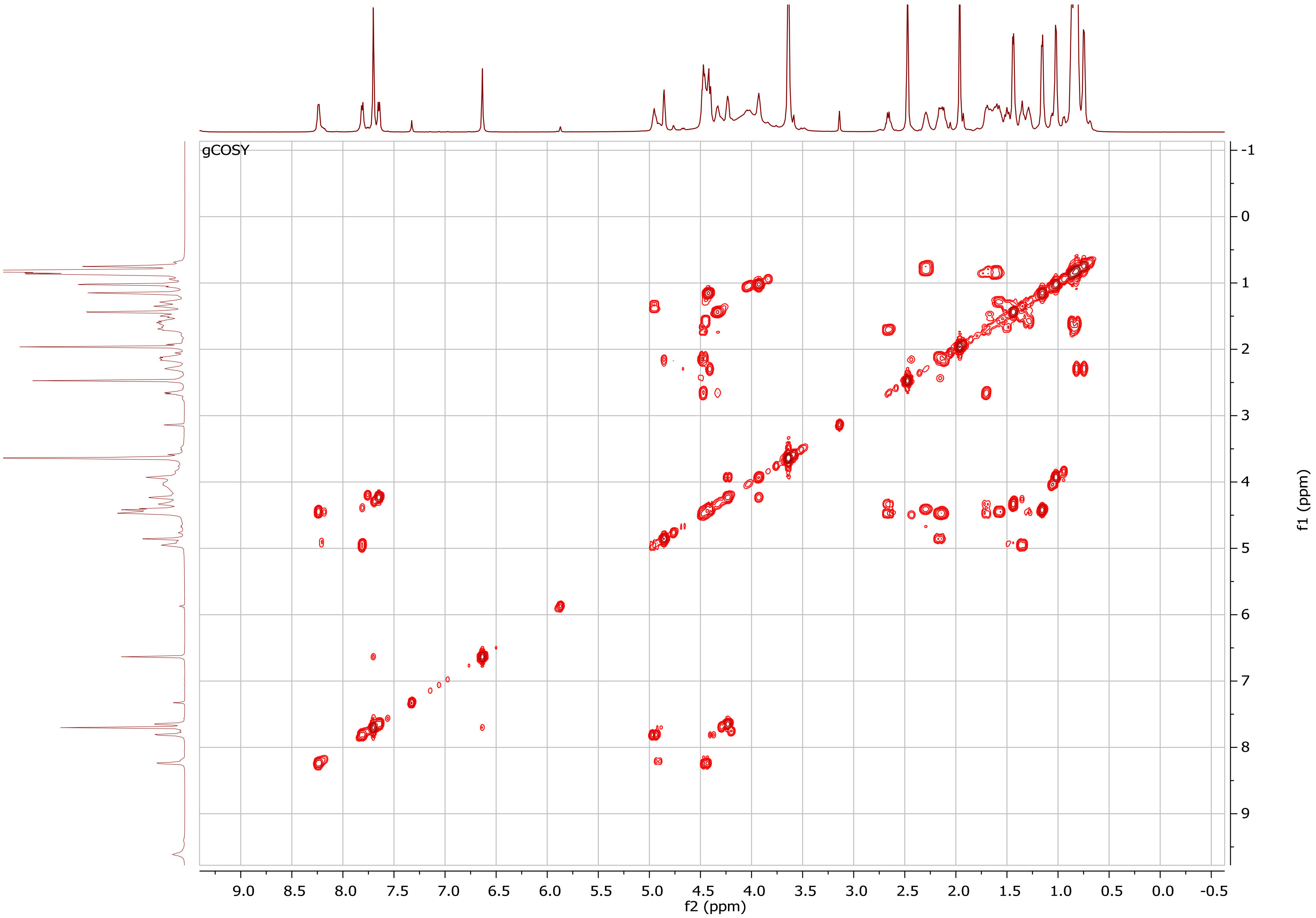

Figure S8. ROESY spectrum of compound **1** in DMSO-d_6

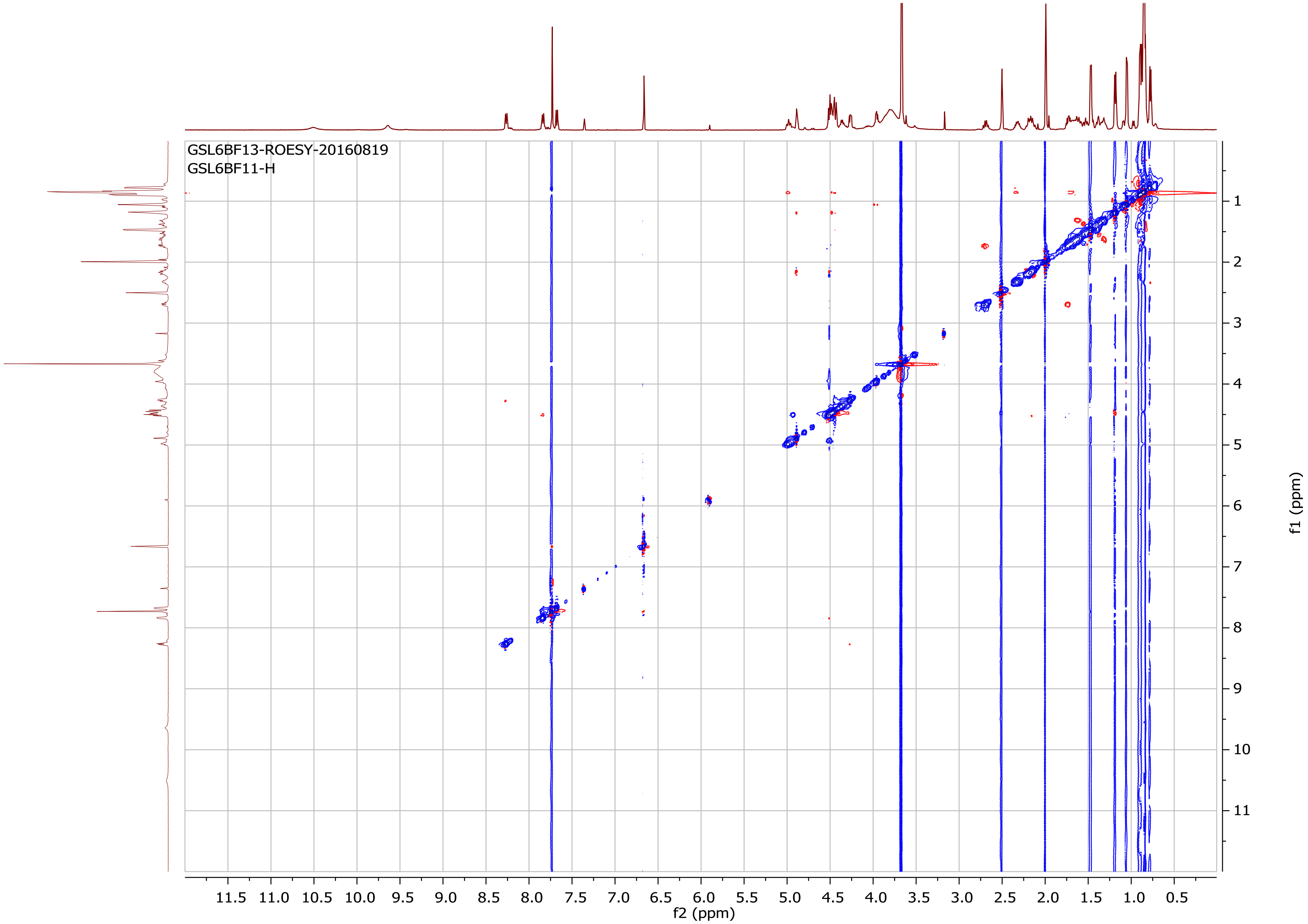

Figure S9. NOESY spectrum of compound **1** in DMSO-d_6

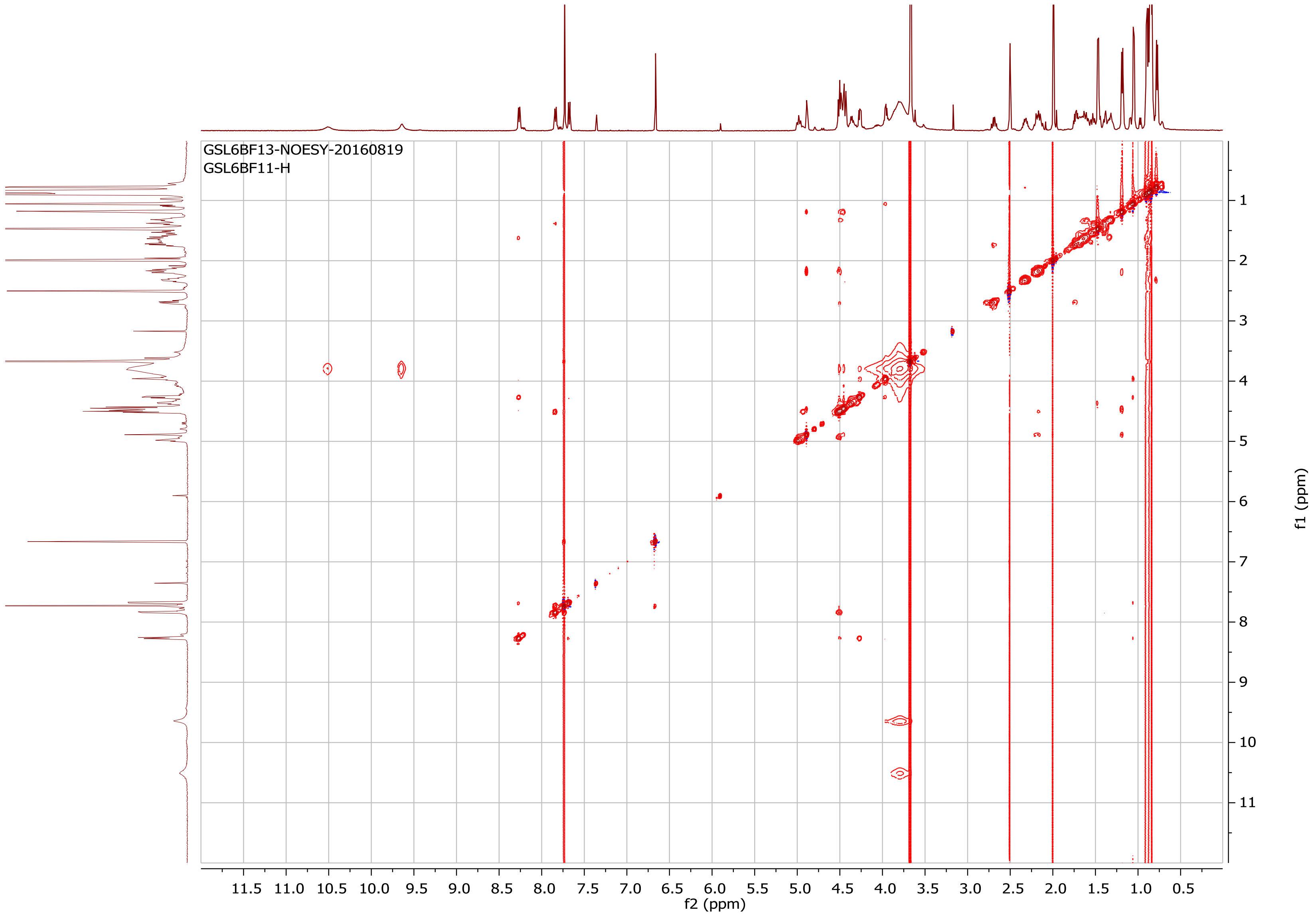

Figure S10.1D NOE spectrum of compound **1** at 4.88 ppm DMSO-d_6

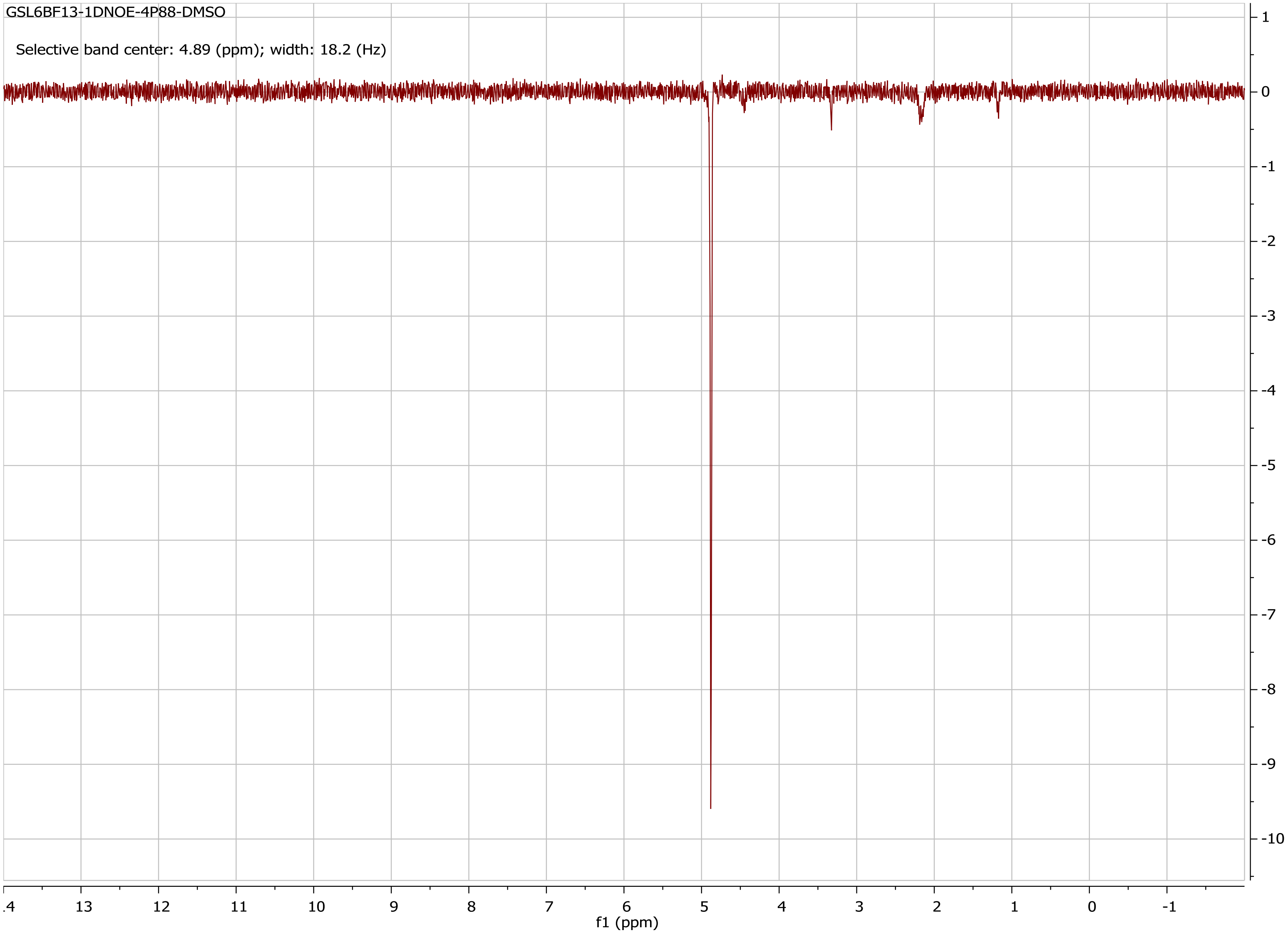

Figure S11.1D NOE spectrum of compound **1** at 4.36 ppm DMSO-d_6

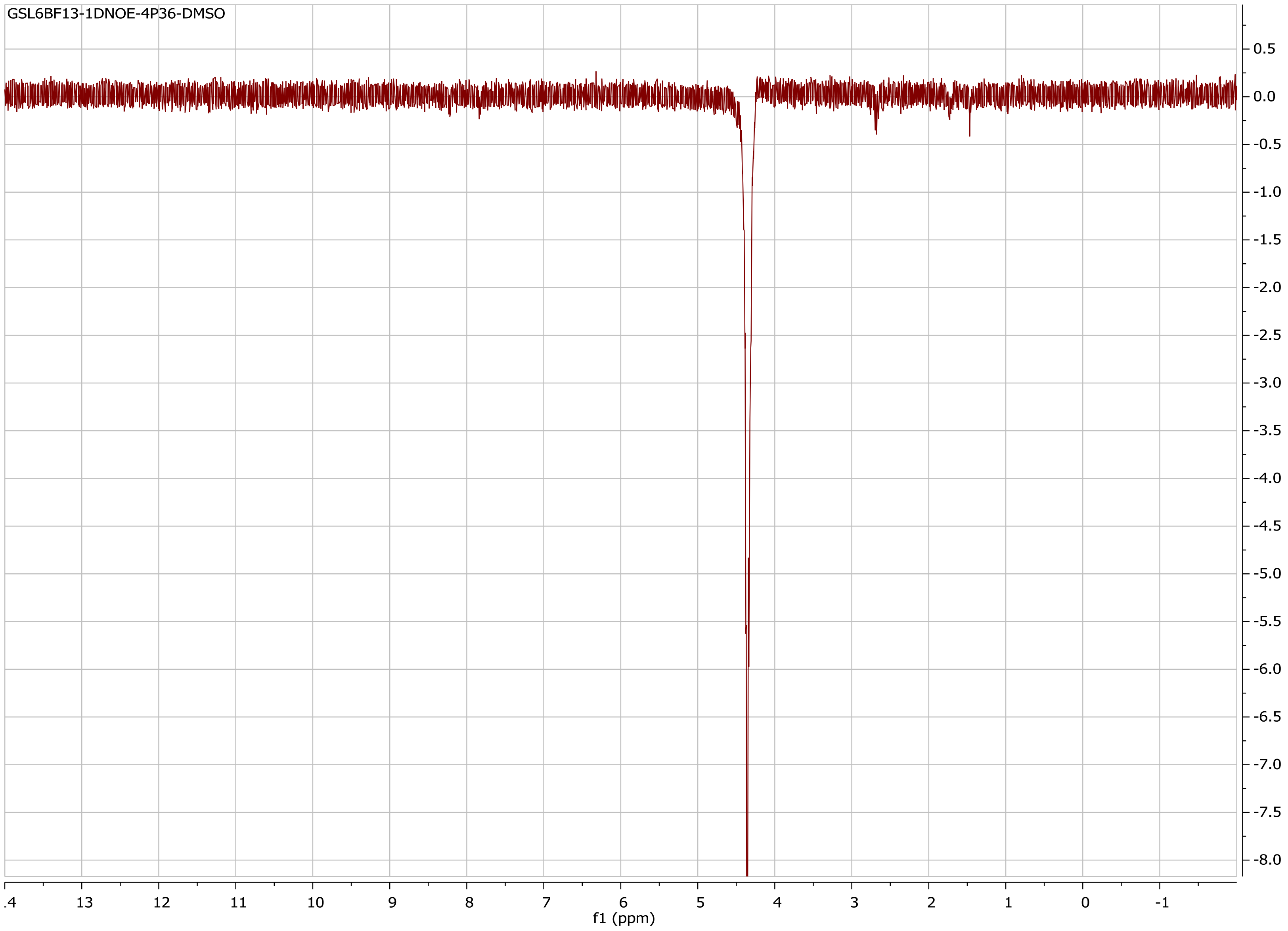

Figure S12.Main Fragment Ions Observed in the HRESIMS/MS Spectrum of compound **1**

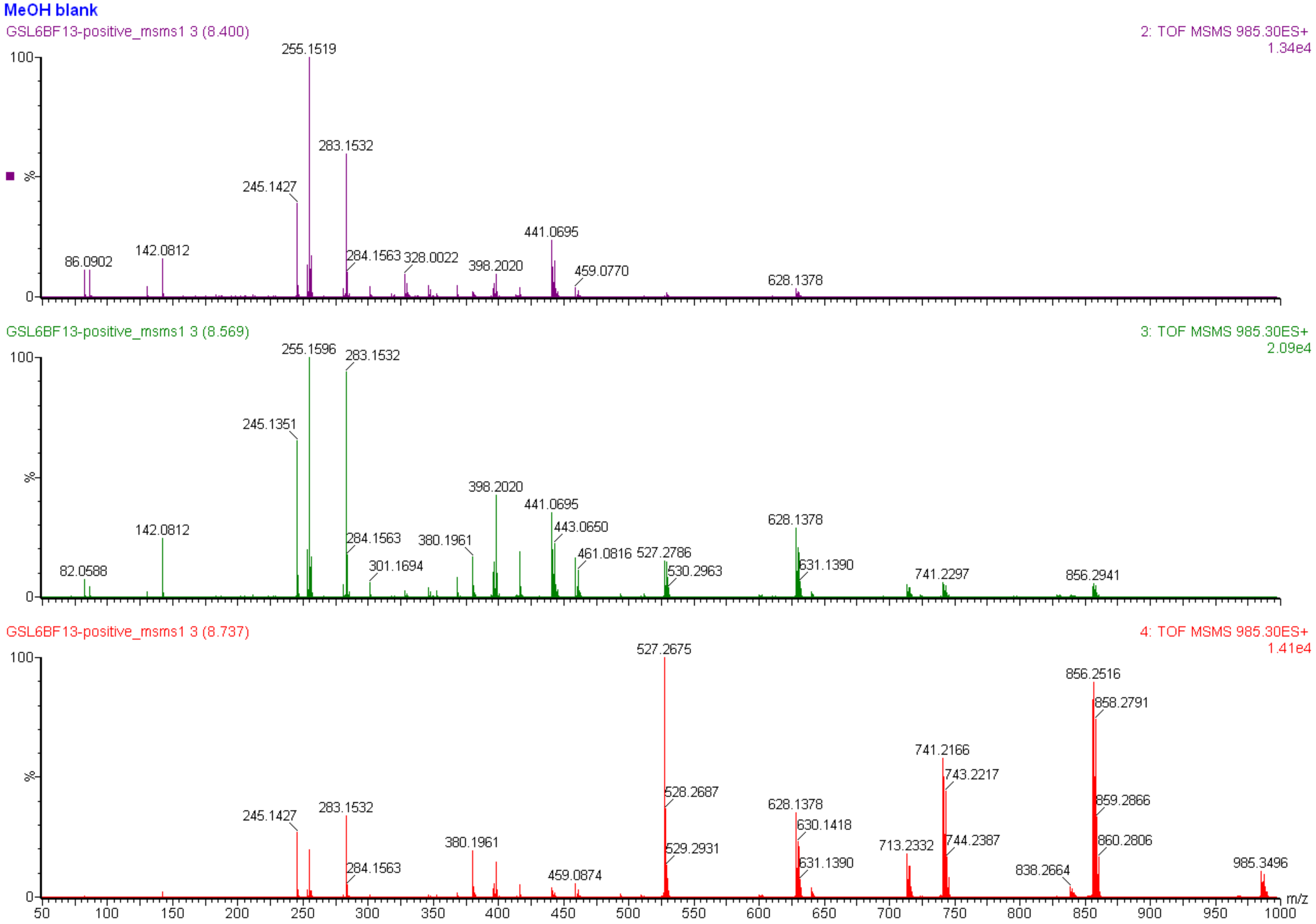

Figure S13. HR(+)ESIMS of compound **1**

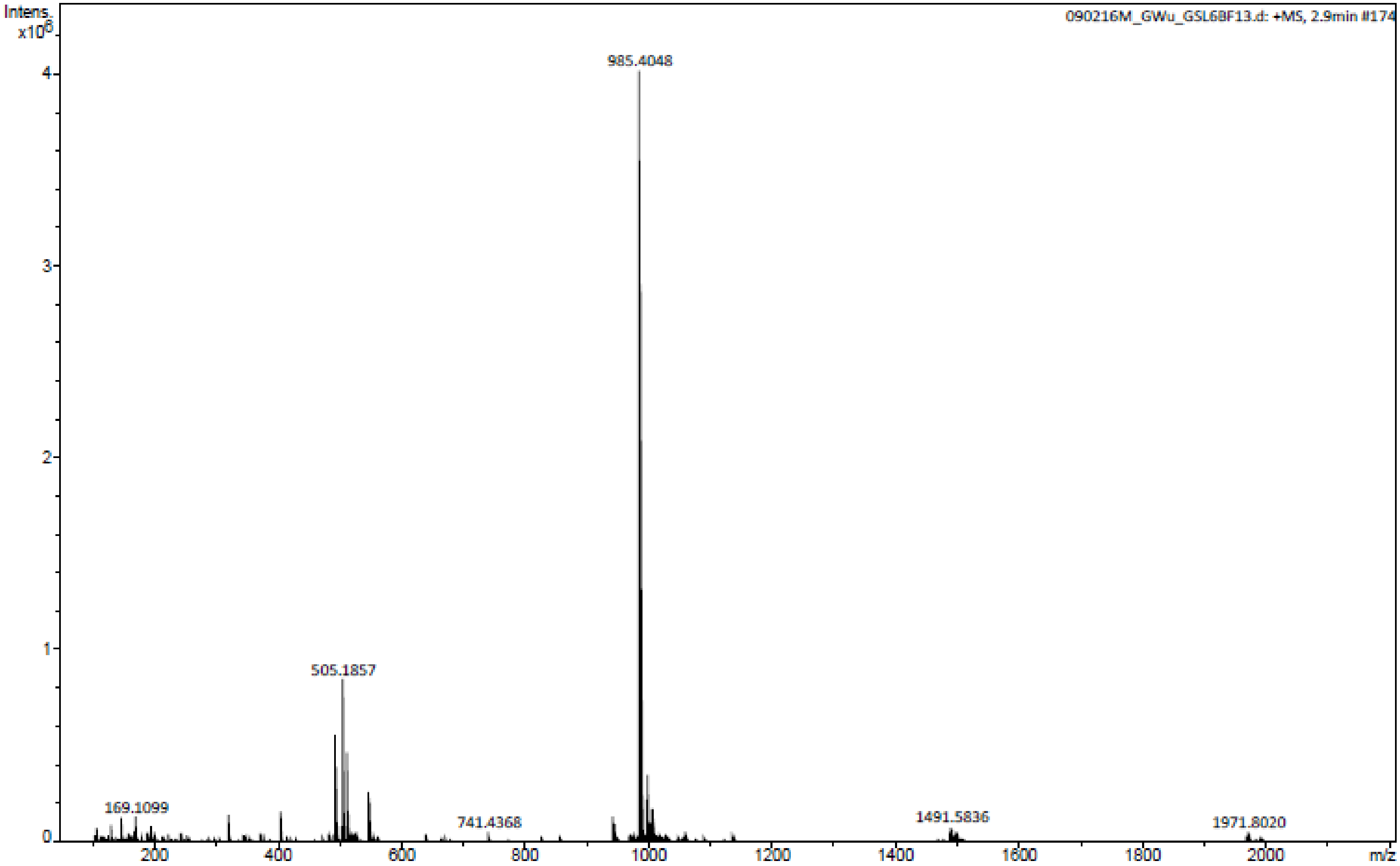

Figure S14. ^{1}H NMR spectrum of compound **2** in DMSO-d_6

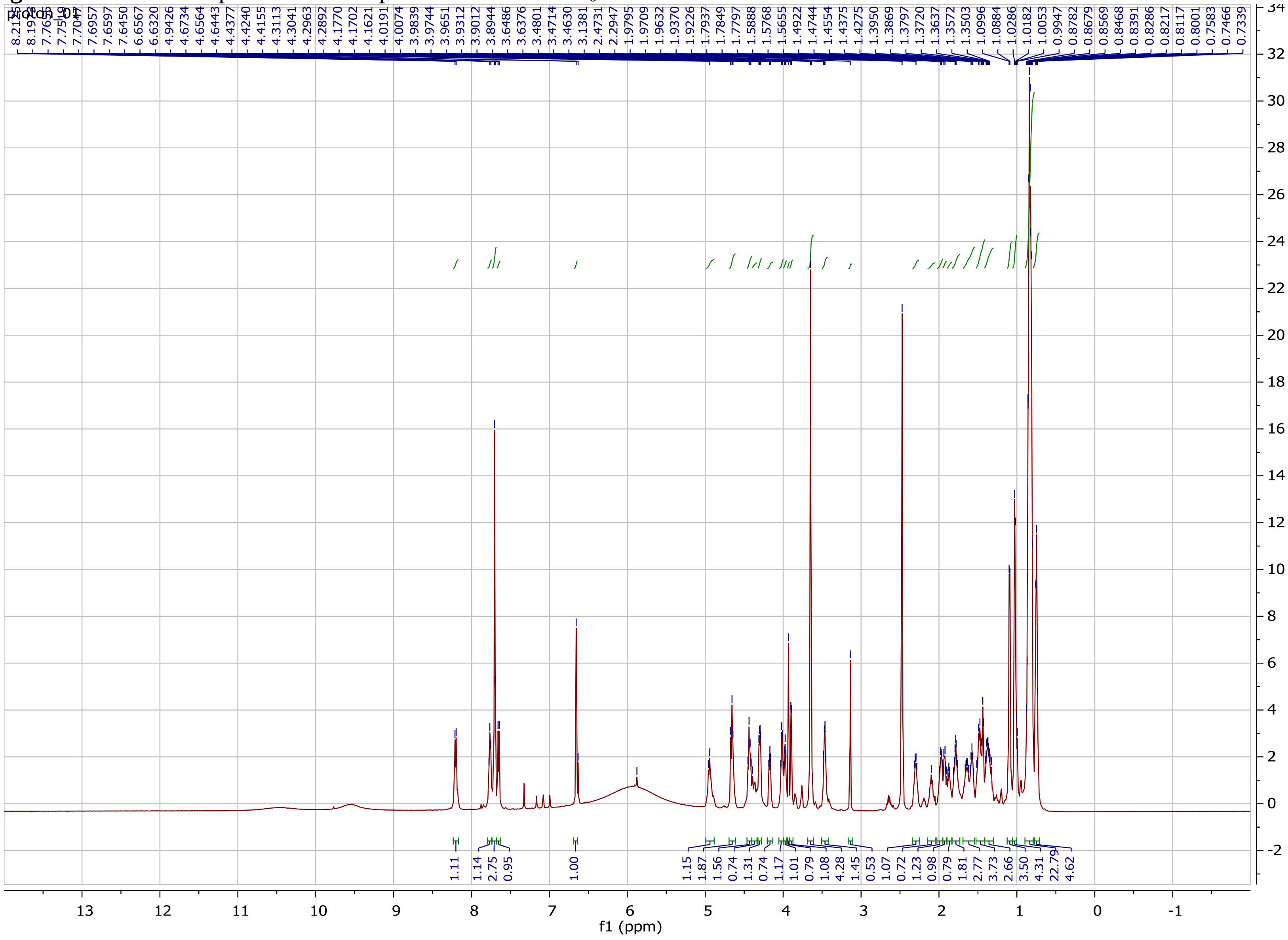

Figure S15. ^{13}C NMR spectrum of compound **2** in DMSO-d_6

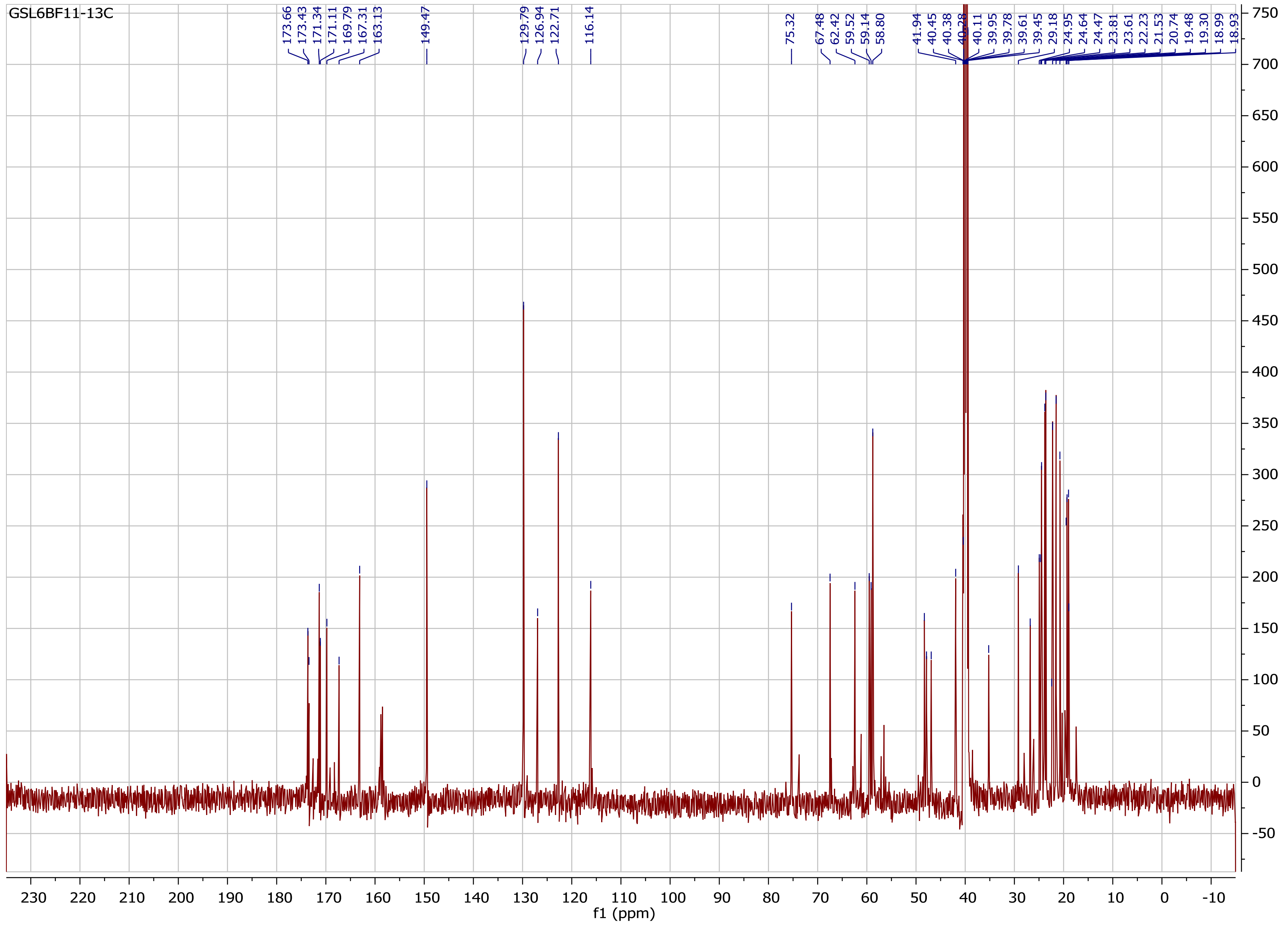

Figure S16.gHSQCAD spectrum of compound **2** in DMSO-d_6

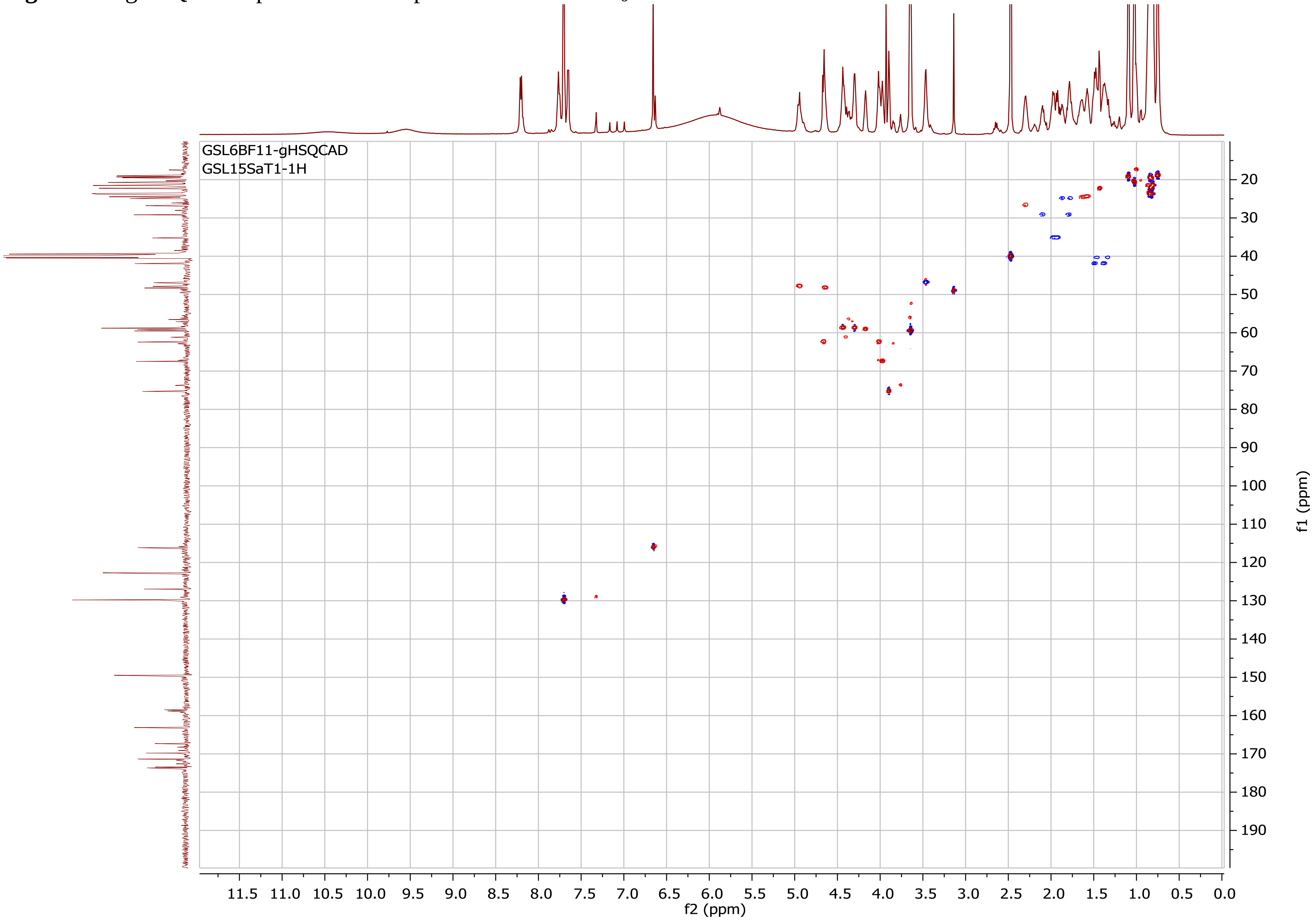

Figure S17.gHMBCAD spectrum of compound **2** in DMSO-d_6

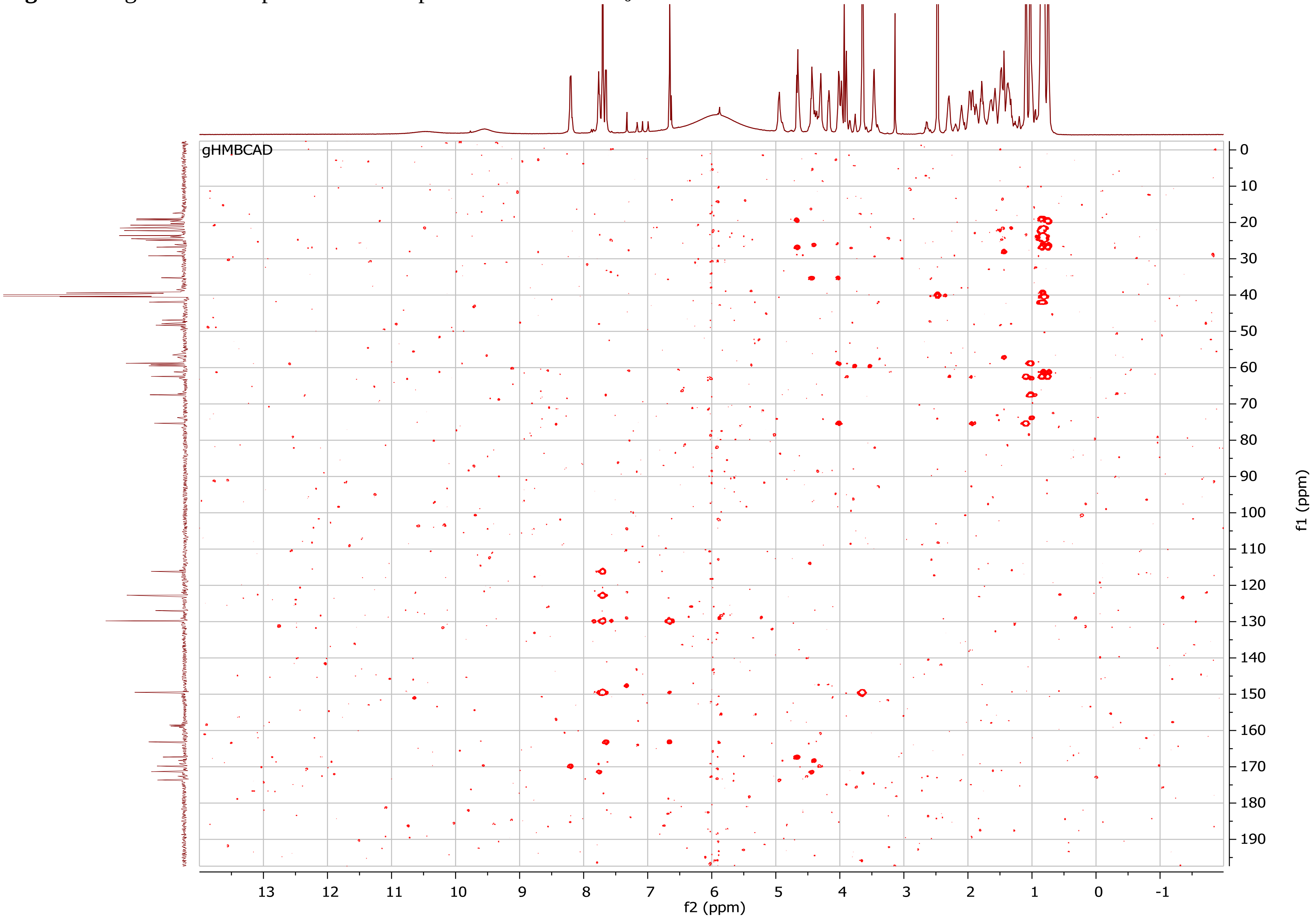

109 **Figure S18**.TOCSY spectrum of compound **2** in DMSO-d_6

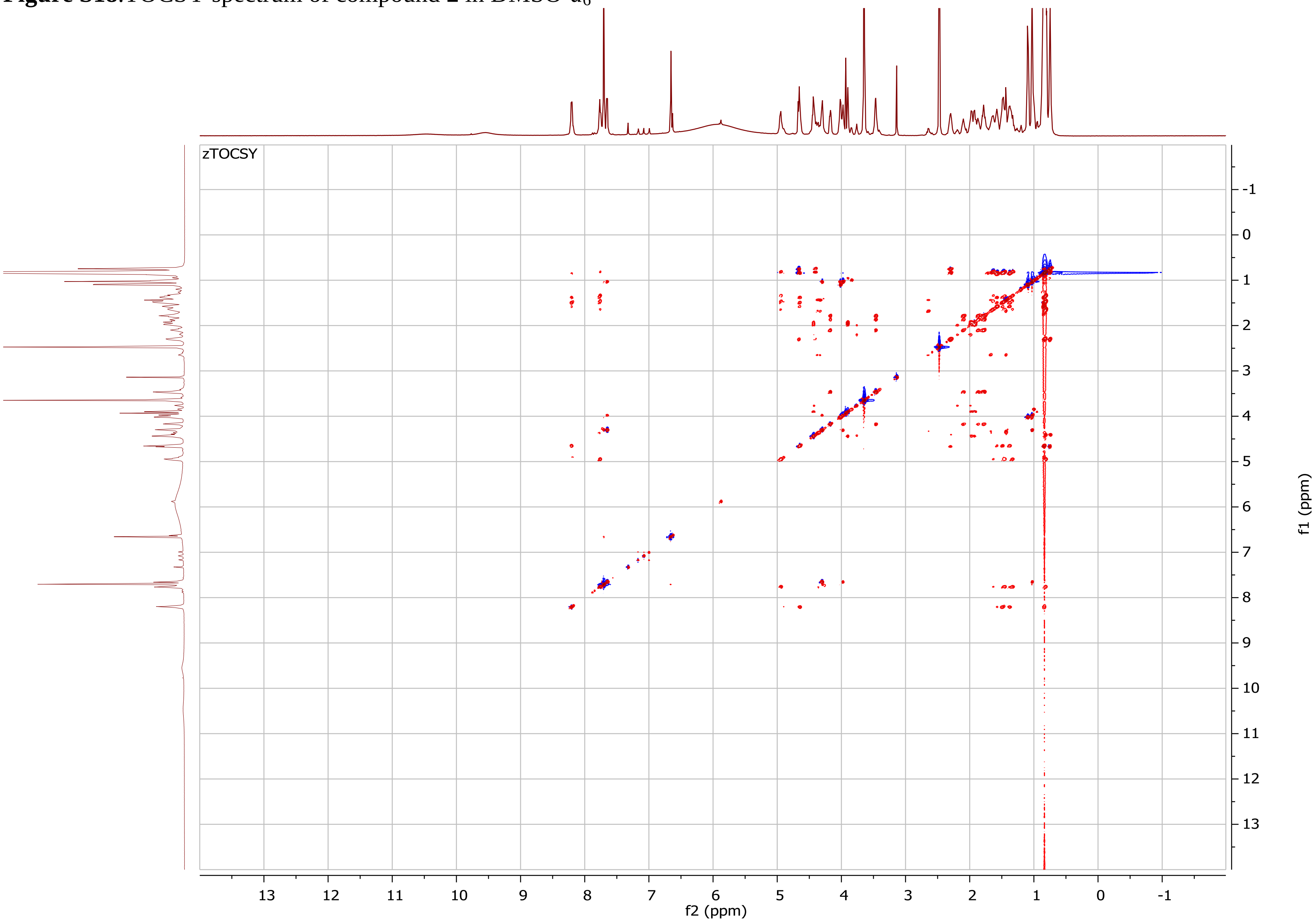

110

111 **Figure S19**.COSY spectrum of compound **2** in DMSO-d_6

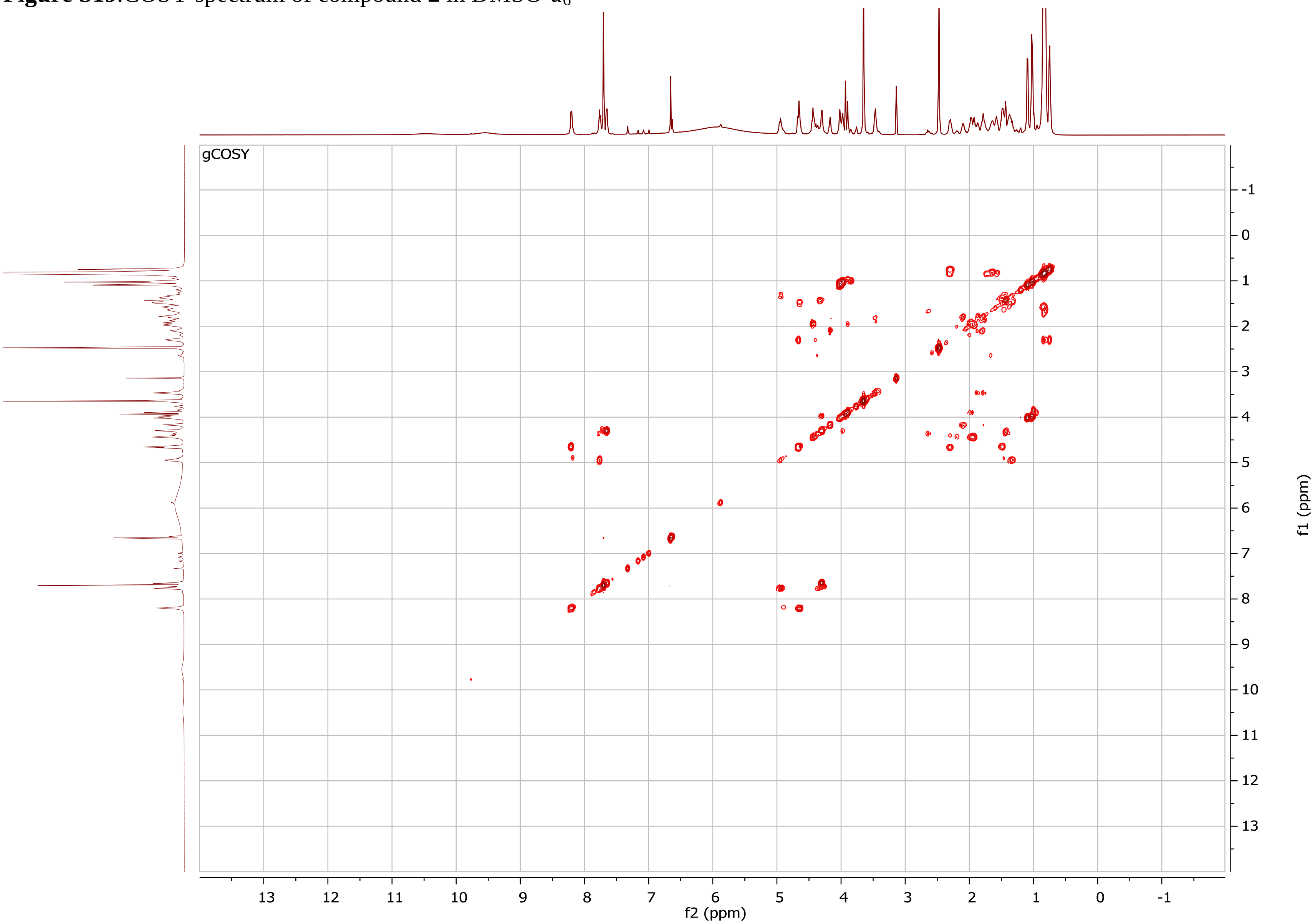

112

113 **Figure S20**. ROESY spectrum of compound **2** in DMSO-d_6

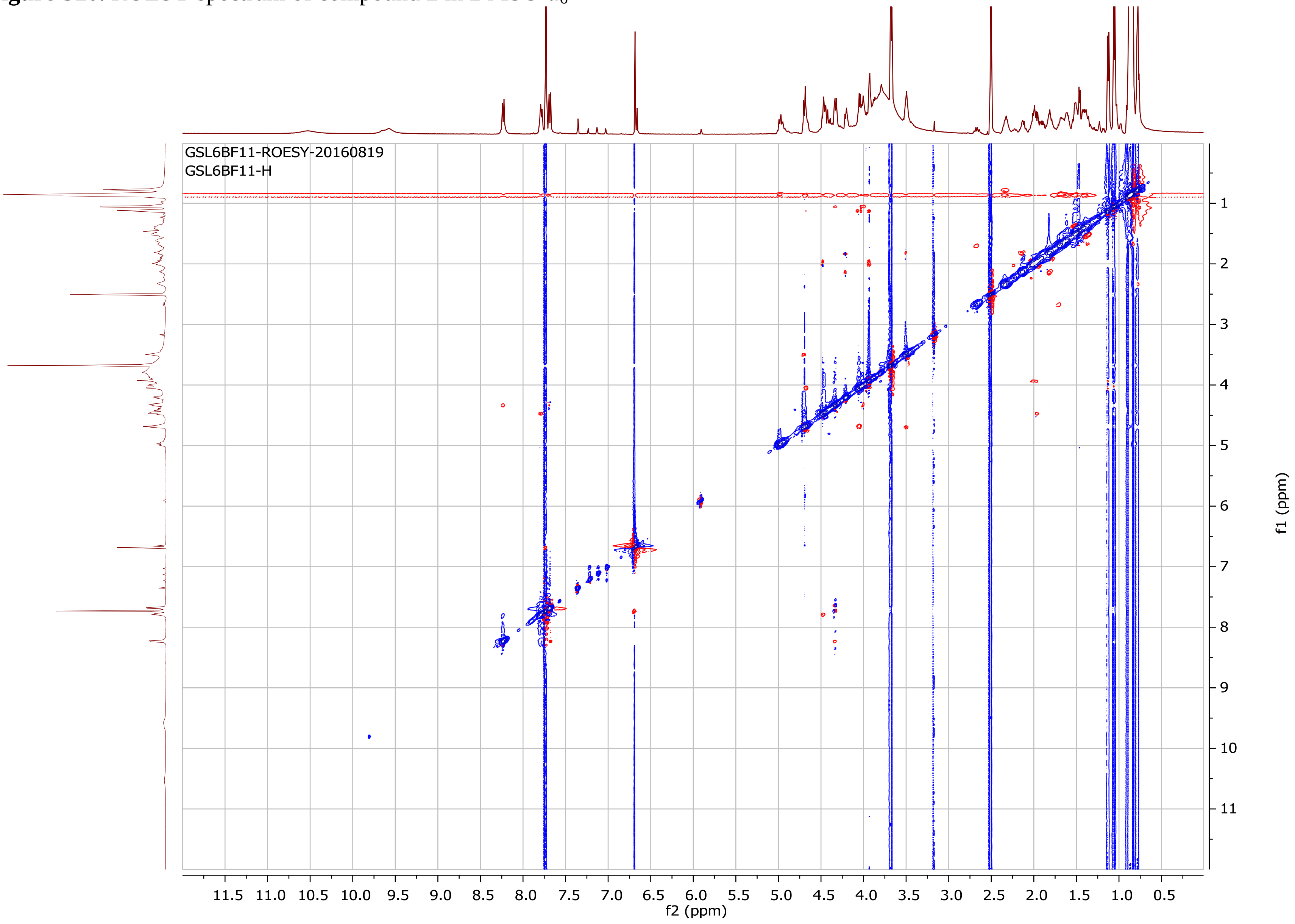

114

115 **Figure S21**.NOESY spectrum of compound **2** in DMSO-d_6

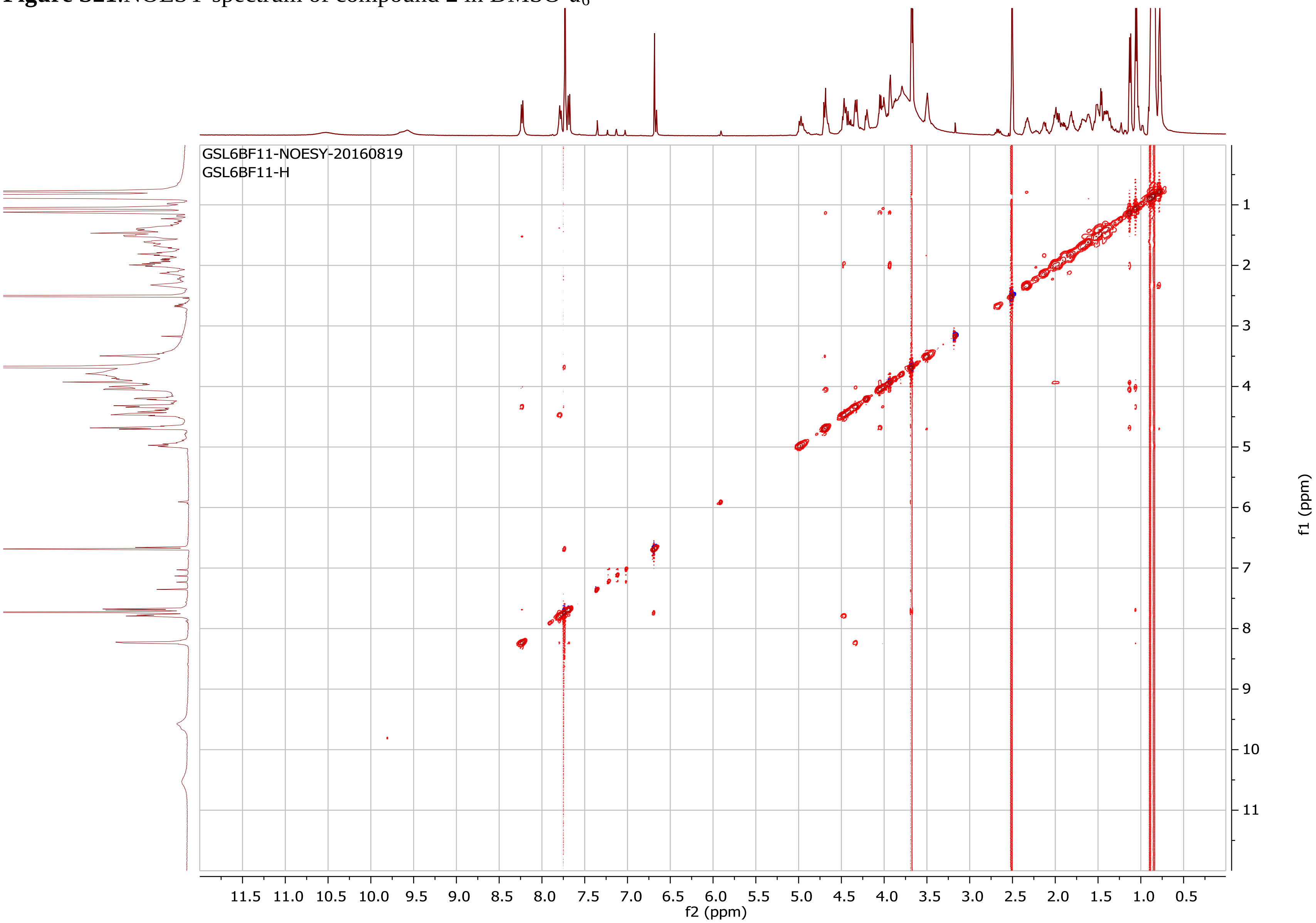

116

Figure S22.1D NOE spectrum of compound **2** at 3.92 ppm DMSO-d_6

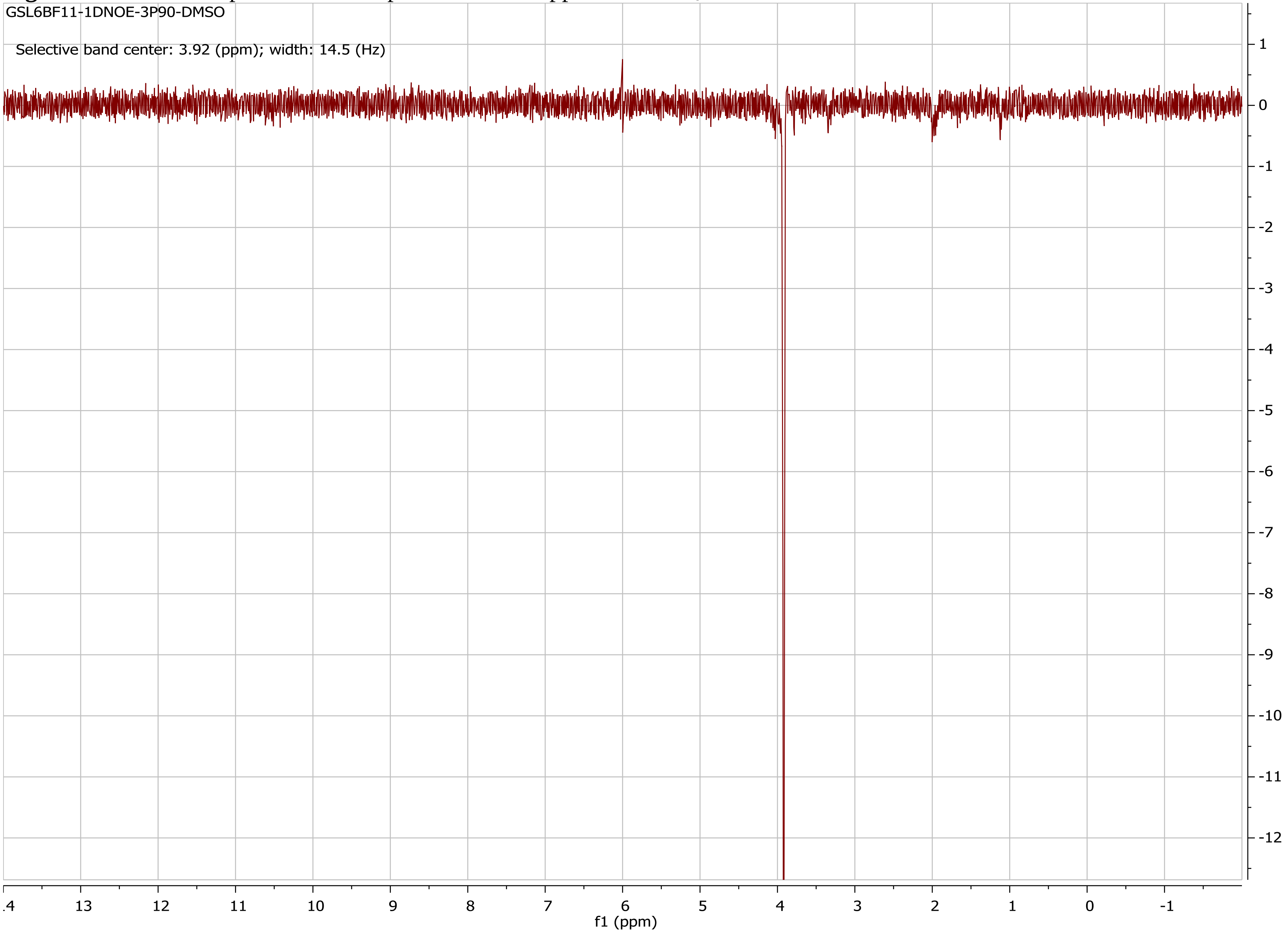

Figure S23. Main fragment ions observed in the HRESIMS/MS Spectrum of compound **2**

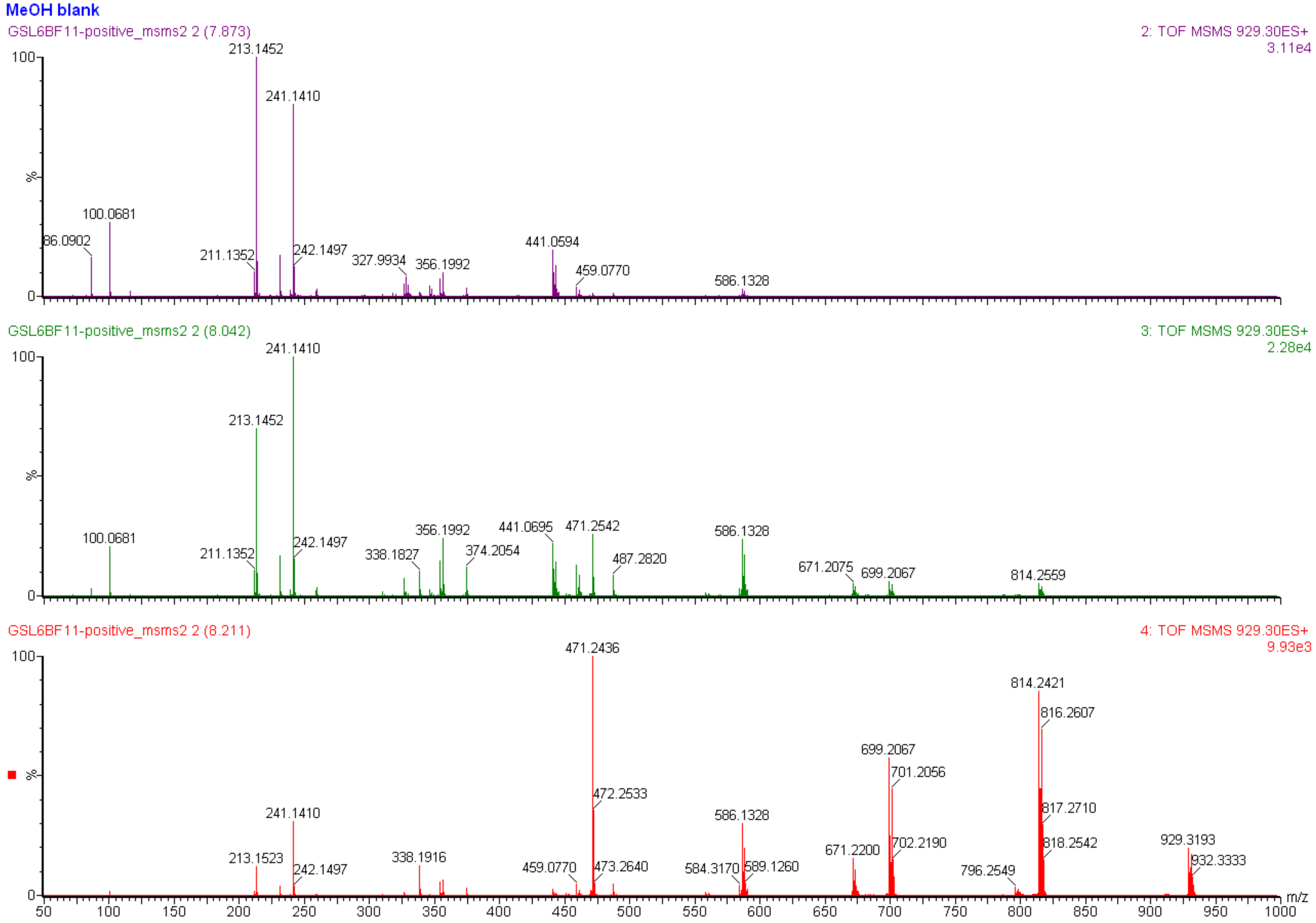

Figure S24. HR(+)ESIMS of compound **2**

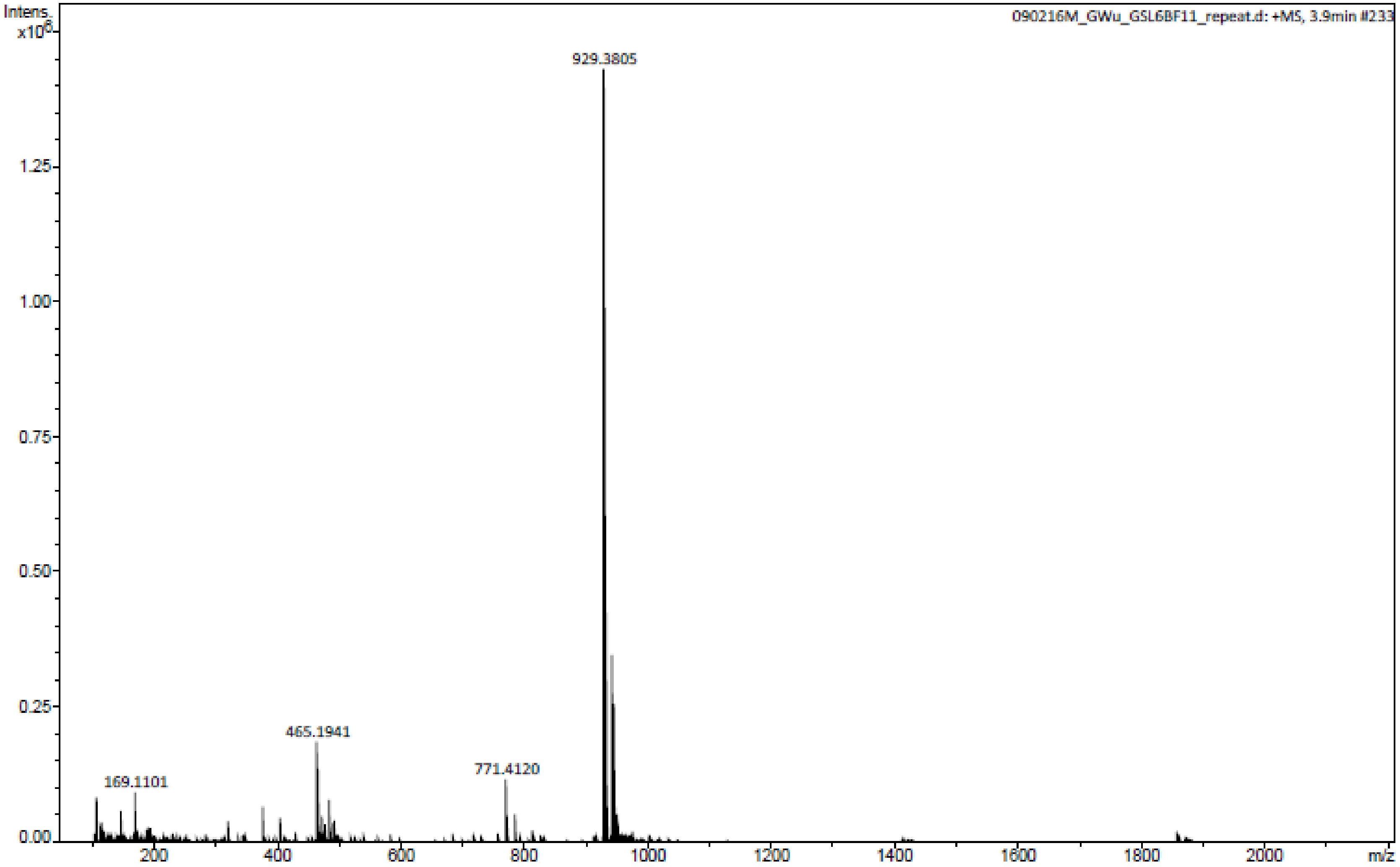

Figure S25.^{1}H NMR spectrum of compound **3** in DMSO-d_6

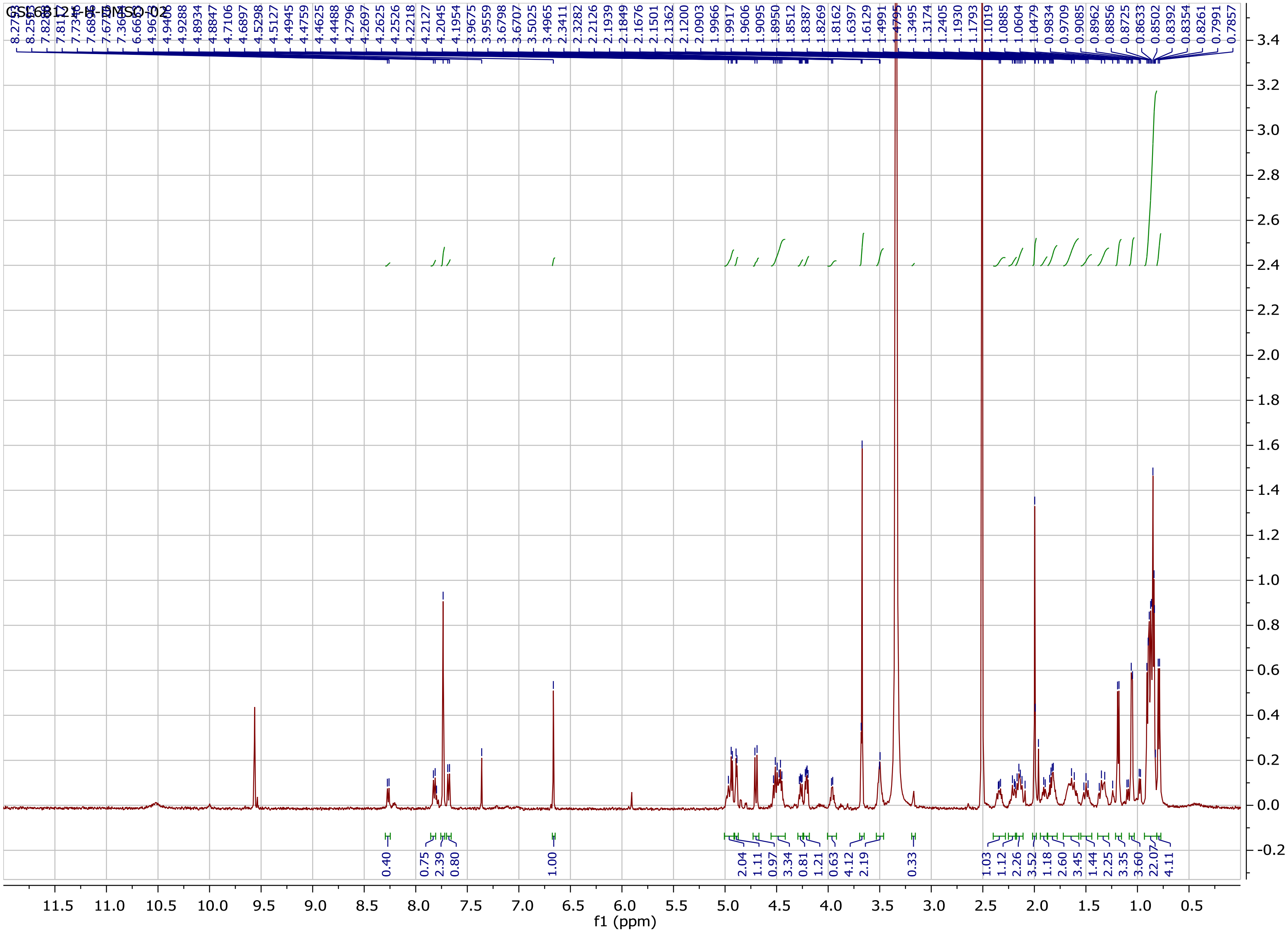

Figure S26. gHSQCAD spectrum of compound **3** in DMSO-d_6

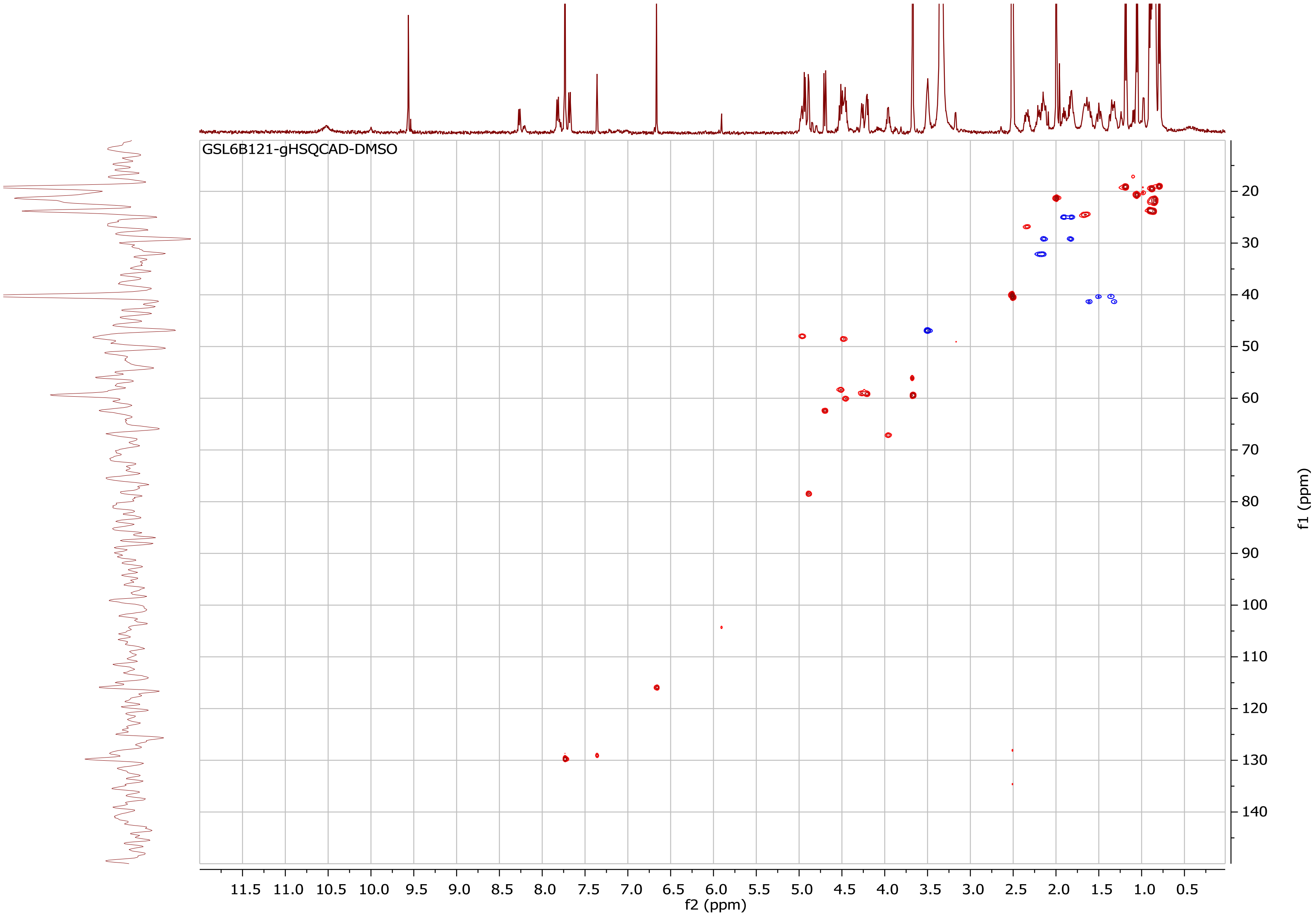

Figure S27.gHMBCAD spectrum of compound **3** in DMSO-d_6

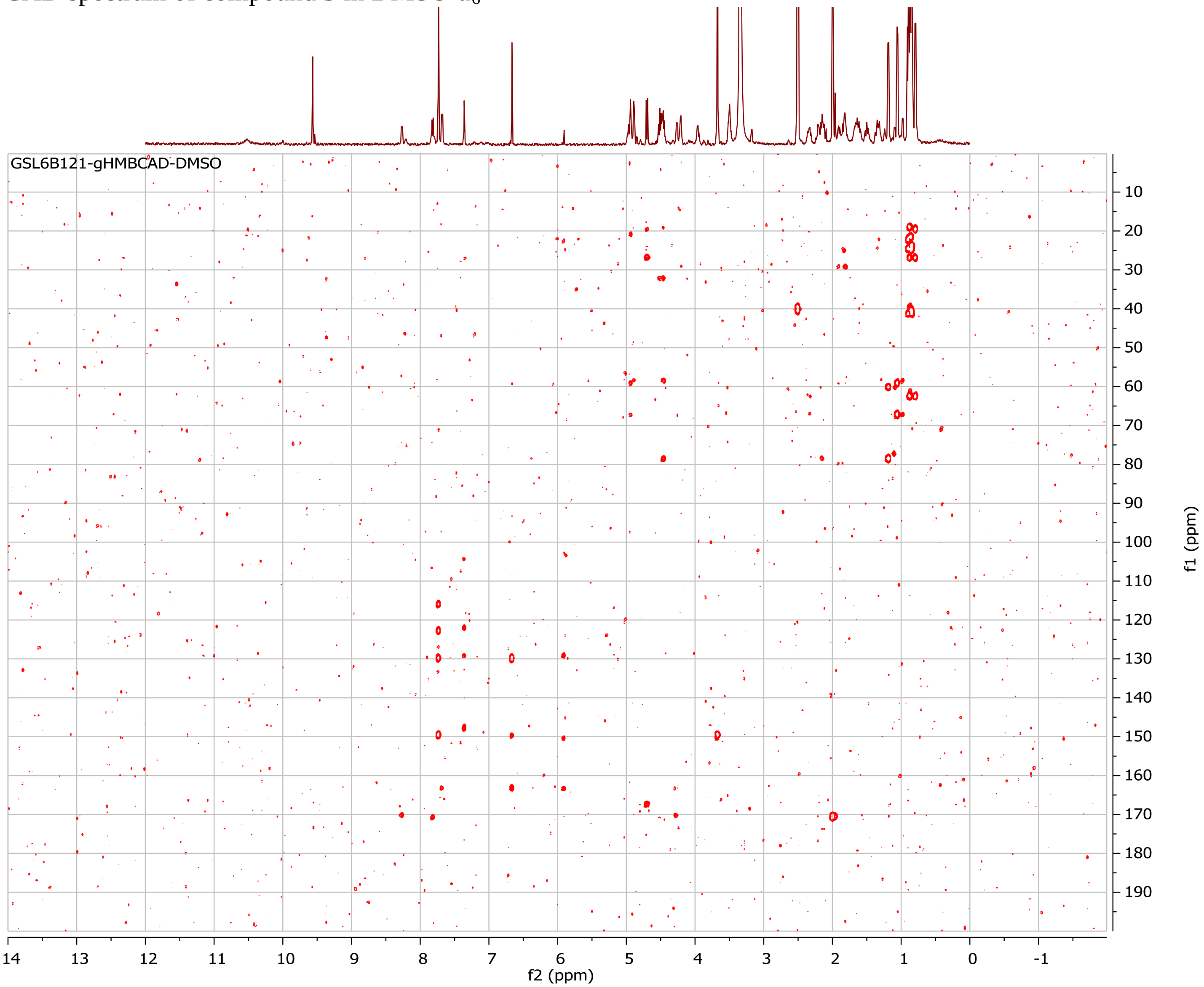

136 **Figure S28**. TOCSY spectrum of compound **3** in DMSO-d_6

137

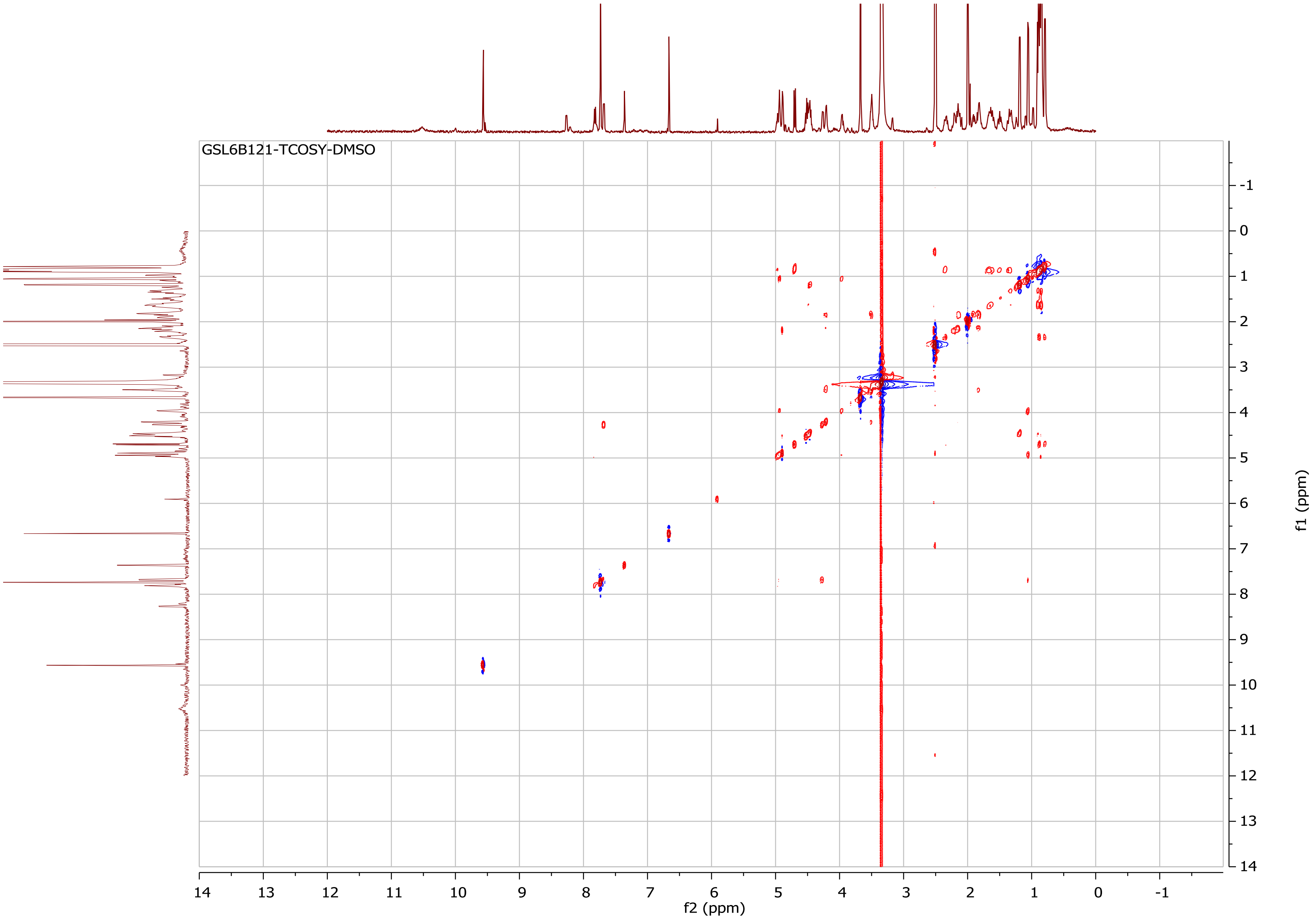

138

Figure S29.COSY spectrum of compound **3** in DMSO-d_6

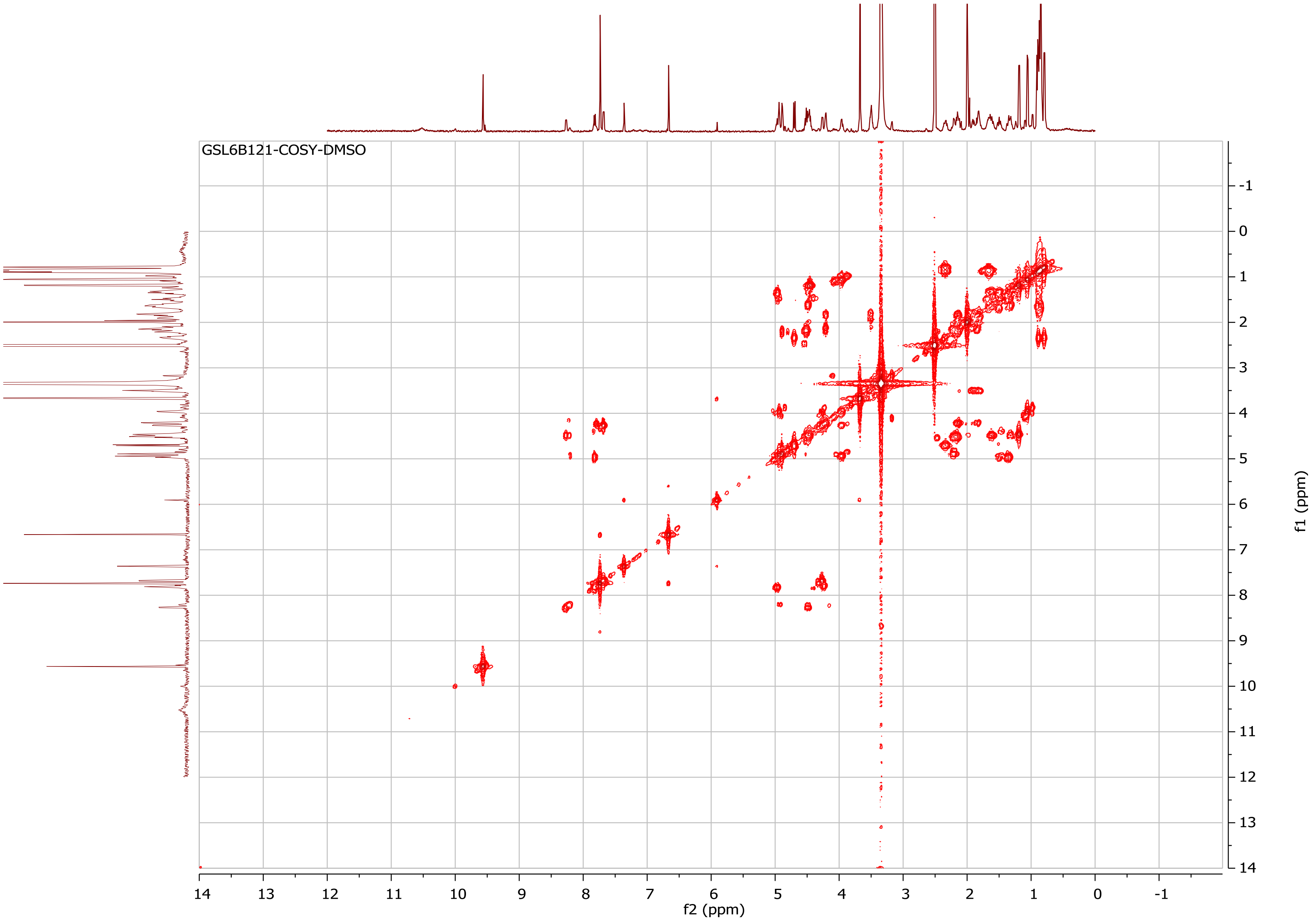

Figure S30. ROESY spectrum of compound **3** in DMSO-d_6

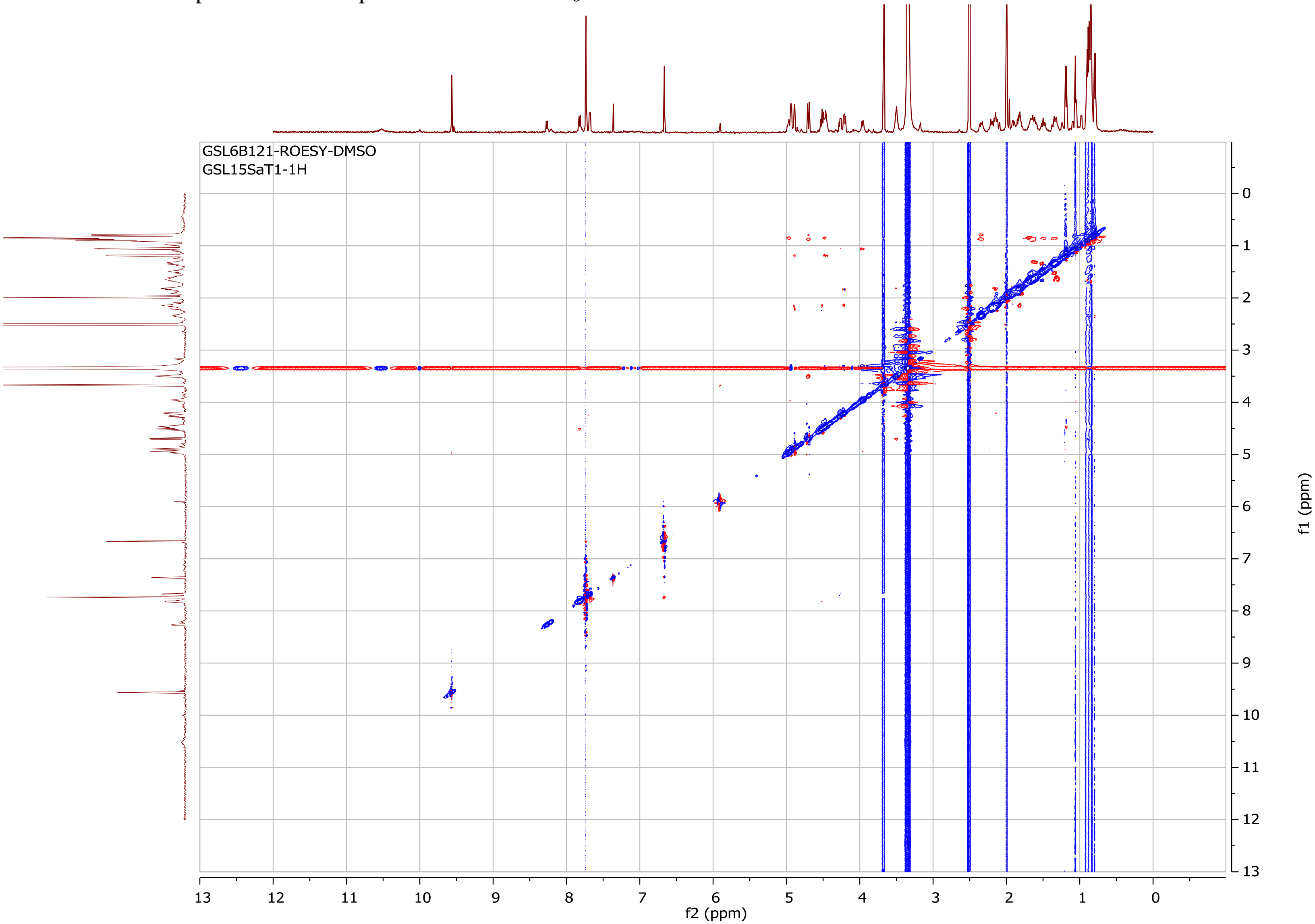

Figure S31.NOESY spectrum of compound **3** in DMSO-d_6

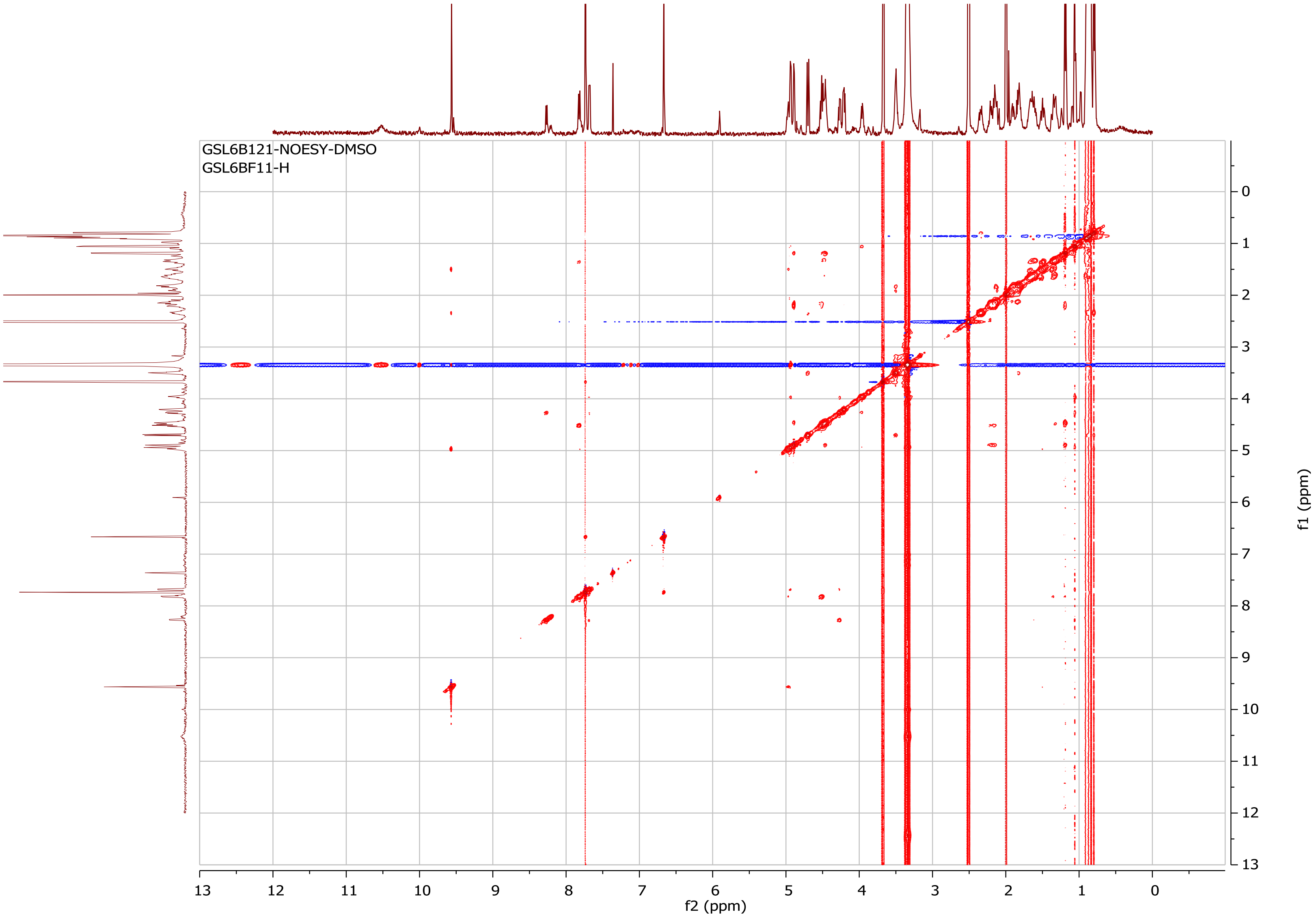

Figure S32. HR(+)ESIMS of compound **3**

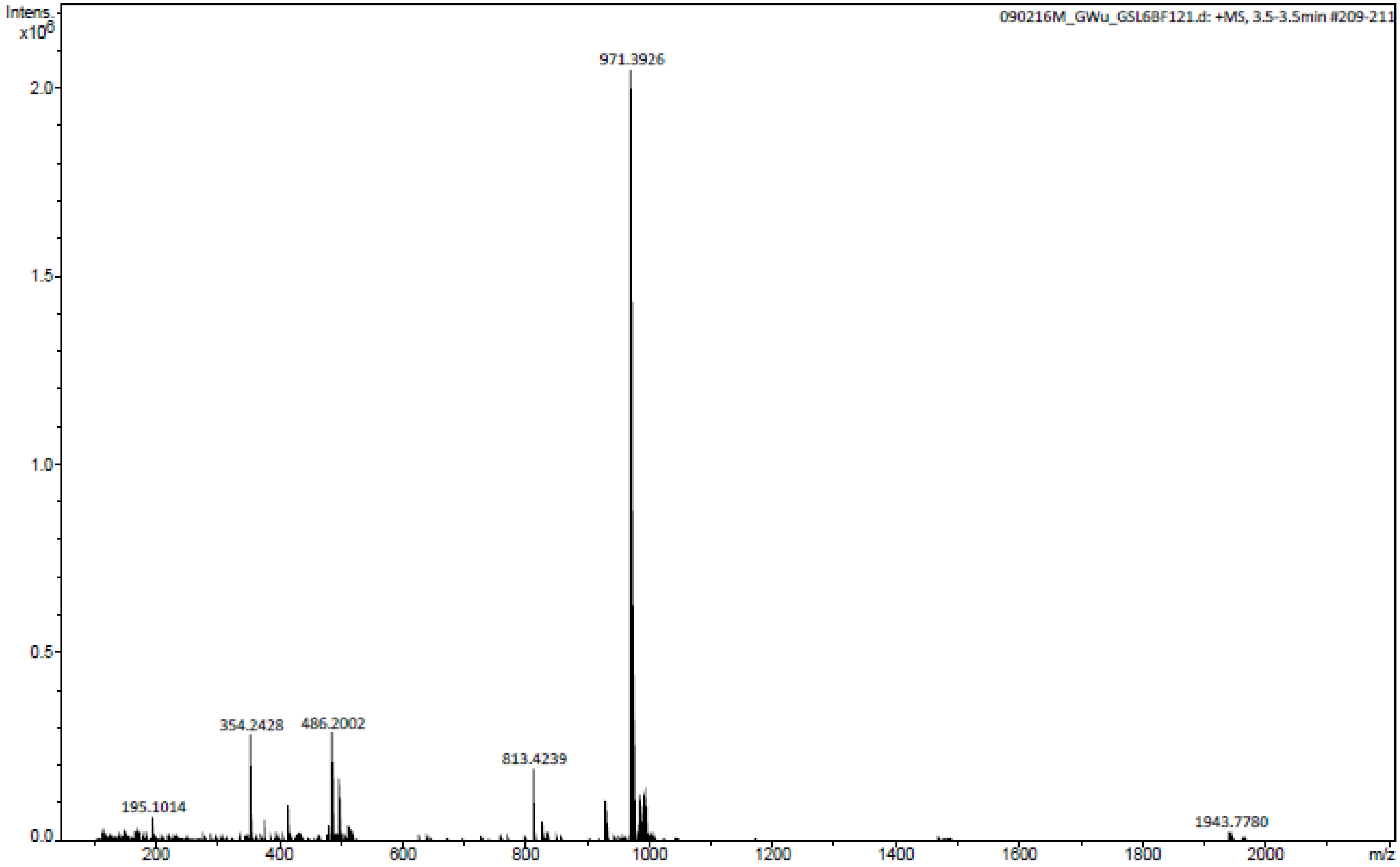

Figure S33.Advanced Marfey's analysis of acid hydrolysate of **1**

A)D,L-FDLA-Threonine derivatives in **1**: 414 $[M+H]^+$

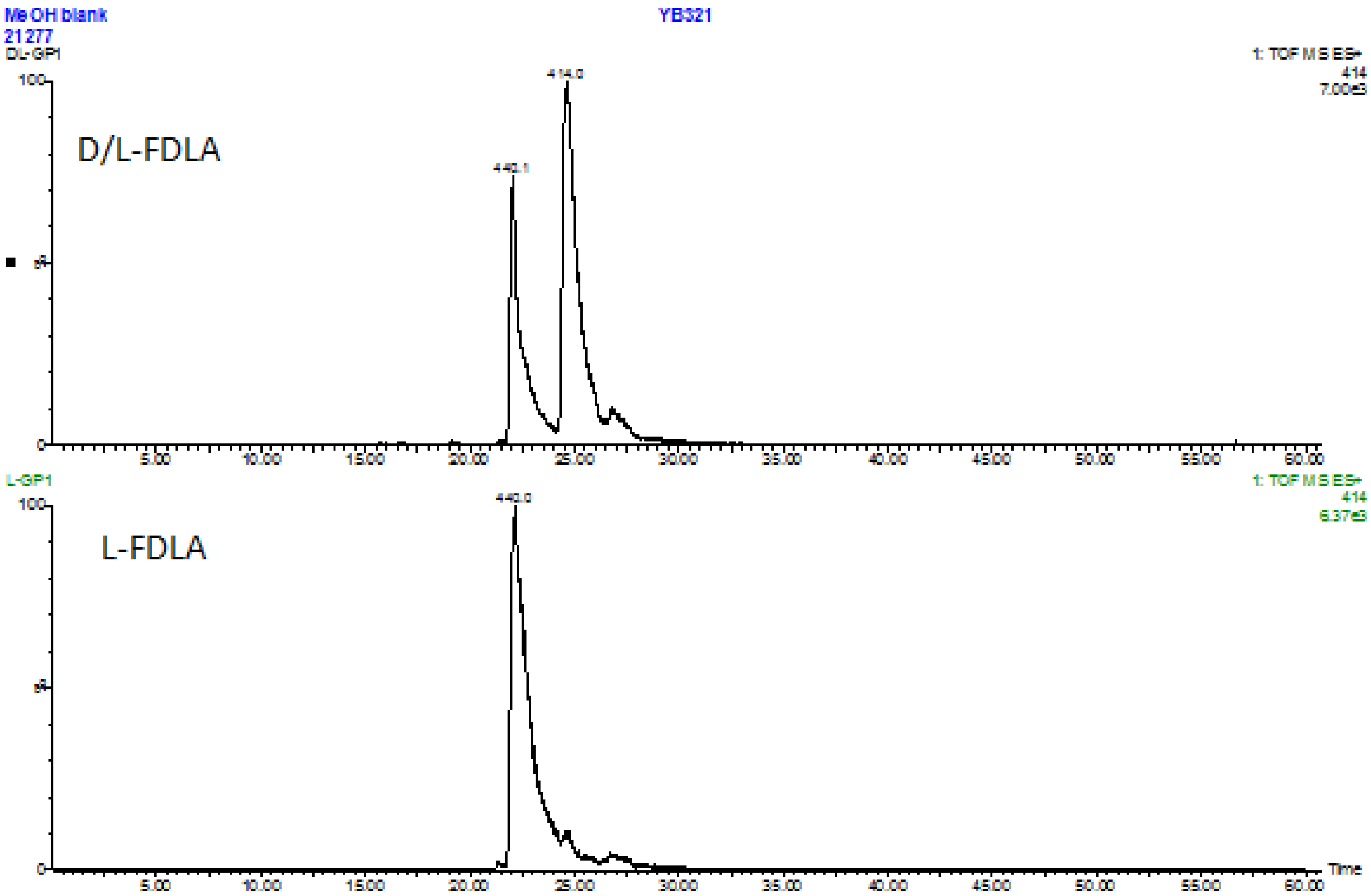

B) D,L-FDLA-Leucine derivatives in **1**: 426 $[M+H]^+$

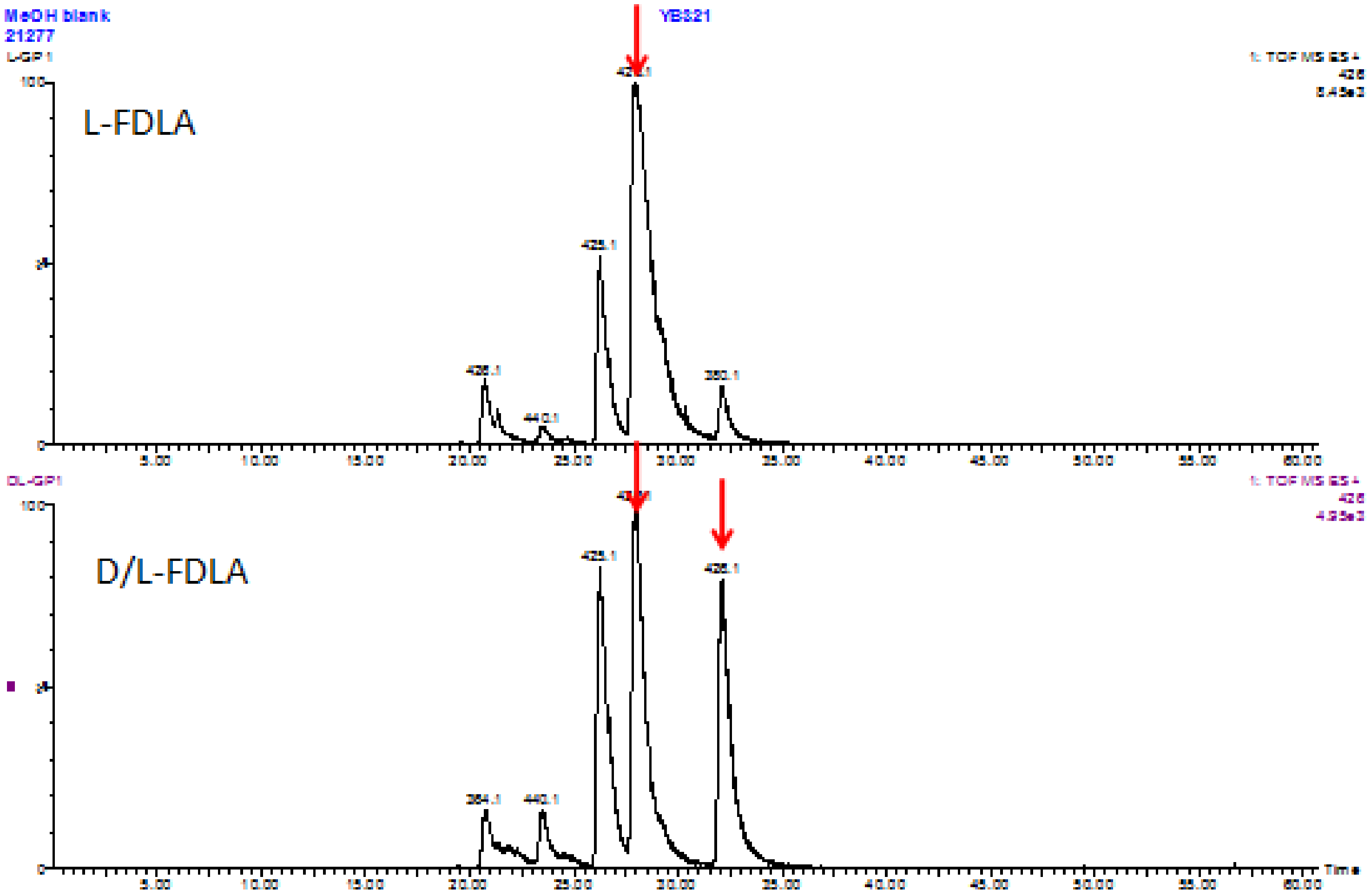

C) D,L-FDLA-HMP derivatives in **1**: 440 $[M+H]^+$

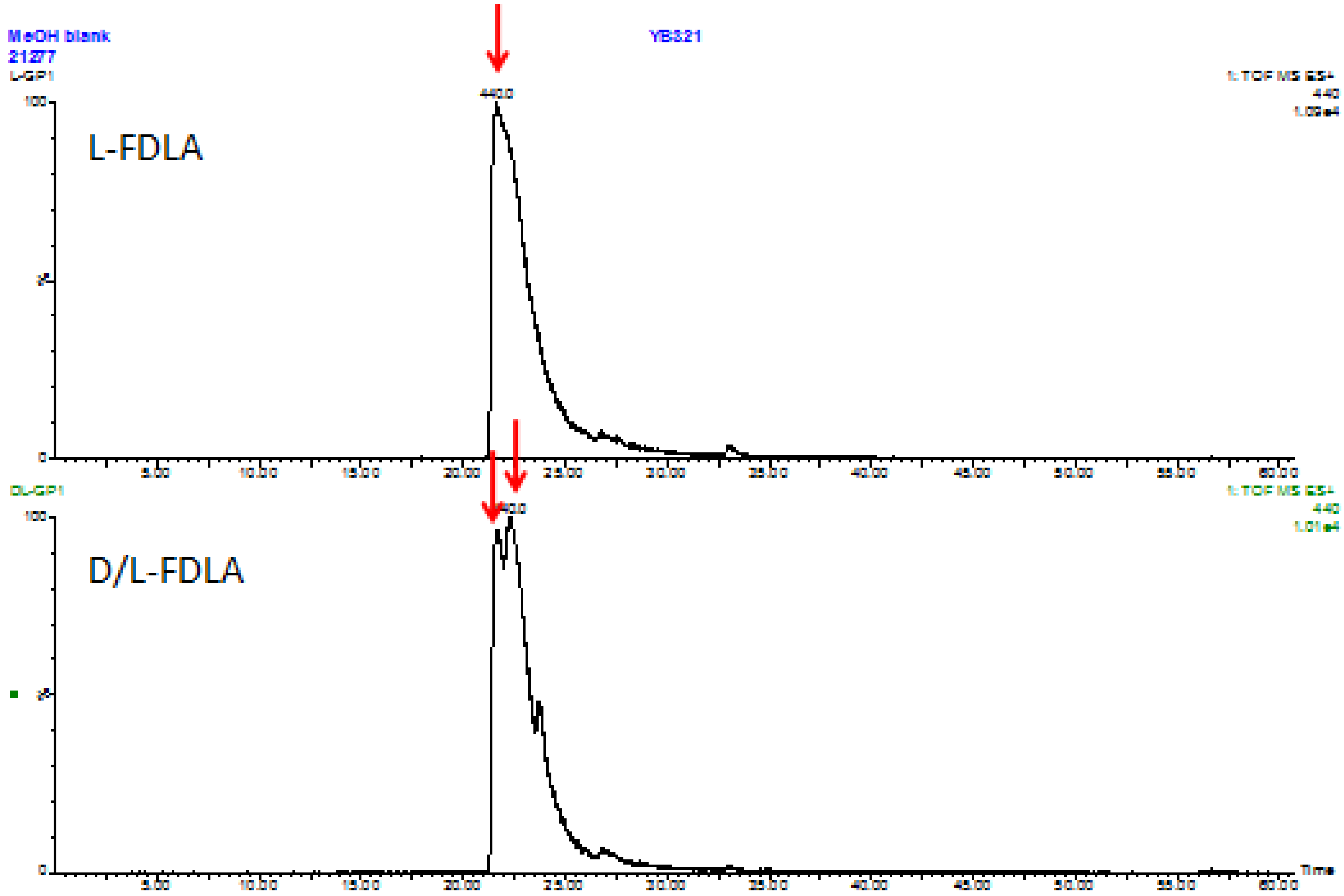

D)D,L-FDLA-Valine derivatives in **1**: 434 $[M+Na]^+$

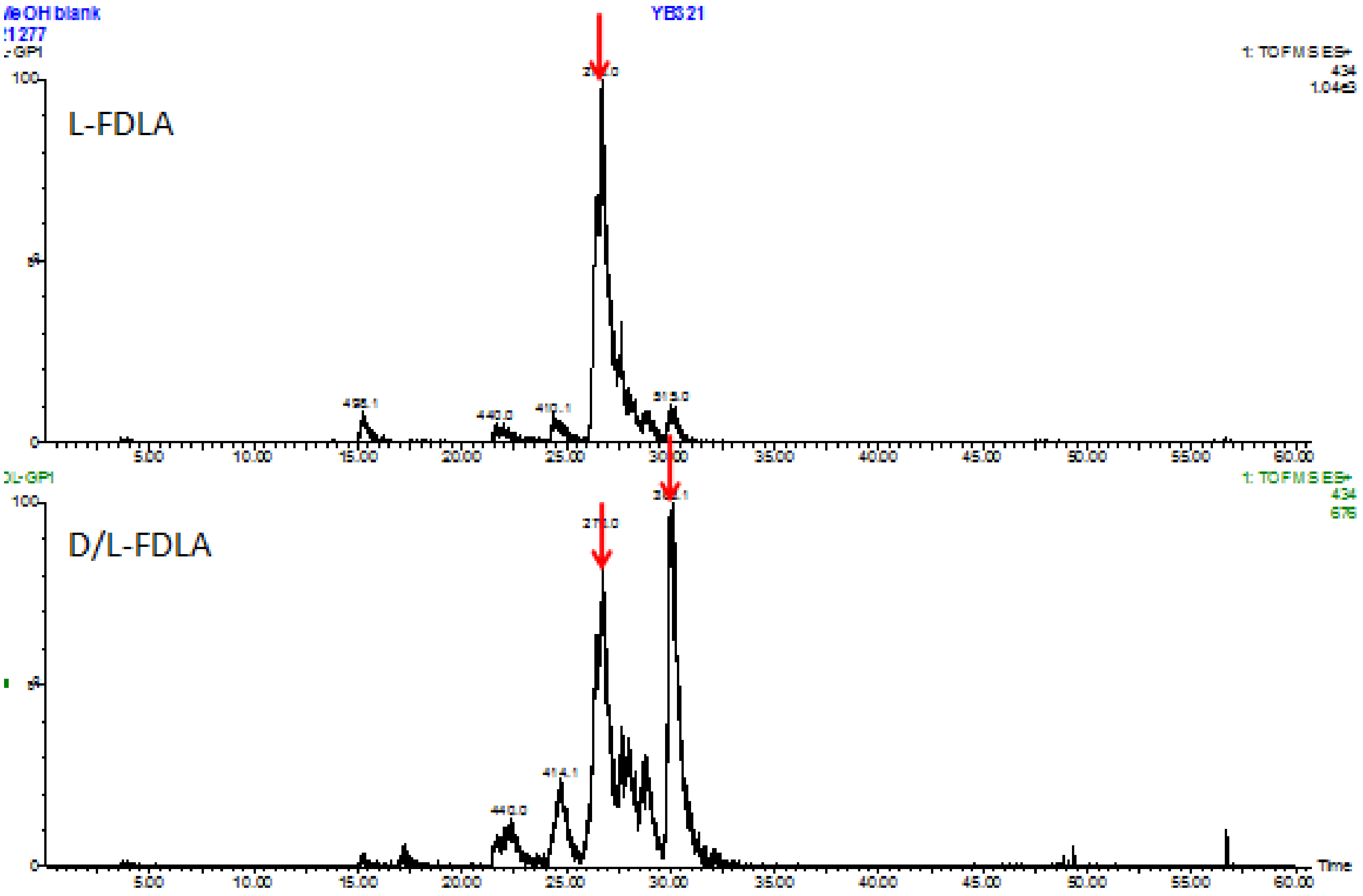

Figure S34.Advanced Marfey's analysis of acid hydrolysate of **2**

A)D,L-FDLA-Threonine derivatives in **2**: 414 $[M+H]^+$

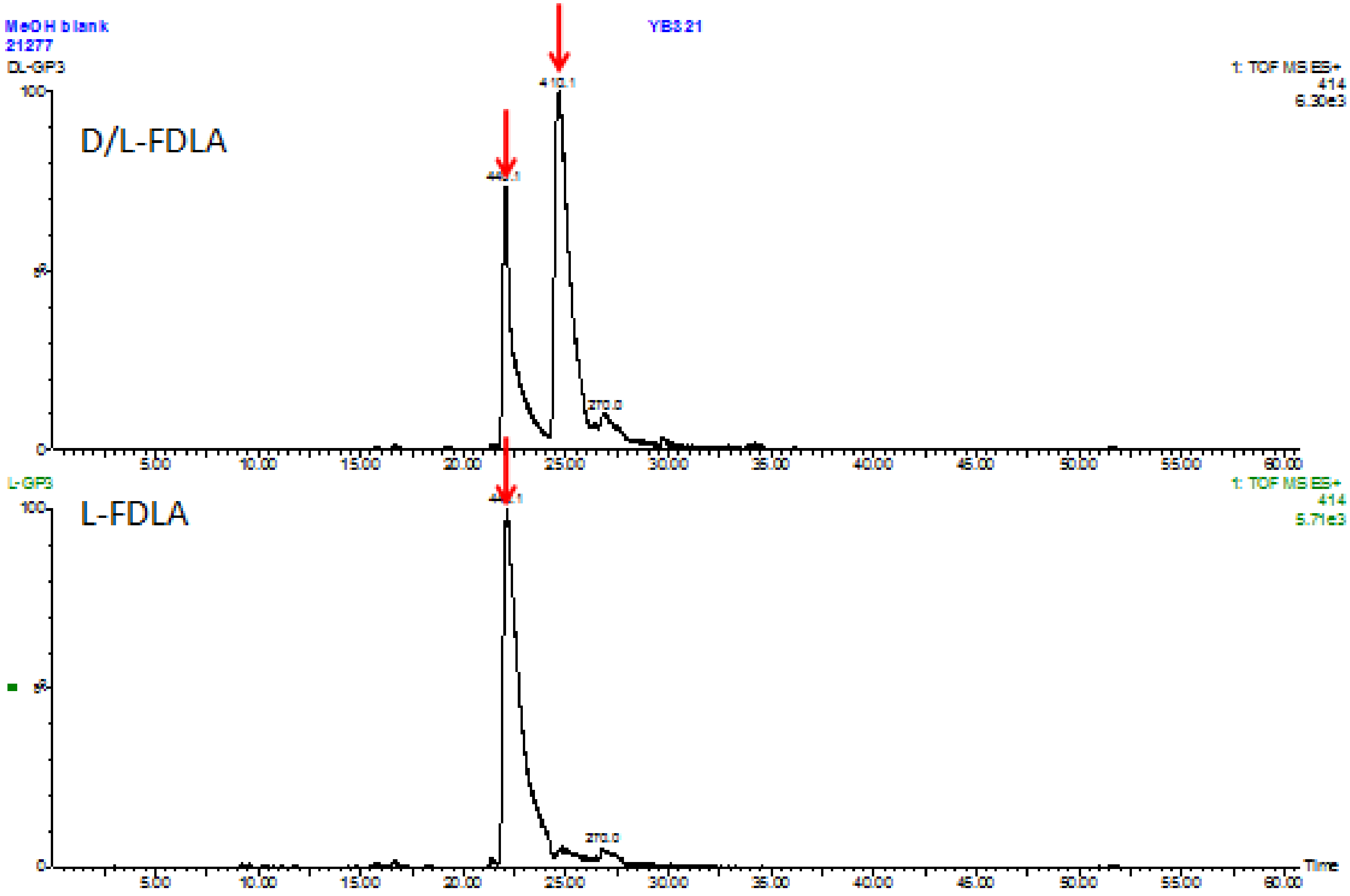

B)D,L-FDLA-Leucine derivatives in **2**: 426 $[M+H]^+$

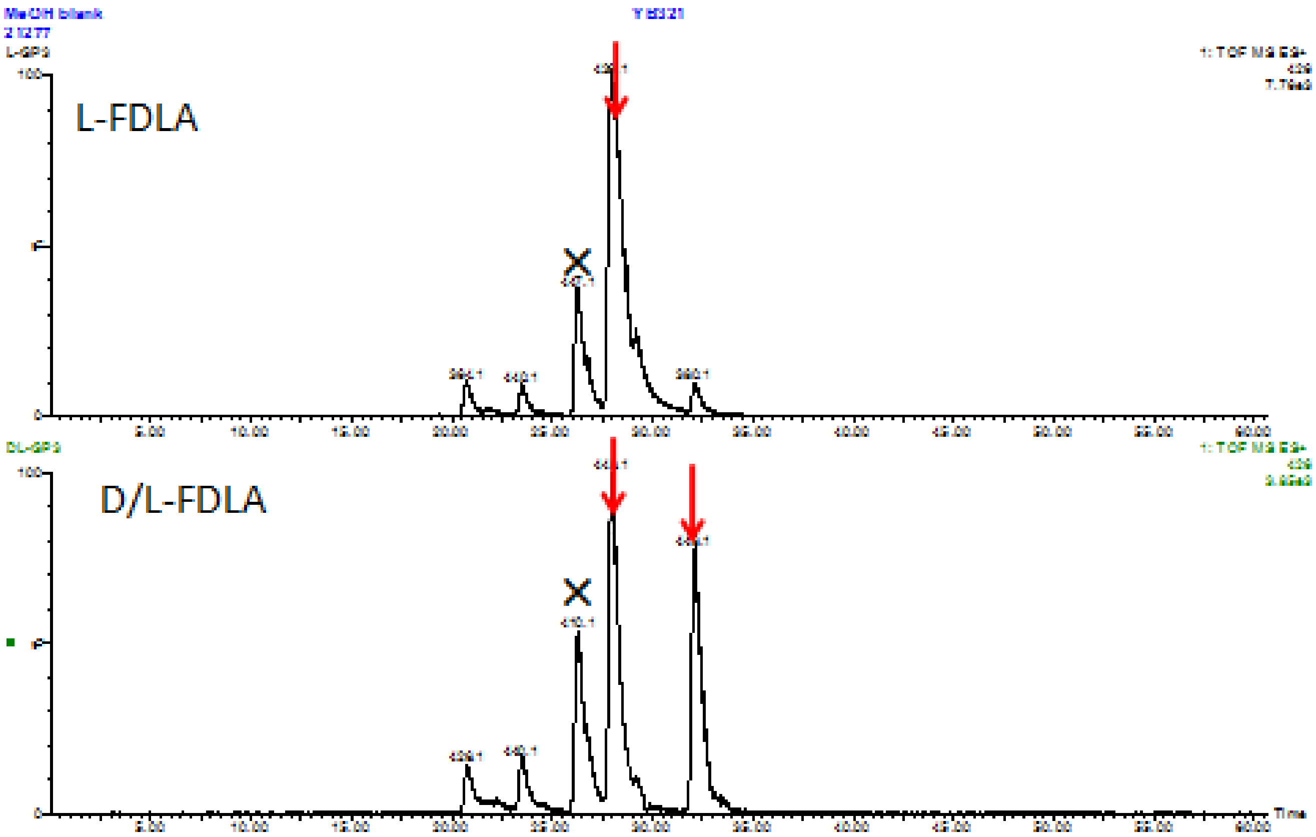

C) D,L-FDLA-HMP derivatives in **2**: 440 $[M+H]^+$

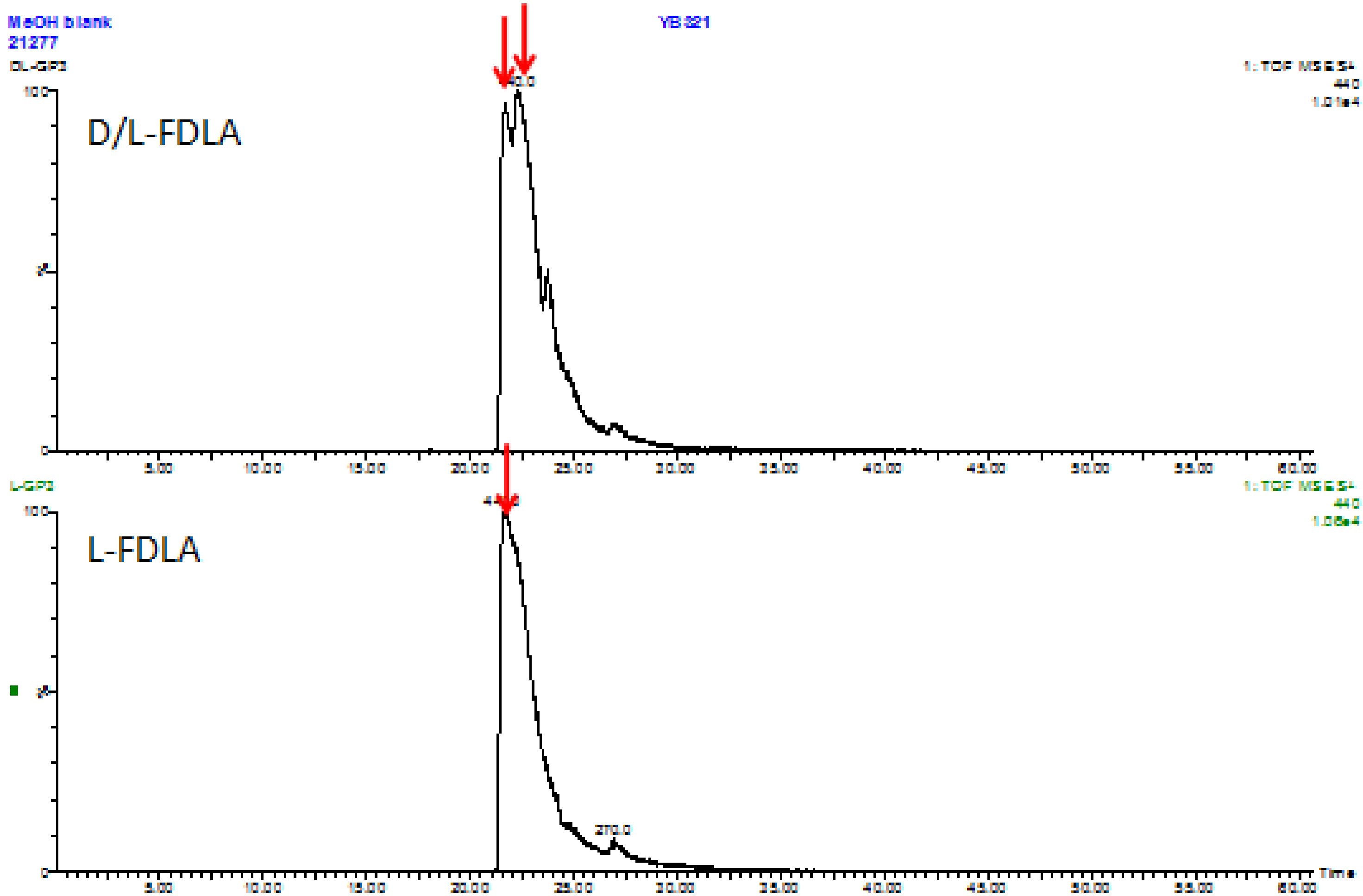

D) D,L-FDLA-Valine derivatives in **2**: 434 $[M+Na]^+$

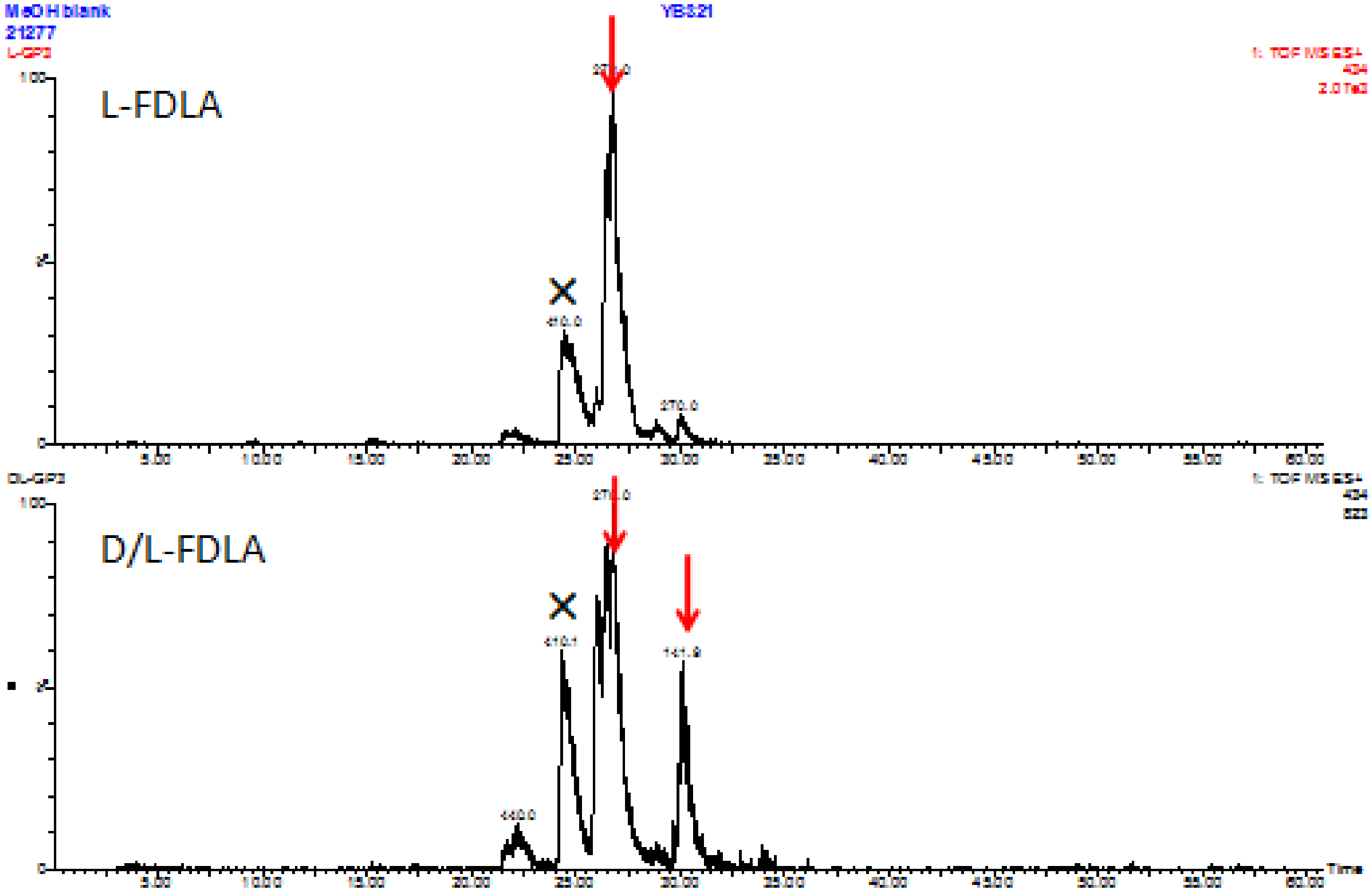

E) D,L-FDLA-Proline derivatives in **2**: 410 $[M+H]^+$

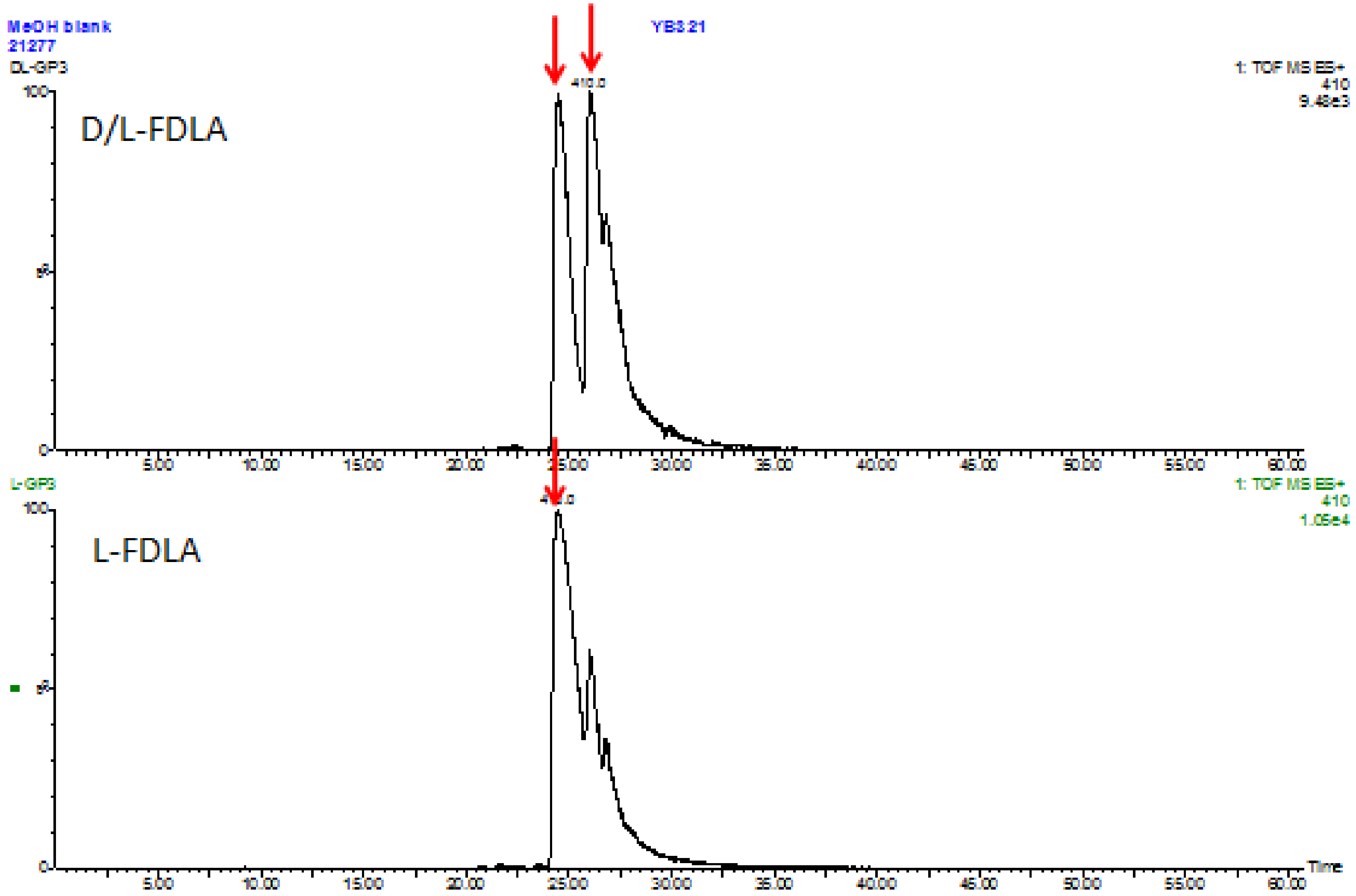

F) D,L-FDLA-Proline standard: 410 $[M+H]^{+}$

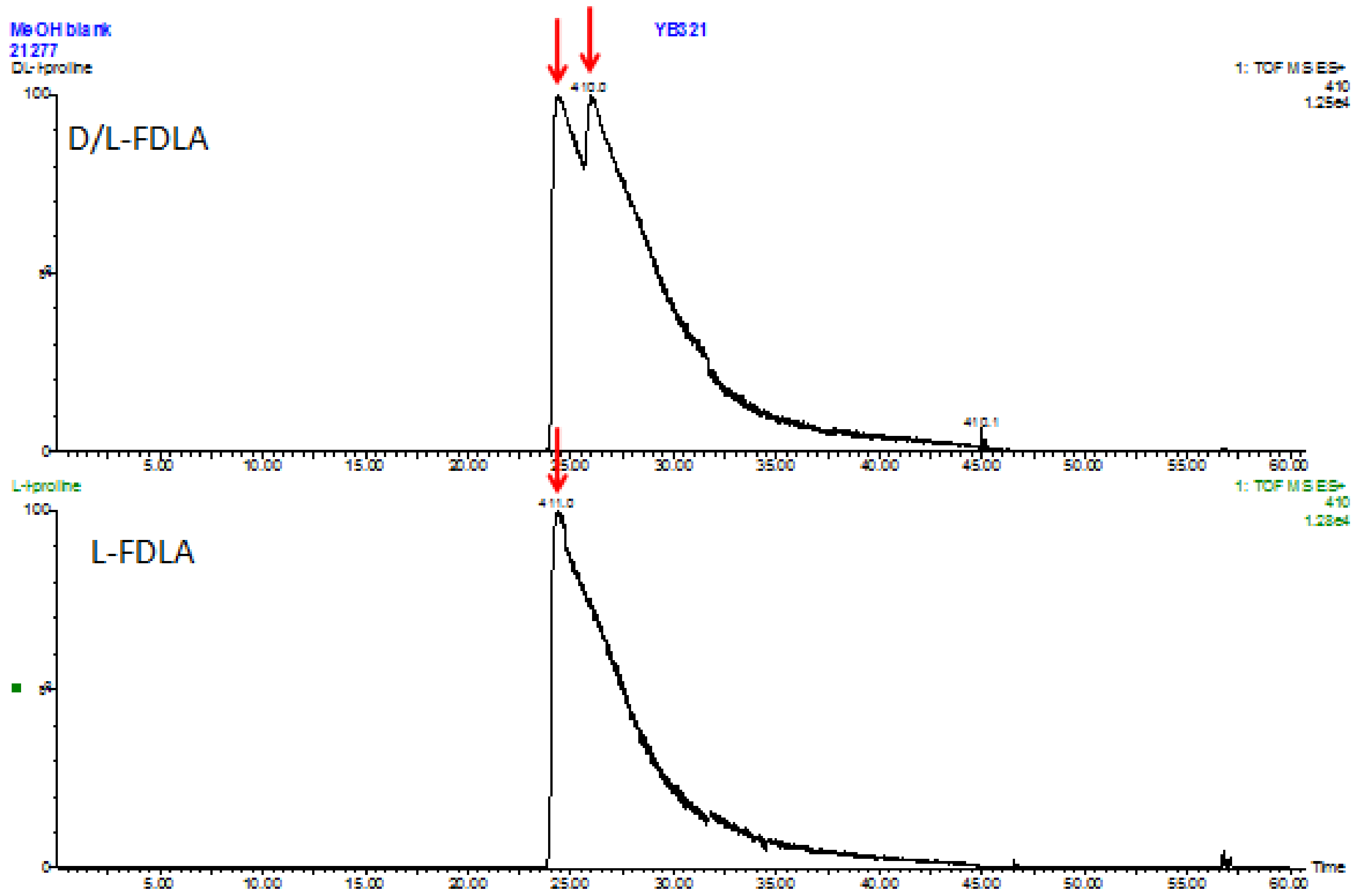

Figure S35. Advanced Marfey's analysis of acid hydrolysate of **3**

A)D,L-FDLA-Threonine derivatives in **3**: 414 $[M+H]^+$

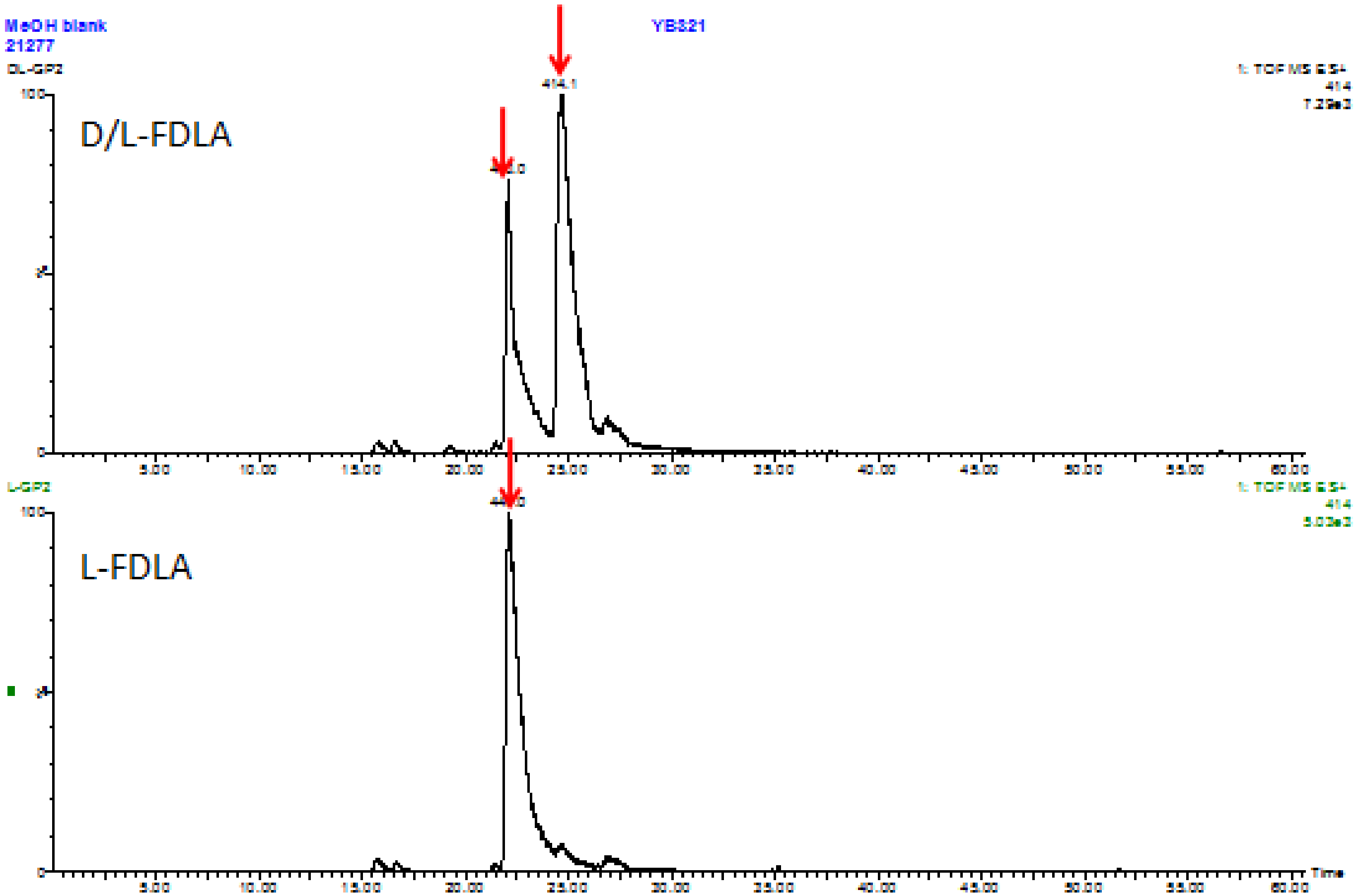

B)D,L-FDLA-Leucine derivatives in **3**: 426 $[M+H]^+$

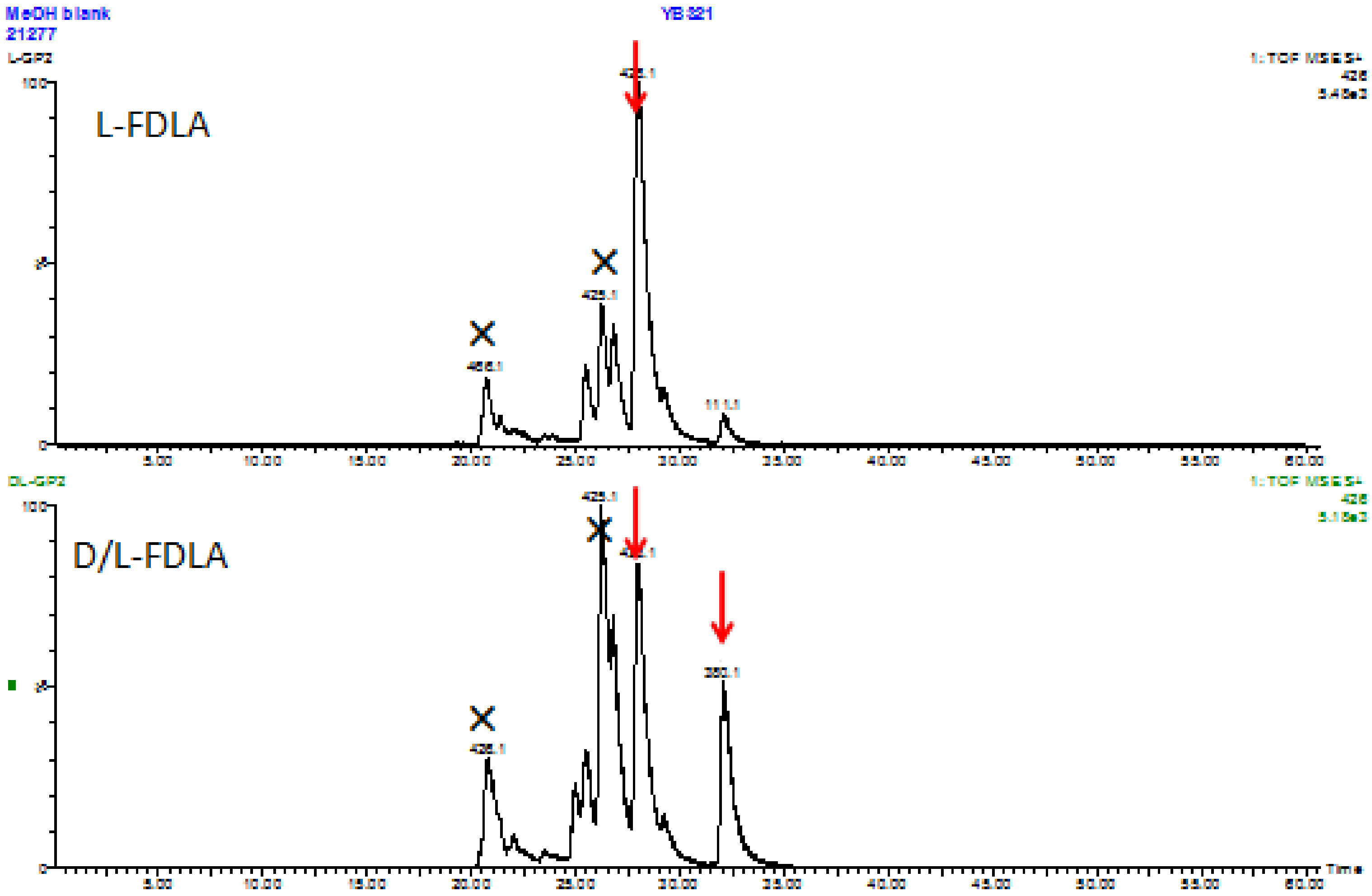

C) D,L-FDLA-HMP derivatives in **3**: 440 $[M+H]^+$

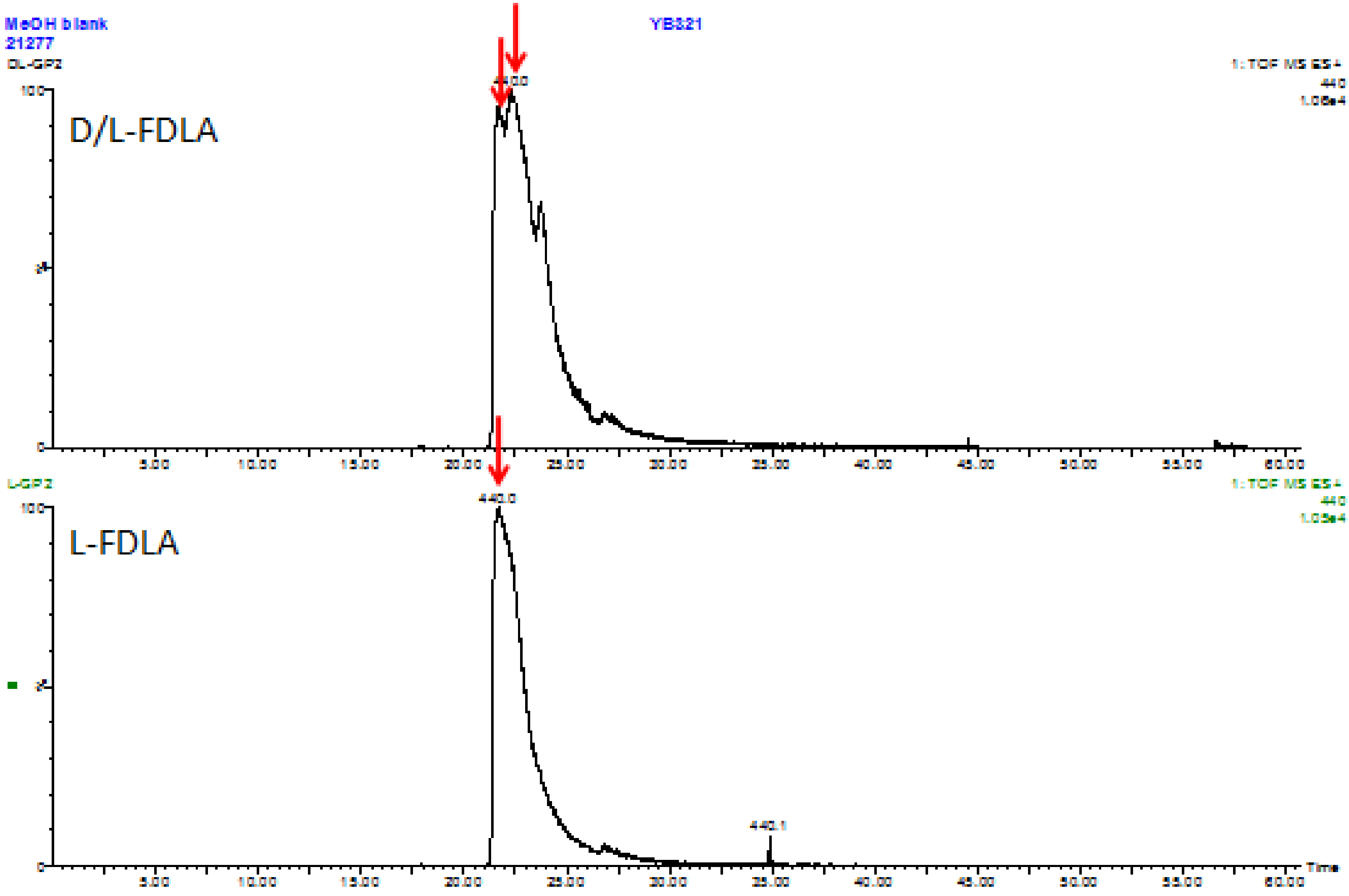

D) D,L-FDLA-Valine derivatives in **3**: 434 $[M+Na]^+$

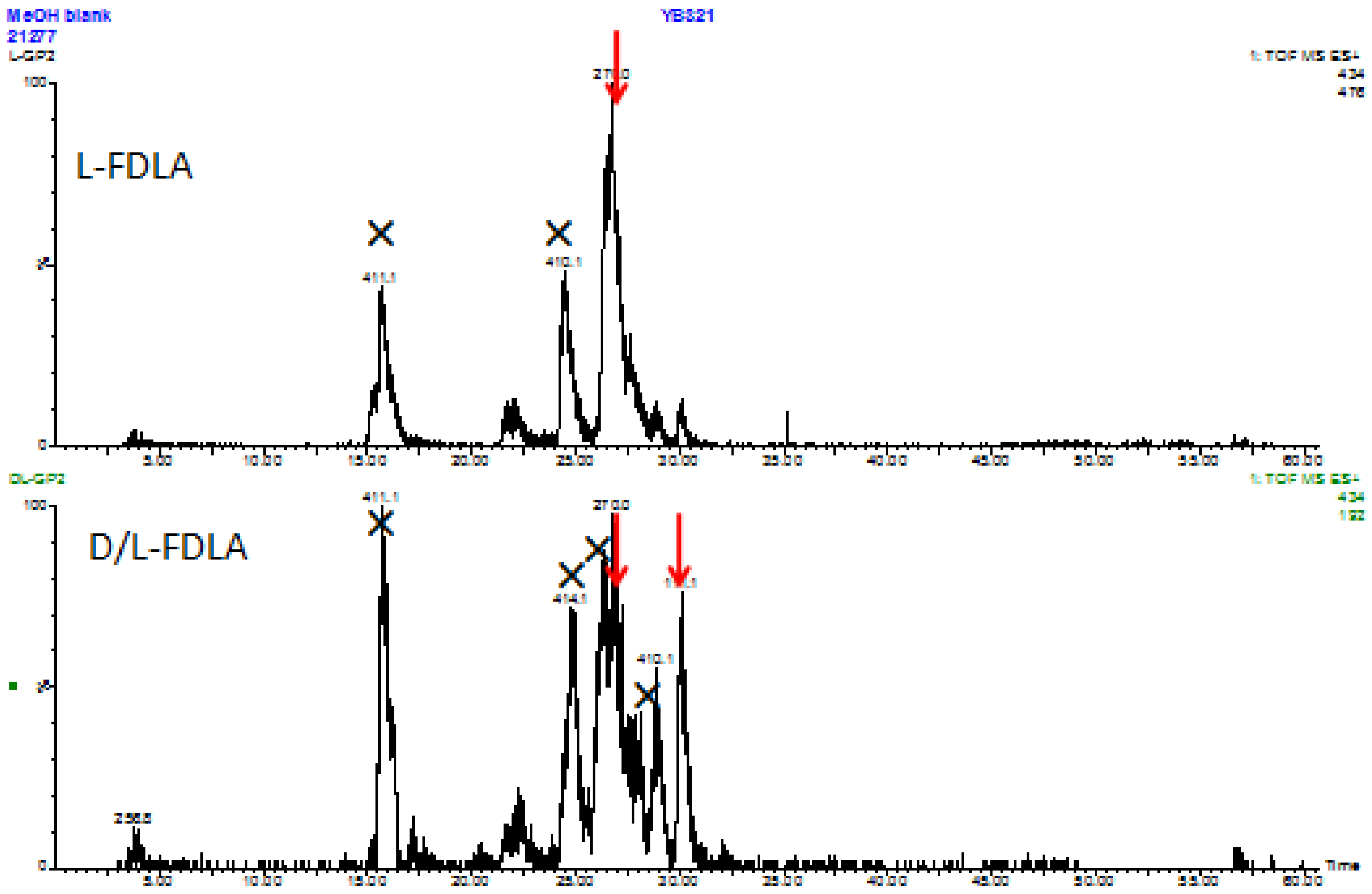

E) D,L-FDLA-Proline derivatives in **3**: 410 $[M+H]^+$

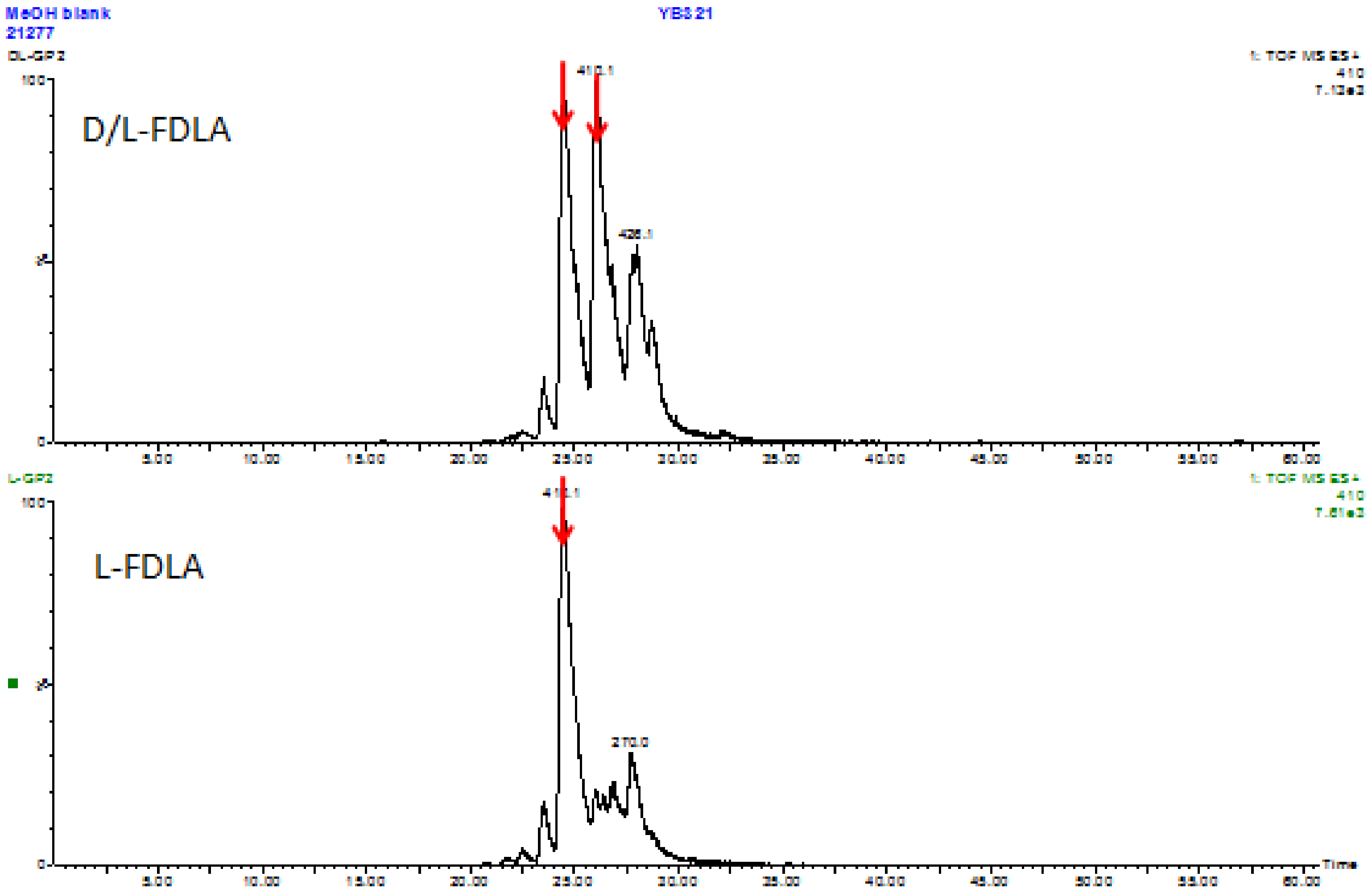

Table S1: Corresponding Retention times between D/L-FDLA derivatives of amino acids

Amino acids	Structure of FDLA-derivatives	m/z $[M+H]^+$	Retention time of D/L-FDLA derivatives (min)	Retention time of L-FDLA derivatives (min)
L-Threonine		414	22.03, 24.57	22.08
L-Leucine		426	27.94, 32.03	28.03
		440.1	21.61, 22.22	21.59
L-Valine		412, 434[M+Na]+	26.69, 29.95	26.69
L-Proline		410	24.41, 26.00	24.35
L-Proline (standard)		410	24.43, 26.05	24.39

Analysis condition:

HPLC-MS method: the analysis of the L- and D-FDLA derivatives was carried out by an Agilent Eclipse XDB-C18 column (150×4.6 mm, 5 μm) employing a linear gradient of from 5% to 100% CH_3CN in 0.1%formic acid at 0.5 mL/min over 45 min.
